# TPC2 rescues lysosomal storage in mucolipidosis type IV, Niemann–Pick type C1, and Batten disease

**DOI:** 10.15252/emmm.202115377

**Published:** 2022-08-05

**Authors:** Anna Scotto Rosato, Einar K Krogsaeter, Dawid Jaślan, Carla Abrahamian, Sandro Montefusco, Chiara Soldati, Barbara Spix, Maria Teresa Pizzo, Giuseppina Grieco, Julia Böck, Amanda Wyatt, Daniela Wünkhaus, Marcel Passon, Marc Stieglitz, Marco Keller, Guido Hermey, Sandra Markmann, Doris Gruber‐Schoffnegger, Susan Cotman, Ludger Johannes, Dennis Crusius, Ulrich Boehm, Christian Wahl‐Schott, Martin Biel, Franz Bracher, Elvira De Leonibus, Elena Polishchuk, Diego L Medina, Dominik Paquet, Christian Grimm

**Affiliations:** ^1^ Faculty of Medicine, Walther Straub Institute of Pharmacology and Toxicology Ludwig‐Maximilians‐Universität Munich Germany; ^2^ Telethon Institute of Genetics and Medicine Naples Italy; ^3^ Experimental Pharmacology, Center for Molecular Signaling (PZMS) Saarland University School of Medicine Homburg Germany; ^4^ Evotec AG Hamburg Germany; ^5^ Department of Pharmacy, Center for Drug Research Ludwig‐Maximilians‐Universität Munich Germany; ^6^ Center for Molecular Neurobiology Hamburg (ZMNH) Institute of Molecular and Cellular Cognition, UKE Hamburg Germany; ^7^ Department of Neurology, Center for Genomic Medicine Massachusetts General Hospital, Harvard Medical School Boston MA USA; ^8^ Cellular and Chemical Biology Department, Institut Curie U1143 INSERM, UMR3666 CNRS, PSL Research University Paris France; ^9^ Institute for Stroke and Dementia Research (ISD) Ludwig‐Maximilians‐University (LMU) Hospital Munich Germany; ^10^ Institute for Neurophysiology Hannover Medical School Hannover Germany; ^11^ Institute of Biochemistry and Cell Biology (IBBC), CNR Rome Italy; ^12^ Medical Genetics Unit, Department of Medical and Translational Science Federico II University Naples Italy; ^13^ Munich Cluster for Systems Neurology (SyNergy) Ludwig‐Maximilians‐University (LMU) Munich Germany

**Keywords:** Batten, MLIV, NPC1, TPC2, TRPML, Genetics, Gene Therapy & Genetic Disease, Organelles, Pharmacology & Drug Discovery

## Abstract

Lysosomes are cell organelles that degrade macromolecules to recycle their components. If lysosomal degradative function is impaired, e.g., due to mutations in lysosomal enzymes or membrane proteins, lysosomal storage diseases (LSDs) can develop. LSDs manifest often with neurodegenerative symptoms, typically starting in early childhood, and going along with a strongly reduced life expectancy and quality of life. We show here that small molecule activation of the Ca^2+^‐permeable endolysosomal two‐pore channel 2 (TPC2) results in an amelioration of cellular phenotypes associated with LSDs such as cholesterol or lipofuscin accumulation, or the formation of abnormal vacuoles seen by electron microscopy. Rescue effects by TPC2 activation, which promotes lysosomal exocytosis and autophagy, were assessed in mucolipidosis type IV (MLIV), Niemann–Pick type C1, and Batten disease patient fibroblasts, and in neurons derived from newly generated isogenic human iPSC models for MLIV and Batten disease. For *in vivo* proof of concept, we tested TPC2 activation in the MLIV mouse model. In sum, our data suggest that TPC2 is a promising target for the treatment of different types of LSDs, both *in vitro* and *in‐vivo*.

## Graphical abstract

Mutations in proteins of the endolysosomal machinery such as lysosomal enzymes or lysosomal membrane proteins often result in severe neurodegenerative disease, including mucolipidosis type IV, Niemann–Pick type C1, or Batten disease.

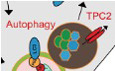

**The paper explained**

**Problem**

Batten disease (JNCL), mucolipidosis type IV (MLIV), and Niemann–Pick type C1 (NPC1) are fatal neurodegenerative rare lysosomal storage diseases. There is currently no curative therapy available for either of these diseases.

**Results**

We show here that treatment with a PI(3,5)P_2_‐mimetic small molecule agonist of the endolysosomal cation channel TPC2, TPC2‐A1‐P, ameliorates cellular disease phenotypes in patient fibroblasts and iPSC‐derived neuronal models of MLIV, NPC1, and JNCL as well as disease phenotypes in the mouse model of MLIV *in vivo*.

**Impact**

Our data suggest that activation of TPC2 has the potential to serve as a novel approach to treat different lysosomal storage disorders, in particular those going along with a disturbed endolysosomal Ca^2+^ homeostasis.

## Introduction

Lysosomal Ca^2+^ release is of significant physiological relevance. Lysosomal Ca^2+^ regulates several cellular processes, e.g., autophagy (Medina *et al*, 2015), membrane trafficking (Dong *et al*, 2010; Ruas *et al*, 2010; Cao *et al*, 2015), exocytosis (Samie *et al*, 2013; Davis *et al*, 2020), nutrient adaptation (Cang *et al*, 2013), membrane repair (Cheng *et al*, 2014), and cell migration (Bretou *et al*, 2017). Disruption of lysosomal Ca^2+^ content or Ca^2+^ release is associated with several diseases, particularly neurodegenerative lysosomal storage diseases (Kiselyov *et al*, 2010; Lloyd‐Evans & Platt, 2011; Feng & Yang, 2016). Mucolipidosis type IV (MLIV) constitutes the most direct link between defective lysosomal Ca^2+^ release and neurodegeneration, caused by dysfunction of the lysosomal cation channel TRPML1 (also called MCOLN1) (Slaugenhaupt, 2002; Feng & Yang, 2016). TRPML1 signaling or TRPML1‐mediated Ca^2+^ release is similarly impaired in other LSDs such as Niemann–Pick type C1 (NPC1) (Shen *et al*, 2012), Niemann–Pick type A (NPA; also called infantile neurovisceral form of acid sphingomyelinase (SMPD1) deficiency) (Zhong *et al*, 2016), and Fabry disease (Zhong *et al*, 2016). Pharmacological and genetic activation of TRPML1 ameliorates NPC1‐associated lactosylceramide (LacCer) trafficking defects and cholesterol accumulation (Shen *et al*, 2012), while activation of the lysosomal big conductance Ca^2+^‐activated potassium (BK) channel TRPML1 dependently rescues aberrant lysosomal storage in NPA and Fabry disease (Zhong *et al*, 2016). Furthermore, loss of FIG 4 (polyphosphoinositide phosphatase) and PYKfyve (FYVE finger‐containing phosphoinositide kinase), which are both involved in the synthesis of the endogenous TRPML/two‐pore channel (TPC) agonist PI(3,5)P_2_ (phosphatidylinositol 3,5‐bisphosphate), is associated with neurological or neurodegenerative disease phenotypes (Chow *et al*, 2007; Zhang *et al*, 2007; Zou *et al*, 2015), and TRPML1 activation in FIG 4^−/−^ cells rescues lysosomal storage phenotypes (Zou *et al*, 2015).

While activation of TRPML1 in LSDs is gaining traction, effects of activating the related two‐pore channel 2 (TPC2 or TPCN2) remain unexplored. TPC2 shares several features with TRPML1: both channels are permeable for Ca^2+^ and Na^+^ (Calcraft *et al*, 2009; Zong *et al*, 2009; Wang *et al*, 2012; Gerndt *et al*, 2020), reside in endolysosomal membranes (Pryor *et al*, 2006; Calcraft *et al*, 2009; Kim *et al*, 2009; Ruas *et al*, 2010), are activated by PI(3,5)P_2_ (Dong *et al*, 2010; Wang *et al*, 2012; Gerndt *et al*, 2020), are widely expressed in the CNS (Bae *et al*, 2014; Pereira *et al*, 2017; Foster *et al*, 2018; Minckley *et al*, 2019), cause trafficking defects when lost (Dong *et al*, 2010; Shen *et al*, 2012; Chen *et al*, 2014; Grimm *et al*, 2014; Nguyen *et al*, 2017), interact with mTOR/TFEB/autophagy pathways (Medina *et al*, 2011; Cang *et al*, 2013; Medina *et al*, 2015; Wang *et al*, 2015; Li *et al*, 2016; Ogunbayo *et al*, 2018; Scotto Rosato *et al*, 2019), and promote lysosomal exocytosis (Medina *et al*, 2011; Samie *et al*, 2013; Gerndt *et al*, 2020).

We therefore hypothesized that TPC2 activation may modulate lysosomal Ca^2+^ signaling to rescue LSD phenotypes, particularly in LSDs where TRPML1 is impacted. In our study, we focused on MLIV and NPC1 on the one hand, LSDs that both have been shown before to be connected to disrupted lysosomal Ca^2+^ signaling and TRPML1 dysfunction (Shen *et al*, 2012). On the other hand, we focused on juvenile neuronal ceroid lipofuscinosis (JNCL) or Batten disease, caused by mutations in CLN3, an LSD which shows prominent retinal and neurodegenerative phenotypes with gradual vision loss and progressive cognitive decline as observed in MLIV, and with a similar age‐dependent disease onset and evidence for disturbed lysosomal Ca^2+^ homeostasis (Chandrachud *et al*, 2015). By analyzing disease hallmarks in patient fibroblasts, novel CRISPR/Cas9‐engineered iPSCs/iPSC‐derived neurons, and the MLIV mouse upon treatment with a TPC2 small molecule agonist, TPC2‐A1‐P, we demonstrate that TPC2 activation ameliorates the phenotypes of these LSDs both *in vitro* and *in vivo*.

## Results

### TPC2 activation modulates LSD phenotypes in human patient fibroblasts

**Figure 1:**
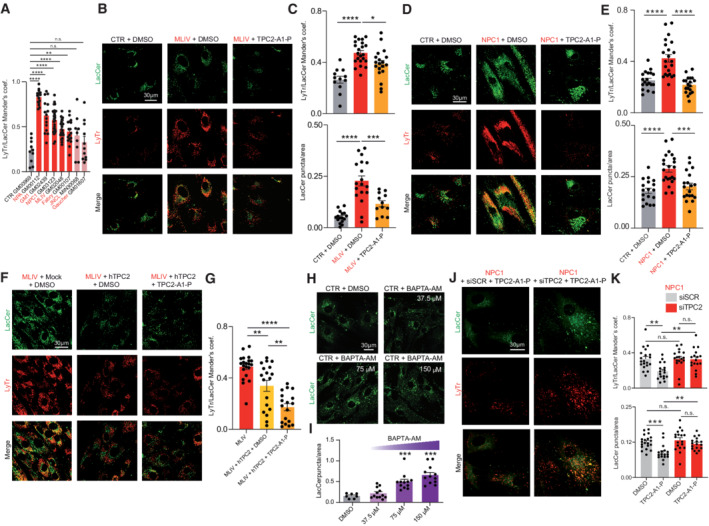
TPC2 agonist effect on lactosylceramide trafficking AColocalization of LacCer and LysoTracker (LyTr) in different CTR and LSD patient fibroblasts. Mander's coefficients were calculated using the Fiji JACoP plugin.B, CConfocal images (B) and statistical analysis (C) showing colocalization of LacCer and LyTr in human CTR and MLIV fibroblasts, treated with TPC2‐A1‐P (30 μM, 16 h).D, EConfocal images (D) and statistical analysis (E) showing colocalization of LacCer and LyTr in human CTR and NPC1 fibroblasts, treated with TPC2‐A1‐P (30 μM, 48 h).F, GConfocal images and statistical analysis showing LacCer/LyTr colocalization in MLIV patient fibroblasts which were moc ‐electroporated and treated with DMSO or electroporated with a gain‐of‐function hTPC2(M484L/G734E):mCherry TOPO 3.1 vector and treated with either DMSO or TPC2‐A1‐P (30 μM, 16 h).H, ICa^2+^ chelation (BAPTA‐AM) dose dependently impairs LacCer trafficking in CTR fibroblasts.J, KConfocal images (J) and statistical analysis (K) of NPC1 patient fibroblasts treated with 50 nM mock siRNA (siSCR) or siRNA targeting TPCN2 (siTPC2) for 72 h. Cells were then treated with DMSO or TPC2‐A1‐P (30 μM). Data information: Shown are mean values ± SEM. *n* > 3 technical and biological replicates for each tested condition (each dot represents an imaged frame containing several cells); one‐way ANOVA, *post hoc* Bonferroni's (A, C, E, G, I) or Tukey's (K) multiple comparisons test. *p‐value < 0.05; **p‐value < 0.01; ***p‐value < 0.001; ****p‐value < 0.0001.

**Figure 2:**
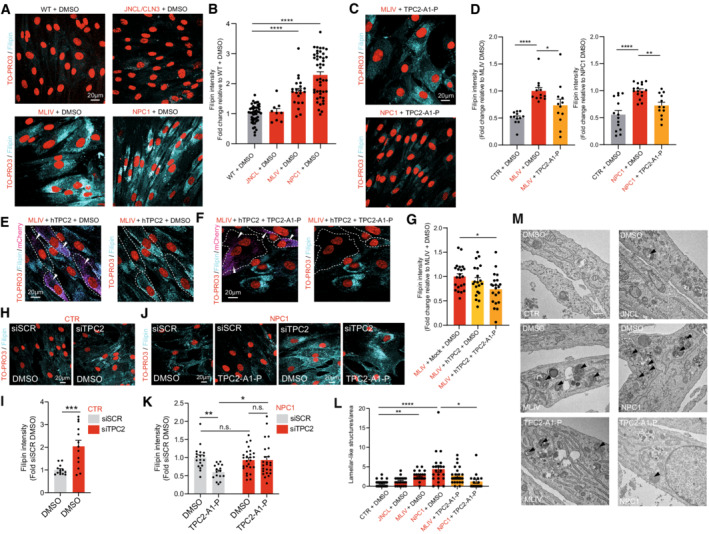
TPC2 agonist effect on cholesterol accumulation and ultrastructural changes A, BConfocal images (A) and statistical analysis (B) of cholesterol accumulation in human CTR, MLIV, JNCL, and NPC1 fibroblasts. Cholesterol accumulation was evident for NPC1 and MLIV fibroblasts but not for JNCL fibroblasts. The images show filipin staining to visualize cholesterol accumulation and TO‐PRO3 as nuclear staining.C, DTPC2‐A1‐P (30 μM, 48 h) rescued NPC1 and MLIV cholesterol accumulation.E–GConfocal images (E‐F) and statistical analysis (G) of MLIV patient fibroblasts mock electroporated and treated with DMSO or electroporated with a gain‐of‐function hTPC2(M484L/G734E):mCherry TOPO 3.1 vector (white arrowheads) and treated with either DMSO or TPC2‐A1‐P (30 μM, 48 h).H–KConfocal images (H‐I) and statistical analysis (J‐K) of human CTR and NPC1 patient fibroblasts treated with 50 nM mock siRNA (siSCR) or siRNA targeting TPCN2 (siTPC2) for 72 h. Cells were then treated with DMSO or TPC2‐A1‐P (30 μM).L, MStatistics (L) and electron microscopy images (M) of human CTR, MLIV, JNCL, and NPC1 fibroblasts. The effect of the treatment with TPC2 agonist (30 μM, 48 h) was examined in NPC1 and MLIV cells. Data information: Shown are mean values ± SEM. *n* > 3 technical and biological replicates for each tested condition (each dot represents an imaged frame containing several cells); one‐way ANOVA, *post hoc* Bonferroni's multiple comparisons test (B, D, E, L), or two‐tailed Student's *t*‐test (J and K). *p‐value < 0.05; **p‐value < 0.01; ***p‐value < 0.001; ****p‐value < 0.0001.

**Figure 3:**
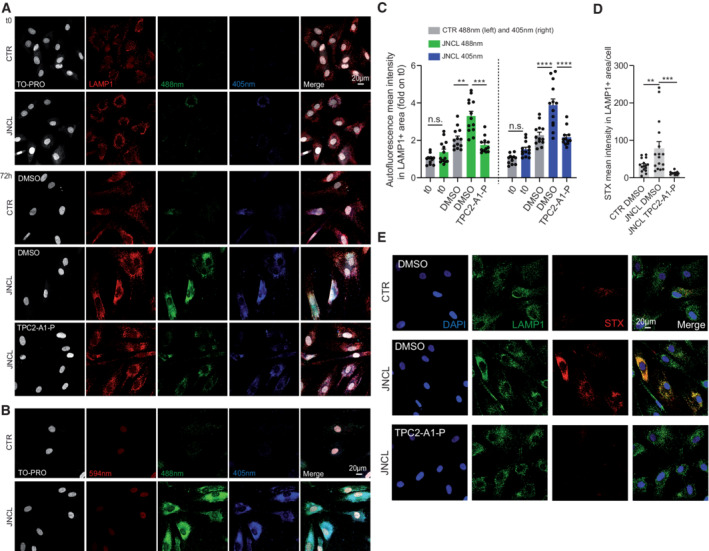
TPC2 agonist effect on lipofuscin and Gb3 accumulation AConfocal images of CTR and JNCL fibroblasts. Images show LAMP1 staining and autofluorescence at 405 and 488 nm excitation wavelength, respectively, corresponding to the lipofuscin autofluorescence spectrum. Cells were treated with DMSO or TPC2‐A1‐P (30 μM) following cell cycle arrest (2 h mitomycin C treatment).BConfocal images showing no autofluorescence signal at 594 nm excitation wavelength (used for LAMP1 staining).CMean autofluorescence intensity in LAMP1^+^ area.D, EConfocal images of Gb3 accumulation stained with Shiga toxin (STX) in CTR and JNCL fibroblasts. Cells were treated with DMSO or TPC2‐A1‐P (30 μM) after cell cycle arrest. Data information: Shown are mean values ± SEM. *n* > 3 technical and biological replicates for each tested condition (each dot represents an imaged frame containing several cells); one‐way ANOVA, *post hoc* Bonferroni's multiple comparisons test. **p‐value < 0.01; ***p‐value < 0.001; ****p‐value < 0.0001.

**Figure EV1:**
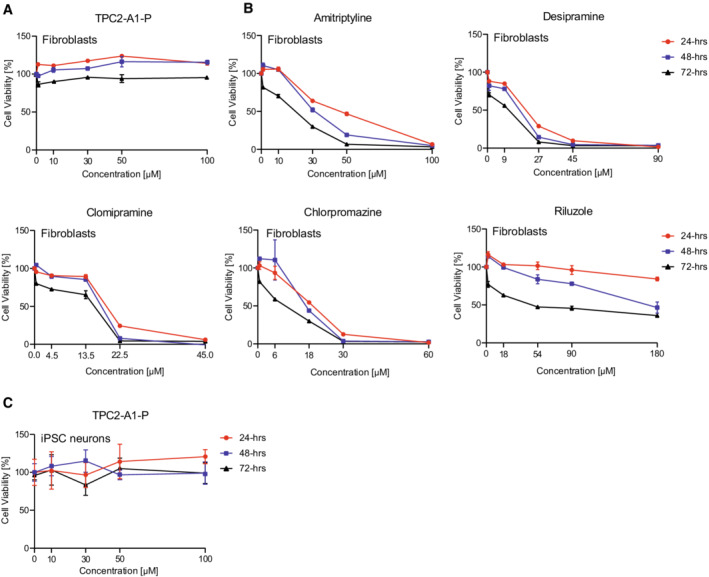
Effect of TPC2‐A1‐P and various drugs reported to activate TPC2 on cell viability A–CCell viability assay for TPC2‐A1‐P and other compounds reported to activate TPC2 (Zhang *et al*, 2019) on human patient fibroblasts (A, B) and iPSC‐derived neurons (C). Cells were incubated for 24, 48, and 72 h with increasing compound concentrations, and cell viability was assessed with CellTiter‐Blue according to the manufacturer's protocol. Data are presented as mean ± SEM. *n* > 3 for each tested condition.

**Figure EV2:**
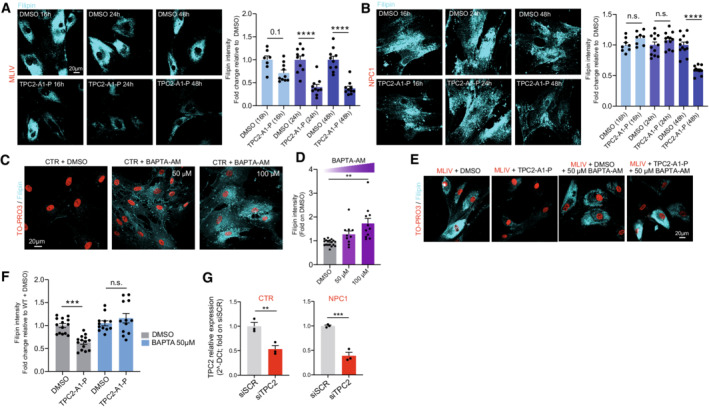
Time course of filipin rescue and effect of BAPTA‐AM in human fibroblasts A, BTime course of filipin rescue (treatment with either DMSO or 30 μM TPC2‐A1‐P) in human MLIV and NPC1 fibroblasts (16–48 h).C, DCa^2+^ chelation (BAPTA‐AM) dose dependently causes cholesterol accumulation in CTR fibroblasts.E, FCa^2+^ chelation (BAPTA‐AM) blunts the effect of TPC2‐A1‐P (48 h treatment) when added for the last 3 h.GRT‐qPCR showing TPCN2 knockdown efficiency in human CTR and NPC1 fibroblasts. Data information: Shown are mean values ± SEM. *n* > 3 technical and biological replicates for each tested condition (each dot represents an imaged frame containing several cells or three independent qPCR experiments, respectively); one‐way (A, B) or two‐way (F) ANOVA, *post hoc* Tukey's multiple comparisons test, or two‐tailed Student's *t*‐test (D, G). **p‐value < 0.01; ***p‐value < 0.001; ****p‐value < 0.0001.

Based on the concept that disrupted endolysosomal Ca^2+^ homeostasis constitutes a major pathomechanism underlying LSDs as evidenced by MLIV, we assessed the effect of our recently published PI(3,5)P_2_‐mimetic TPC2 agonist, TPC2‐A1‐P (Gerndt *et al*, 2020), releasing both Ca^2+^ and Na^+^, on the phenotypes of the above‐mentioned LSDs. For NPC1 and MLIV, lactosylceramide (LacCer) and cholesterol trafficking defects are reported (Shen *et al*, 2012; Chen *et al*, 2014). Hence, we started our study by assessing these defects in fibroblasts from NPC1 and MLIV patients compared to control (CTR) fibroblasts. The lipid LacCer is internalized clathrin independently and targeted to the Golgi apparatus in CTR cells, whereas in several LSD fibroblasts including NPC1 and MLIV it accumulates in late endosomes and lysosomes. Accordingly, we observed significant endolysosomal accumulation of LacCer in NPC1 and MLIV, and a range of other LSDs compared to CTR, but not for JNCL (CLN3^Δ1.02kb/Δ1.02kb^) and Gaucher, as reported previously (Vitner *et al*, 2010), demonstrating reproducibility of the assay (Fig 1A). We next assessed the effect of TPC2 activation in MLIV and NPC1 versus CTR fibroblasts. In MLIV fibroblasts, carrying the most common patient variation (MCOLN1^IVS3‐2A>G/Ex1‐7del^; GM02048) TPC2 activation by TPC2‐A1‐P significantly reduced lysosomal accumulation of LacCer (Mander's coefficient) and the number of LacCer puncta per area after incubation overnight (16 h) (Fig 1B and C), while in NPC1 cells (NPC1^P237S/I1061T^; GM03123), significant rescue was seen after 48 h incubation (Fig 1D and E). To assess maximal rescue effects, we tested overexpression of a gain‐of‐function variant of TPC2 (TPC2^M484L/G734E^; Chao *et al*, 2017) with and without TPC2‐A1‐P activation in MLIV fibroblasts (Fig 1F and G). Both TPC2 overexpression alone and overexpression in combination with TPC2‐A1‐P significantly reduced lysosomal accumulation of LacCer in MLIV cells, with a stronger effect seen in the combination. To exclude any potential toxic effects of TPC2‐A1‐P on fibroblasts, cell viability assays were performed (Fig EV1A). Commercially available drugs reported to activate TPC2 were examined alongside TPC2‐A1‐P (Zhang *et al*, 2019). In these tests, TPC2‐A1‐P showed no toxicity up to the maximal test concentration (100 μM; Fig EV1B). By using the Ca^2+^ chelator BAPTA‐AM, we could further demonstrate that reduction in free intracellular Ca^2+^ induces a similar LacCer trafficking defect in CTR as in MLIV or NPC1 cells (Fig 1H and I), suggesting a relevant role of Ca^2+^ in the process. Furthermore, TPC2‐A1‐P rescued the lysosomal LacCer accumulation in mock, but not in siTPC2‐treated NPC1 fibroblasts, corroborating the on‐target effect of TPC2‐A1‐P (Fig 1J and K). LacCer trafficking is also affected by intracellular cholesterol levels (Pryor *et al*, 2006; Vitner *et al*, 2010; Shen *et al*, 2012; Chen *et al*, 2014). Cholesterol reduction reportedly restores proper LacCer trafficking to Golgi, whereas cholesterol overload redirects LacCer to endolysosomal compartments (Puri *et al*, 1999). We therefore next assessed endolysosomal cholesterol accumulation, which has been reported for both MLIV and NPC1 (Shen *et al*, 2012; Chen *et al*, 2014; Grimm *et al*, 2014). Altered cellular cholesterol homeostasis can conveniently be visualized using the polyene antibiotic filipin. While we could not detect cholesterol storage in JNCL cells, we could confirm that NPC1 and MLIV fibroblasts strongly accumulate cholesterol (Fig 2A and B). In both NPC1 and MLIV cells, accumulated cholesterol was efficiently reduced upon TPC2 activation with TPC2‐A1‐P (Fig 2C and D). While in MLIV cells, significant effects were seen already after 24 h treatment, again in NPC1 cells only after 48 h effects were significant (Fig EV2A and B). In a further set of experiments, we tested TPC2^M484L/G734E^ overexpression with and without TPC2‐A1‐P activation, finding that only overexpression in combination with the agonist significantly reduced intracellular cholesterol (Fig 2E–G). Using BAPTA‐AM, we could again demonstrate, in analogy to LacCer, that chelation of Ca^2+^ results in cholesterol accumulation (Fig EV2C and D), confirming free intracellular Ca^2+^ to play a role in the process. BAPTA‐AM was also shown to blunt the effect of TPC2‐A1‐P (Fig EV2E and F). We further silenced TPC2 expression in healthy human fibroblasts, which resulted in cholesterol accumulation in siTPC2, but not in mock‐treated cells (Fig 2H and I), in accordance with previous observations in murine TPC2 knockout fibroblasts (Grimm *et al*, 2014). Furthermore, TPC2‐A1‐P rescued the cholesterol accumulation in mock‐treated, but not in siTPC2‐treated NPC1 fibroblasts, corroborating the on‐target effect of TPC2‐A1‐P (Fig 2J and K). Efficacy of the siRNAs was validated using qRT‐PCR (Fig EV2G). We next used electron microscopy (EM) to assess ultrastructural changes following compound treatment. Gross alterations in endolysosomal morphology have previously been reported in MLIV and NPC1 fibroblasts (Garver *et al*, 2000; Vergarajauregui *et al*, 2008). We found an abundance of lysosomes with aberrant/lamellar structures in NPC1 and to a lesser extent in MLIV cells, but observed no changes in lysosomal morphology in JNCL fibroblasts (Fig 2L and M). NPC1 fibroblasts showed a stronger difference from CTR than MLIV fibroblasts and only for the former we found TPC2‐A1‐P treatment to significantly restore ultrastructural morphology (Fig 2L and M). While neither ultrastructural changes nor changes in LacCer trafficking or cholesterol accumulation were detectable in JNCL cells, JNCL patient fibroblasts are known to accumulate lipofuscin—appearing as an autofluorescent green‐to‐yellow pigment under ultraviolet light (Mole *et al*, 2020). We used the cell cycle blocker mitomycin C to exacerbate the progressive storage of lipofuscin within lysosomal compartments in JNCL fibroblasts (Fig 3A–C). Treatment with TPC2‐A1‐P rescued this autofluorescence, decreasing it to CTR levels (Fig 3A–C). Furthermore, we used fluorescently labeled Shiga toxin (STX) to visualize globotriaosylceramide (Gb3) accumulation, a recently reported (Soldati *et al*, 2021) phenotype in JNCL cells, and found that TPC2‐A1‐P rescued Gb3 accumulation significantly (Fig 3D and E). In conclusion, activating TPC2 with TPC2‐A1‐P restores various LSD phenotypes in patient‐derived fibroblasts.

### Generation of human isogenic iPSC models of MLIV and JNCL using CRISPR/Cas9

**Figure 4:**
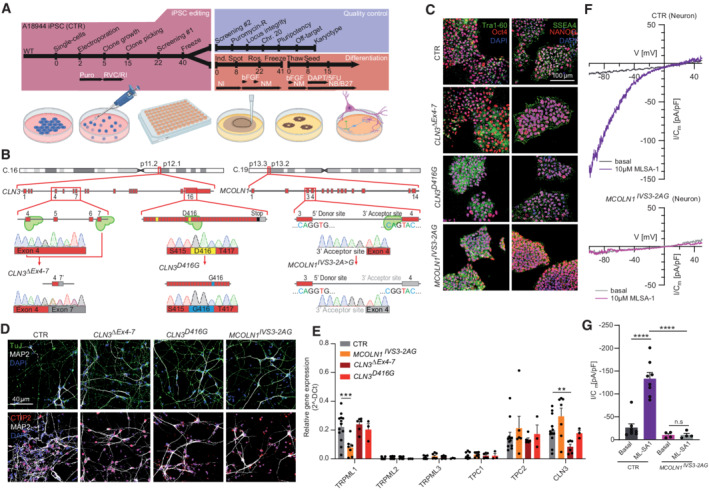
Generation, differentiation, and characterization of lysosomal storage disease iPSCs A, BTimeline of gene editing, quality control, and differentiation. The A18944 iPSC line (CTR) was used for gene editing. iPSCs were electroporated with a plasmid carrying spCas9, target gRNAs, and repair template. Target sites are shown in (B).CImmunofluorescence images of pluripotency markers Tra1‐60, Oct4, SSEA4, and NANOG demonstrate pluripotency of CTR and gene‐edited iPSCs.DEdited iPSCs were differentiated into cortical neurons expressing the neuronal markers TuJ and MAP2, and the cortical neuron transcription factor CTIP2.EUsing RT‐qPCR, we assessed the expression of lysosomal storage disease genes (TRPML1 for MLIV and CLN3 for JNCL) and drug targets (TRPMLs and TPCs).F, GiPSC‐derived neurons were treated with apilimod to enlarge lysosomes, and TRPML1 responsiveness was assessed. ML‐SA1 (10 μM)‐elicited TRPML1 currents were observed in CTR lysosomes but not in MLIV neurons, indicative of abrogated TRPML1 function. Data information: Shown are mean values ± SEM. *n* > 3 technical and biological replicates for each tested condition (each dot represents a single measurement from distinct neuronal differentiations); Gaussian distribution assumed; one‐way ANOVA, followed by Tukey *post hoc* test. **p‐value < 0.01; ****p‐value < 0.0001.

**Figure EV3:**
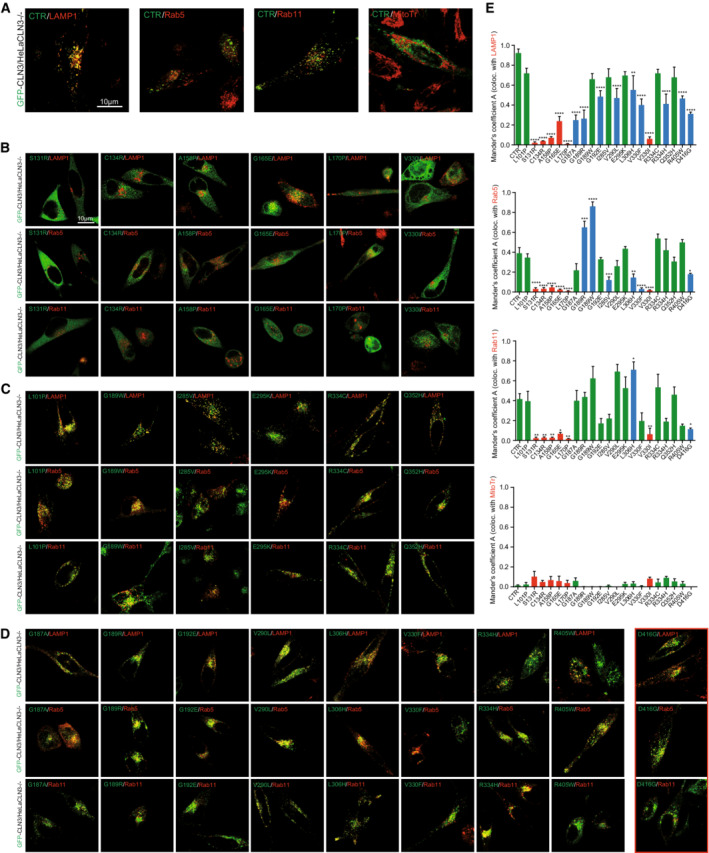
Colocalization of GFP‐CLN3 and Batten disease‐causing missense mutants with endolysosomal markers (LAMP1 for LE/LY, Rab5 for EE, and Rab11 for RE) and MitoTracker‐DR A–DConfocal images of CLN3 KO HeLa cells cotransfected with either GFP‐CLN3 CTR (WT) or GFP‐CLN3 missense mutant variants (as indicated) and endolysosomal markers: LAMP1‐RFP, Rab5‐RFP, or Rab11‐DsRed. Six mutants (B), CLN3^S131R^, CLN3^C134R^, CLN3^A158P^, CLN3^G165E^, CLN3^L170P^, and CLN3^V330I^, appeared strongly mislocalized to the cytosol. When present in patients (usually heterozygously, alongside the more prevalent CLN3^Δ1.02kb^ variant), these variants reportedly result in a variety of clinical phenotypes, including classic JNCL, cone‐rod dystrophy, autophagic vacuolar myopathy, or retinitis pigmentosa (RP). A further six mutants (C), CLN3^L101P^, CLN3^G189W^, CLN3^I285V^, CLN3^E295K^, CLN3^R334C^, and CLN3^Q352H^ showed no significant difference in colocalization with LAMP1, Rab5, or Rab11 compared to CTR CLN3. For these, clinical phenotypes have not been described, incompletely characterized, or described as protracted Batten disease or RP. The remaining nine mutations (D), CLN3^G187A^, CLN3^G189R^, CLN3^G192E^, CLN3^V290L^, CLN3^L306H^, CLN3^V330F^, CLN3^R334H^, CLN3^R405W^, and CLN3^D416G^ showed significantly reduced lysosomal localization (LAMP1), while retaining endosomal localization. CLN3^D416G^ also showed a significant decrease in Rab11 (recycling endosome) colocalization compared to CLN3 CTR. Rab5 (early endosome) colocalization was altered in four of these nine mutants, including CLN3^D416G^. Due to its consistent reduction in colocalization with all endolysosomal markers, CLN3^D416G^ was chosen as a candidate for iPSC generation (with classic, more severe clinical JNCL phenotype).EQuantification of experiments as shown in A‐D. Shown are the respective Mander's correlation coefficients (MCC) for automated colocalization analysis (JACoP/Fiji) of GFP‐CLN3 CTR and missense mutants with LAMP1‐RFP, Rab5‐RFP, Rab11‐DsRed, or MitoTracker‐DR (negative control). Data information: Data are presented as mean ± SD. *n* > 3 technical and biological replicates for each tested condition; one‐way ANOVA Dunnett's multiple comparisons test. *p‐value < 0.1; **p‐value < 0.01; ***p‐value < 0.001; ****p‐value < 0.0001.

**Figure EV4:**
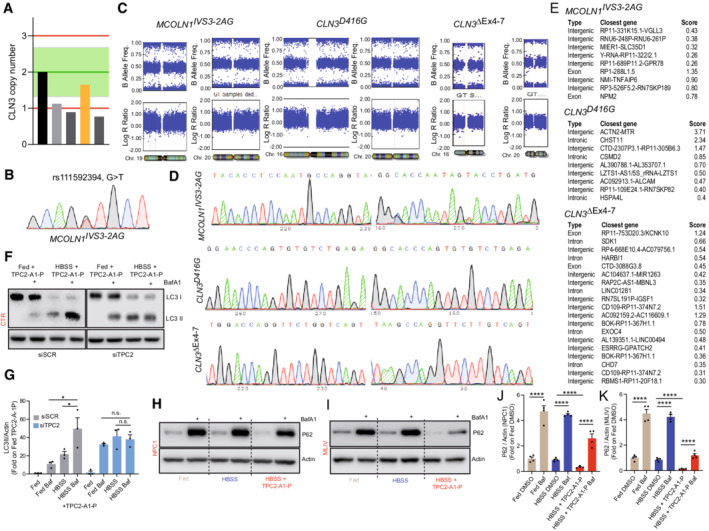
Quality control of novel MLIV and CLN3 iPSC lines and autophagy (LC3 and P62) experiments AFour CLN3^D416G^ iPSC clones were screened for locus copy numbers by qgPCR in comparison to the unedited parent line (black bar) to rule out undesired on‐target editing. The clone showing two CLN3 copies was selected (yellow bar).BA heterozygous, silent SNP was found alongside the MLIV^IVS3‐2A>G^ edit, confirming the presence of both edited alleles and ruling out large indels due to on‐target effects.CMolecular karyotyping did not reveal any detectable aberrations in the selected cell lines at the targeted locus or chromosome 20, which is frequently altered in edited iPSCs.DThe most likely off‐target sites of the gRNAs used for each edit were predicted by CFD and MIT algorithms and sequenced, revealing no off‐target editing in the selected CLN3 and MLIV clones. The most likely off‐target sites for each clone are depicted.ETabular summary of sequenced off‐target sites.F, GEffect of HBSS + TPC2‐A1‐P on LC3 in siSCR or siTPC2‐‐treated human CTR fibroblasts.H–KEffect of TPC2‐A1‐P on P62 accumulation with and without bafilomycin A1 treatment in human NPC1 and MLIV fibroblasts. Data information: Shown are mean values ± SEM. *n* > 3 technical and biological replicates for each tested condition (each dot in (F), (H), and (I) represent three independent western blot experiments, respectively); two‐tailed Student's *t*‐test (G, J, K). *p < 0.05; ****p‐value < 0.0001.

To extrapolate our patient fibroblast data to human neurons with isogenic controls, we used CRISPR/Cas9 to generate iPSCs expressing either the most common MLIV‐causing mutation MCOLN1^IVS3‐2A>G^ (Bargal *et al*, 2001) or the JNCL‐causing mutation CLN3^D416G^. In addition, we generated a knockout model for CLN3 (CLN3^ΔEx4–7^) (Fig 4A and B). To identify a suitable JNCL point mutant candidate, we performed a systematic analysis of the subcellular localization of disease‐causing CLN3 point mutations and correlated them with reported clinical phenotypes (Fig EV3A–E). Based on this analysis, we chose CLN3^D416G^, which shows significant reduction in endolysosomal localization compared to its WT counterpart but not complete mislocalization. Clinically, CLN3^D416G^ causes the classical, severe JNCL phenotype, marked by retinitis pigmentosa and progressive neurodegeneration (Kousi *et al*, 2012). The mutations were engineered into WT A18944 iPSCs (CTR) using CRISPR/Cas9‐mediated gene editing (Fig 4A and B) (Weisheit *et al*, 2020). Active gRNAs (Brinkman *et al*, 2014) were transfected alongside spCas9 and repair template. Since the cut sites overlapped with introduced mutations, our approaches did not require blocking mutations to prevent re‐editing, yielding several homozygously edited clones (Paquet *et al*, 2016; Kwart *et al*, 2017). Established iPSC clones were deeply quality controlled to exclude undesired on‐target effects by qgPCR and SNP genotyping (Weisheit *et al*, 2020, 2021), integration of editing components by confirming puromycin sensitivity, chromosomal abnormalities by performing molecular karyotyping, and off‐target effects by sequencing the top off‐target sites determined by two distinct algorithms (CFD/MIT) (Fig EV4A–E). Maintenance of pluripotency in edited lines was confirmed by staining for pluripotency markers Tra1‐60, Oct4, SSEA4, and NANOG (Fig 4C).

### Effect of TPC2 activation in neurons derived from human LSD iPSCs

**Figure 5:**
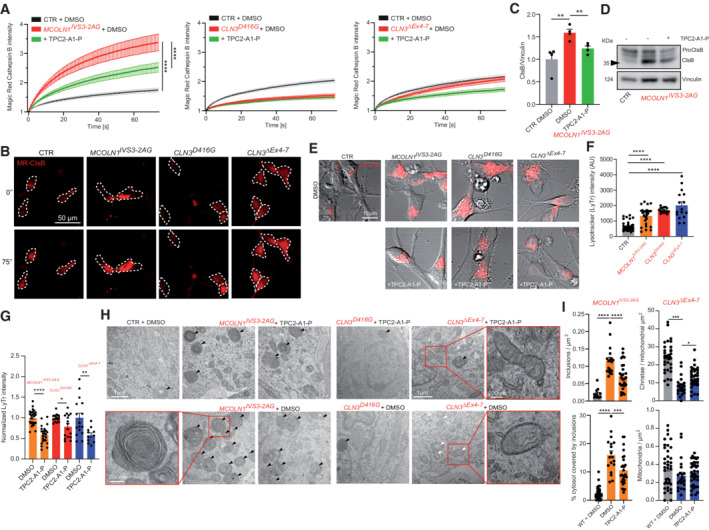
Effect of TPC2‐A1‐P on human neuronal LSD phenotypes Cortical neurons were differentiated from iPSCs, generating lysosomal storage disease neurons and isogenic controls.
A, BLysosomal proteolysis was measured following pre‐treatment with DMSO/TPC2‐A1‐P, using the magic red (MR) cathepsin B substrate, and performing FRAP measurements to assess the proteolysis rate. MCOLN1^IVS3‐2A>G^ (MLIV) neurons showed increased proteolysis, while CLN3^D416G^ and CLN3^ΔEx4–7^ neurons (JNCL) exhibited either significantly lower or slightly reduced proteolysis rates, respectively.C, DWestern blot analysis of cathepsin B (CtsB) in CTR and MCOLN1^IVS3‐2A>G^ neurons treated with TPC2‐A1‐P (30 μM) or DMSO.E–GCortical neurons were treated with compounds and acidic compartments stained with LysoTracker (LyTr). The endolysosomal expansion was observed in MCOLN1^IVS3‐2A>G^, CLN3^D416G,^ and CLN3^ΔEx4–7^ neurons, which was ameliorated by TPC2‐A1‐P (30 μM) treatment.H, IElectron microscopy analysis of neuronal rosettes (neuronal progenitor cells, NPC) treated with DMSO or TPC2‐A1‐P. TPC2‐A1‐P treatment significantly decreased the number of inclusion bodies (black arrowheads) in MCOLN1^IVS3‐2A>G^. CLN3^D416G^ and CLN3^ΔEx4–7^ lacked an appropriate assay window and showed no significant accumulation of inclusion bodies. However, CLN3^ΔEx4–7^ NPC showed significantly more mitochondria with aberrant cristae numbers (white arrowheads), a phenotype which was rescued by TPC2‐A1‐P (30 μM) treatment. Data information: Shown are mean values ± SEM. *n* > 3 technical and biological replicates for each tested condition (each dot represents an imaged frame containing several cells, obtained from at least three distinct neuronal differentiations); one‐way ANOVA, *post hoc* Tukey's multiple comparisons test, or two‐tailed Student's *t*‐test (C). **p‐value < 0.01; ***p‐value < 0.001; ****p‐value < 0.0001.

JNCL and MLIV are both marked by primary neuronal dysfunction as evidenced by neuronal monocultures developing pathological characteristics such as autophagic defects, ultrastructural abnormalities, and expansion of the lysosomal compartment (Curcio‐Morelli *et al*, 2010; Lojewski *et al*, 2014; Kinarivala *et al*, 2020). We therefore employed our established protocol to differentiate iPSCs into cortical neurons (Paquet *et al*, 2016) (Fig 4D) and assessed whether these neurons express genes relevant for disease (TRPML1 for MLIV and CLN3 for JNCL) and treatment (TRPML1 and TPC2). Transcripts of TRPML1, TPC2, and CLN3 were readily detectable in the cortical neurons, while the endolysosomal cation channels TRPML2, TRPML3, and TPC1 were largely undetectable (Fig 4E). Measuring TRPML1‐dependent currents using the endolysosomal patch‐clamp technique (Chen *et al*, 2017), showed absence and presence in MCOLN1^IVS3‐2A>G^ mutant and CTR neurons, respectively (Fig 4F and G). Phenotypically, we assessed these neurons by analyzing lysosomal cathepsin B (CtsB) activity, LysoTracker (LyTr) staining, and ultrastructures by electron microscopy. To exclude any potential toxic effects of TPC2‐A1‐P on iPSC‐derived neurons again, cell viability assays were performed (Fig EV1C). Increased CtsB activity is linked to cell death in MLIV (Colletti *et al*, 2012) and, conversely, decreased CtsB activity has been reported in CLN3 disease (Metcalf *et al*, 2008). We applied fluorescence recovery after photobleaching (FRAP) as established by Metcalf *et al* (2008), finding MCOLN1^IVS3‐2A>G^ neurons to exhibit significantly increased CtsB activity, while JNCL (CLN3^D416G^ and CLN3^ΔEx4–7^) neurons either exhibited slightly reduced or unchanged CtsB activity compared to CTR. TPC2‐A1‐P treatment significantly decreased CtsB activity in iPSC‐derived MCOLN1^IVS3‐2A>G^ neurons (Fig 5A and B). We next assessed the protein levels of intracellular CtsB by western blot analysis, finding increased CtsB levels in MCOLN1^IVS3‐2A>G^ compared to CTR neurons, rescued by TPC2‐A1‐P treatment (Fig 5C and D). We further assessed acidic compartments by LyTr (LysoTracker) staining. The lysosomal compartment appeared expanded in MCOLN1^IVS3‐2A>G^ and JNCL neurons compared to CTR, which was ameliorated upon TPC2‐A1‐P treatment (Fig 5E–G). We continued with electron microscopy analyses of MCOLN1^IVS3‐2A>G^ and JNCL neuronal progenitor cells to assess their ultrastructure. Lysosomal inclusion bodies were readily detected in DMSO‐treated MCOLN1^IVS3‐2A>G^ neuronal progenitor cells (NPC), and their number was significantly decreased upon TPC2‐A1‐P treatment. Ultrastructural analyses in JNCL cells on the other hand revealed no significant change in inclusion body density, remaining further unchanged upon TPC2 activation or DMSO treatment (Fig 5H and I). However, the Cristae numbers per mitochondrial area were significantly reduced in CLN3^ΔEx4–7^ compared to CTR NPC, and TPC2‐A1‐P treatment significantly increased these numbers again (Fig 5H and I).

### Lysosomal exocytosis and autophagy as potential rescue mechanisms

**Figure 6:**
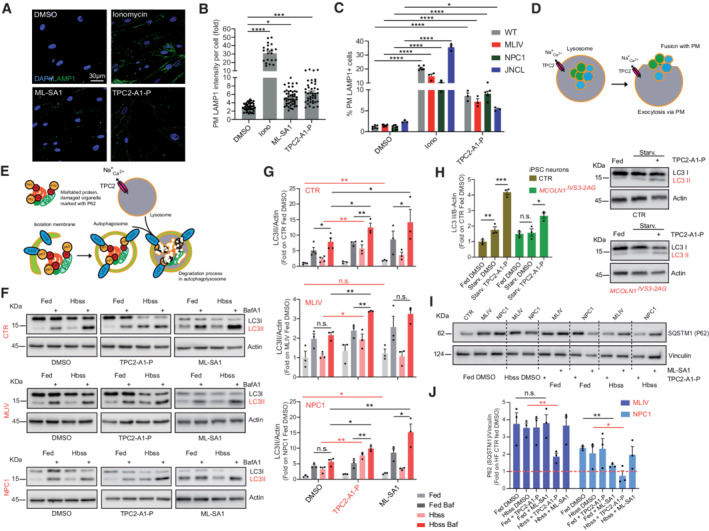
Effect of TPC2‐A1‐P on lysosomal exocytosis and autophagy AConfocal images of plasma membrane (PM) LAMP1 immunofluorescence in CTR fibroblasts. LAMP1 on the PM is expressed as fold change relative to DMSO‐treated cells.BStatistical analysis of lysosomal exocytosis data as shown in (A).CLysosomal exocytosis in CTR, MLIV, NPC1, and JNCL human fibroblasts. PM‐localized LAMP1 was measured by flow cytometry, expressed as percent of CTR DMSO‐treated cells. Ionomycin in (A–C) (4 μM; 10 min treatment) was used as a positive control. TPC2‐A1‐P and ML‐SA1 (30 μM, each; 90 min treatment in A–C).DCartoon showing lysosomal exocytosis. Statistics (B, C): Shown are mean values ± SEM. *n* > 3 for each tested condition (in (B), each dot represents an imaged frame containing several cells, and in (C) each dot is the mean FITC intensity value expressed as a percentage obtained from at least 1 × 10^4^ events); two‐way ANOVA, *post hoc* Dunnett's (B), or Tukey's (C) multiple comparisons test; *p‐value < 0.05; ***p‐value < 0.001; ****p‐value < 0.0001.ECartoon showing the roles of LC3 and P62 in the autophagic pathway.F, GImmunoblot analysis of endogenous LC3 (LC3I‐II) following TPC2‐A1‐P or ML‐SA1 (30 μM, each) treatment, alone or with BafA1, under fed (complete media), or starvation (HBSS) conditions in CTR, MLIV, and NPC1 patient fibroblasts. Graphs show densitometry of LC3II bands normalized to actin.HImmunoblot analysis of endogenous LC3 (LC3I‐II) following TPC2‐A1‐P (30 μM) or DMSO treatment, under fed (complete neurobasal/B27), or starvation (DMEM/F12 free) conditions in CTR and MLIV iPSC‐derived cortical neurons. Graphs show densitometry of LC3II bands normalized to actin.I, JImmunoblot and statistical analysis of endogenous SQSTM1 (P62) upon TPC2‐A1‐P or ML‐SA1 (30 μM, each) treatment, under fed (complete media), or starvation (HBSS) conditions in CTR, MLIV, and NPC1 patient fibroblasts. Data information: In (G, H, J) shown are mean values ± SD. *n* = 3 lysates per condition pooled from three independent experiments; two‐tailed Student's *t*‐test. *p‐value < 0.05; **p‐value < 0.01; ***p‐value < 0.001.

We next examined the effect of TPC2‐A1‐P on lysosomal exocytosis in LSD cells as potential mechanism, underlying the observed rescue effects. Using LAMP1 translocation to the plasma membrane as readout, we found that TPC2‐A1‐P has a similar effect on lysosomal exocytosis in CTR as well as in MLIV, NPC1, and JNCL patient fibroblasts, demonstrating an intact TPC2‐mediated exocytosis capability in the diseased cells (Fig 6A–D). As positive controls, the TRPML1 agonist ML‐SA1 and ionomycin were used. We next assessed the effect of TPC2‐A1‐P on autophagy. Again, as positive control, ML‐SA1 was used. TPC2‐A1‐P increased starvation‐mediated autophagy in CTR fibroblasts (Fig 6E–G) in a TPC2‐dependent manner as demonstrated by siRNA experiments (Fig EV4F and G) and recovered impaired autophagic flux in NPC1 and MLIV fibroblasts (Fig 6F and G). Likewise, in iPSC‐derived cortical neurons, TPC2‐A1‐P increased starvation‐mediated autophagy in CTR and MCOLN1^IVS3‐2A>G^ (MLIV) neurons (Fig 6H). The autophagic flux blockade in NPC1 and MLIV fibroblasts also leads to P62/Sequestosome 1 (SQSTM1) accumulation (Vergarajauregui *et al*, 2008; Elrick *et al*, 2012; Sarkar *et al*, 2013). While starvation alone does not sufficiently clear P62 accumulation, we found that treatment with TPC2‐A1‐P under starvation conditions alleviates the autophagic flux blockade in MLIV and NPC1 fibroblasts, clearing the accumulated P62 (Figs 6I and J, and EV4H–K).

### TPC2 expression in brain assessed by RT‐qPCR and by analyzing a novel reporter mouse model

**Figure 7:**
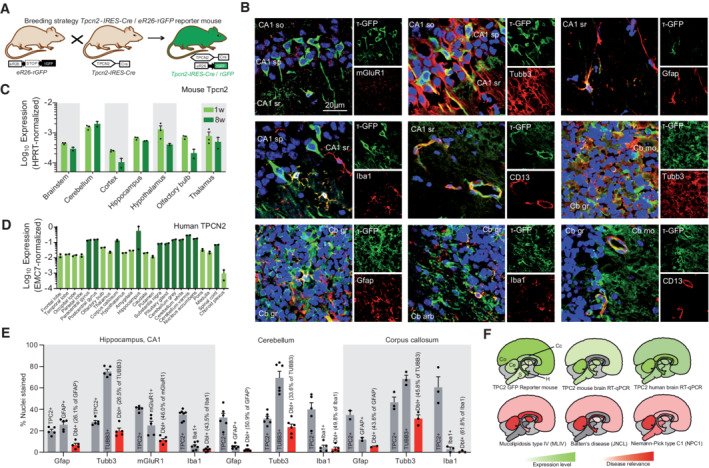
Expression of TPC2 in human and mouse brains The TPC2 reporter mouse *Tpcn2* ^IRES‐Cre/eR26‐τGFP^ was generated as previously described (Wen *et al*, 2011; Wyatt *et al*, 2017).
A–BBoth neurons and glia were found to express Tpc2 in the corpus callosum, the hippocampus, and the cerebellum (so, stratum oriens; sp, stratum pyramidale; sr, stratum radiatum; mo, molecular layer; gr, granular layer). Subpopulations of astrocytes (Gfap) and microglia (Iba1) express Tpc2 (E).COne week (1w)‐ or 8‐week (8w)‐old mouse brains were dissected, and brain Tpc2 transcript was mapped.DA cDNA array was used to map TPC2 transcripts in the human brain.EQuantification of astrocytes(Gfap), microglia (Iba1) and neurons in different brain areas and percentage of cells coexpressing Tpc2 and the respective marker (Dbl = double labeled).FTpc2 expression in the Tpc2 reporter mouse, mouse brain, and human brain is summarized as cartoons, finding highest expression in cerebellum and hippocampus (top panels). Affected brain regions in the lysosomal storage diseases MLIV, JNCL, and NPC1 based on patient and mouse data are color-coded.

**Figure EV5:**
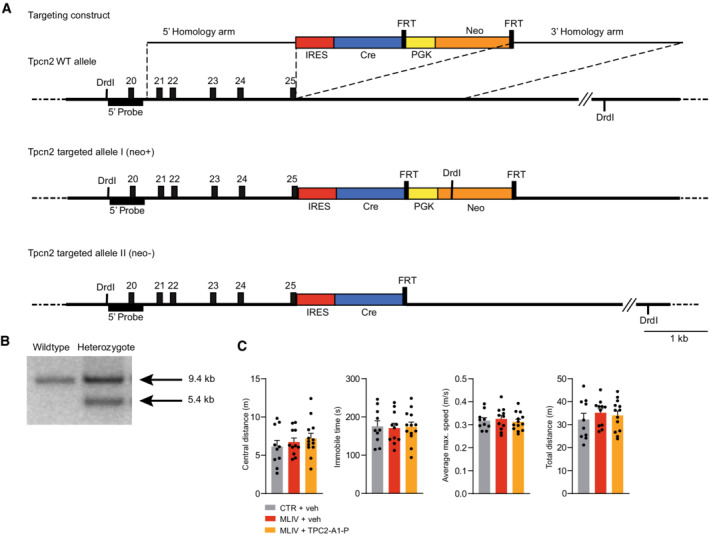
Targeting strategy for TPC2 reporter mouse model and supplementary behavioral data (MLIV mouse model) ATargeting strategy used to express Cre recombinase under the control of the Tpcn2 promoter. The targeting vector contains an IRES‐Cre‐FRT‐PGK‐NEO‐FRT cassette in which a phosphoglycerate kinase promoter drives neomycin resistance (Pgk‐neo). This cassette is incorporated by homologous recombination in embryonic stem cells subsequent to the stop codon in exon 25.BSouthern blot of embryonic stem cell DNA cut with DrdI, demonstrating correct targeting of the Tpcn2‐IRES‐Cre knock‐in allele.CResults of the open‐field test using MLIV mice treated with vehicle or TPC2‐A1‐P, respectively, compared to vehicle‐treated WT littermates (CTR). No differences between CTR and MLIV mice were observed.

To investigate the *in vivo* efficacy of TPC2‐A1‐P, we made use of the MLIV mouse model (Venugopal *et al*, 2007; Grishchuk *et al*, 2014, 2015; Walker & Montell, 2016). One essential prerequisite for TPC2 as a drug target for neurodegenerative LSDs is expression in various cell types of the CNS. To assess Tpc2 expression in the brain, we generated a TPC2 reporter mouse model (*Tpcn2* ^IRES‐Cre/eR26‐τGFP^) (Figs 7A and EV5A and B) (Wyatt *et al*, 2017). The labeling of TPC2‐positive cells via expression of τGFP is dependent on the expression of Cre recombinase under control of the TPC2 promotor. Focusing on the hippocampus and cerebellum, two vulnerable brain regions in LSD‐associated neurodegeneration (Frei *et al*, 1998; Prasad *et al*, 2000; Greene *et al*, 2001; Pontikis *et al*, 2004; Walkley & Suzuki, 2004; Grishchuk *et al*, 2014, 2015), we observed the most distinct Tpc2 expression pattern in neuronal fibers extending toward the hippocampal CA3 pyramidal layer. Furthermore, throughout the hippocampus, Tpc2^+^ pyramidal neurons and processes were readily observed (Fig 7B). Tpc2 was also expressed in hippocampal and cerebellar astrocytes, microglia, and mural cells (CD13^+^) (Fig 7B). To quantify channel expression, we analyzed Tpc2 transcript levels in the mouse brain, finding Tpc2 transcripts in cortex, hippocampus, cerebellum, and other brain regions (Fig 7C). We also assessed TPC2 transcription in the human brain (Fig 7D). The highest TPC2 expression was observed in hippocampus, cerebellum, corpus callosum, nucleus accumbens, and paracentral and postcentral gyrus (Fig 7D). We further quantified cell‐type‐specific expression in hippocampus, cerebellum, and corpus callosum using the reporter mouse model (Fig 7E). In conclusion, TPC2 is expressed in all relevant cell types and regions of the brain to treat the lysosomal storage diseases under investigation here (Fig 7F).

**Figure 8:**
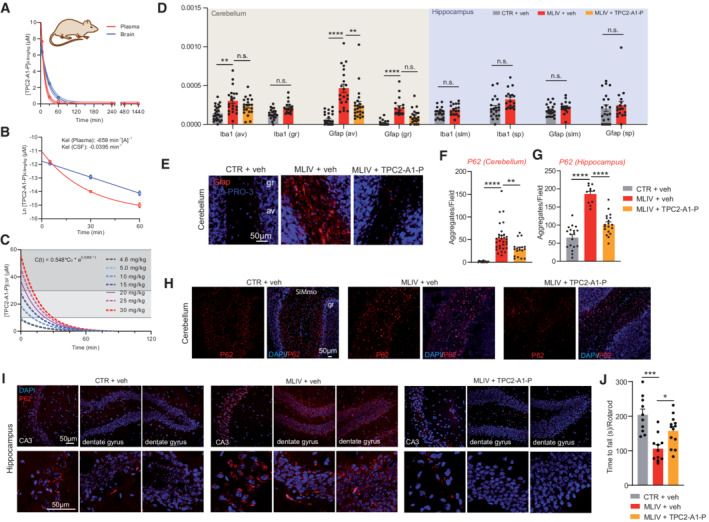
TPC2‐A1‐P pharmacokinetics and *in vivo* rescue effects in MLIV mice ATPC2‐A1‐P was injected intravenously, and mice were sacrificed at the indicated time. TPC2‐A1‐P was measured in plasma and brain by LC–MS/MS. TPC2‐A1‐P was rapidly eliminated, being undetectable by 240 min.BElimination rate constants were determined using a semi‐log plot. A two‐phase decay model could fit the obtained data points in plasma, while a one‐phase decay model fits the data in the brain.CBrain [TPC2‐A1‐P] was simulated for various injected doses. 20 mg/kg TPC2‐A1‐P was chosen to avoid off‐target activity while providing a therapeutic dose for > 20 min.D, EFrom 2 months of age, MLIV mice were injected daily with TPC2‐A1‐P i.p. and sacrificed after 13 weeks; av, arbor vitae; gr, granular cell layer; slm, stratum lacunosum moleculare; and sp, stratum pyramidale. Mild microgliosis (Iba1) was observed only in the cerebellar av, while astrogliosis (Gfap) was observed in the cerebellar av and gr layers in MLIV mice. TPC2‐A1‐P ameliorated MLIV‐associated cerebellar astrogliosis.F, GPlots showing mean numbers of P62 aggregates per section (cerebellum (F) and hippocampus (G)).H, IConfocal images of endogenous P62/SQSTM1 inclusion in CTR and MLIV mouse cerebellar coronal (H) and hippocampal (I) sections.JResults of the rotarod experiments using MLIV mice treated with vehicle or TPC2‐A1‐P, respectively, compared to vehicle‐treated WT littermates (CTR). Data information: Shown in (D) are mean cell densities for the indicated marker ± SEM; shown in (F, G) and (J) are mean values ± SEM (each dot represents an imaged frame containing several cells, >3 frames per condition (F, G) or single animals (J)); two‐way ANOVA, *post hoc* Bonferroni's (D), Dunnett's (F, G), or Tukey's (J) multiple comparisons test. *p < 0.05; **p < 0.01; ***p‐value < 0.001; ****p < 0.0001. The following mouse numbers per condition were used: CTR+DMSO, *n* = 6; MLIV + DMSO, *n* = 3; MLIV + TPC2‐A1‐P, *n* = 4 (D–I); CTR+veh, *n* = 10; MLIV + veh, *n* = 11; MLIV + TPC2‐A1‐P, *n* = 13 (J).

### Pharmacokinetics and *in vivo* testing of TPC2‐A1‐P in the MLIV mouse model

To determine blood–brain barrier permeability and clearance of TPC2‐A1‐P, we injected CTR (WT) mice with the compound and measured its levels in plasma and brain by LC–MS/MS. Following its injection, TPC2‐A1‐P decayed rapidly both in plasma and brain, being undetectable after 240 min (Fig 8A). We determined the elimination rate constants in the plasma and brain, fitting a two‐phase decay model to plasma elimination, and a one‐phase decay model in the brain (Fig 8B). We extrapolated these results to predict that an injection of 20 mg/kg TPC2‐A1‐P would yield a therapeutic dose for ca. 20 min upon injection (estimated C_0_ between 30 and 60 μM and above 10 μM for ca. 20 min; Fig 8C). Due to its rapid clearance, we opted for a daily intraperitoneal treatment regimen. After 3 months of daily injections, mice were sacrificed, and brains were collected for histology. Previous reports demonstrated gliosis in both human patients (Folkerth *et al*, 1995) and the MLIV mouse model (Grishchuk *et al*, 2014; DeRosa *et al*, 2021). We assessed gliosis in cerebellum and hippocampus of the MLIV mouse model, observing prominent astrogliosis in the cerebellar arbor vitae (av) and granular (gr) cell layer, and mild microgliosis of the cerebellar arbor vitae, while no significant differences were seen in hippocampus (Fig 8D). Mice injected with TPC2‐A1‐P were found to show significant amelioration of the astrogliosis phenotype in the cerebellar av (Fig 8D and E). Furthermore, P62/SQSTM1 aggregates were shown previously to accumulate in the central nervous system of the MLIV mouse model, suggesting an impairment in protein degradation (Micsenyi *et al*, 2009). Indeed, we observed a massive accumulation of P62/SQSTM1 inclusions in the MLIV mouse cerebellar granular cell layer and in the hippocampus compared to CTR (WT). Treatment with TPC2‐A1‐P significantly reduced the number of P62/SQSTM1 aggregates (Fig 8F–I). Finally, we tested TPC2‐A1‐P‐ versus vehicle‐treated mice on motor performance on the accelerating rotarod tasks (Walker & Montell, 2016), demonstrating a significant rescue effect of TPC2‐A1‐P over vehicle treatment in MLIV mice (Fig 8J). In contrast to rotarod, no significant differences between CTR and MLIV mice were found in horizontal exploratory activity in the open‐field test (Fig EV5C). Altogether, these data suggest that TPC2 activation is able to restore central nervous system defects and the decline in motor performance in the MLIV mouse model.

## Discussion

Boosting lysosomal trafficking, autophagy, and exocytosis shows a promising therapeutic strategy to improve lysosomal function in several diseases (Medina *et al*, 2011; Bae *et al*, 2014; Grimm *et al*, 2014; Medina *et al*, 2015; García‐Rúa *et al*, 2016; Zhong *et al*, 2016; Grimm *et al*, 2017; Bonam *et al*, 2019; Tsunemi *et al*, 2019). Here, we assessed the effect of TPC2 activation on LSD phenotypes in human fibroblasts and isogenic iPSC‐based neuronal models. We show that TPC2 activation with TPC2‐A1‐P rescues storage phenotypes in MLIV, NPC1, and JNCL cells. Our *in vivo* results further indicate that TPC2‐A1‐P restores central nervous system defects, including astrogliosis and accumulation of P62/SQSTM1 inclusions in MLIV mice (Folkerth *et al*, 1995; Grishchuk *et al*, 2014; DeRosa *et al*, 2021), as well as improves their motor performance (rotarod). Endolysosomes depend on the activity of their channels and transporters, dysfunction of which often severely affects organelle function and underlies neurodegenerative disease pathology. TRPML1 and TPC2 are the primary lysosomal Ca^2+^ release channels, mediating the Ca^2+^ efflux that so often is impaired in neurodegeneration (e.g., NPC1, MLIV, Fabry, and Alzheimer's disease (Feng & Yang, 2016)). TRPML1 function is directly affected in MLIV (Chen *et al*, 2014), while in NPC1 and other LSDs, TRPML1 activity is reduced, e.g., by accumulating sphingomyelin (Shen *et al*, 2012). In addition, lysosomal pH is often increased in LSDs, affecting the activity of not only many lysosomal enzymes but also TRPML1 (Dong *et al*, 2010). While TRPML1 activity is pH dependent, decreasing with increasing pH, TPC2 activation by PI(3,5)P_2_ is pH independent (Wang *et al*, 2012), which may be an advantage when targeting TPC2. Our results provide an incentive to further investigate the potential benefit of TPC2 activation in various LSDs. Besides LSDs, TRPML1/TPC2 activation may also have relevance for adult‐onset neurodegenerative disease therapy. Indeed, stimulation of lysosomal exocytosis via TRPML1 has recently been shown to clear α‐synuclein accumulation in Parkinson's disease dopaminergic neurons (Tsunemi *et al*, 2019), while another study demonstrated that activation of TRPML1 cleared amyloid‐beta (Aβ), which accumulates by unknown mechanisms in the lysosomal and autophagic compartments of neurons in the HIV‐infected brain (Bae *et al*, 2014). These examples encourage further investigation of targeting TRPML1/TPC2 also in adult‐onset neurodegenerative disease therapy.

## Materials and Methods

### Human fibroblast cell culture and electroporation

The following human fibroblast cells isolated from healthy/diseased individuals were investigated: CTR (control) (GM00969), MLIV (GM02048/MCOLN1^IVS3‐2A>G/Ex1‐7del^), NPC1 (NPC1^P237S/I1061T^; GM03123), NPA (SMPD1^L302P/L302P^; GM00112), Gangliosidosis (GLB1^R201C/R201C^; GM02439), Gaucher (GBA^N370S/V394L^; GM01607), and Fabry (GLA^W162+IVS4‐16A>G+IVS6‐22C>T^; GM00107) from Coriell, and JNCL fibroblasts (CLN3^Δ1.02kb/Δ1.02kb^; MIN30068). The cells were grown in DMEM (supplemented with 1 g/l glucose, pyruvate, GlutaMAX, 15% FBS, and 1% P/S) and kept at 37°C with 5% CO_2_. Cells were electroporated using the Neon system (Invitrogen) with 100 μl tips according to the manufacturer's instructions, electroporating 10^6^ cells at a time with 5 μg plasmid using 2 × 20 ms 1,400 V pulses. Following electroporation, 30,000 cells were seeded for cell biological assays into ibiTreat‐coated eight‐well chambers (ibidi) or onto poly‐L‐lysine‐coated 12 mm glass coverslips.

### Lactosylceramide (LacCer) trafficking assay

Human fibroblasts were cultured in ibiTreat eight‐well chambers (ibidi) for live‐cell imaging overnight prior to treatments. Cells were treated with 30 μM agonist in DMSO (to a final DMSO concentration of 0,3%) overnight or up to 48 h, and the lactosylceramide trafficking assay was subsequently initiated: Cells were washed once with PBS, and 25 μM LacCer (BODIPY FL C5‐Lactosylceramide, Invitrogen) pulsed in serum‐free culture medium for 1 h at 37°C. Cells were washed twice with PBS and chased with complete DMEM (including 15% FBS and the indicated agonists) for 2 h at 37°C. LyTr‐DR (LysoTracker‐Deep Red; diluted 1:10,000, Invitrogen) was added 1,5 h into the chase time to visualize acidic organelles. The cells were subsequently washed three times with PBS, before adding a complete phenol‐red‐free medium for imaging. The cells were transferred to a pre‐heated 37°C incubation chamber mounted onto a Zeiss Confocal microscope (LSM 880) and imaged using a 63 X water objective at 488 nm (LacCer) and 633 nm (LyTr) excitation wavelength, respectively. For data quantification, the Fiji software was used alongside the JACoP plugin for colocalization quantification, calculating the Mander's coefficient for LyTr‐DR overlapping LacCer. LacCer density calculations were performed using Harmony High‐Content Imaging and Analysis Software (PerkinElmer).

### Filipin unesterified cholesterol storage assay

Human fibroblasts were cultured in 24‐well chambers on poly‐L‐lysine‐coated coverslips overnight prior to treatments. Cells were treated with 30 μM agonist in DMSO (to a final DMSO concentration of 0.3%) for 48 h, and the filipin staining was initiated: Cells were washed twice with ice‐cold PBS, and fixed in 4% PFA for 30 min. Fixed cells were again washed with cold PBS, and unesterified cholesterol was visualized by filipin staining (PBS with 0.05 mg/mL filipin, Sigma‐Aldrich, and 10% FBS) for 2 h at room temperature in a dark humid chamber. Cells were subsequently washed with ice‐cold PBS twice, and nuclei stained using TO‐PRO‐3 (1:500, Invitrogen). Cells were washed twice and mounted on microscope slides overnight for imaging. Images were captured using a Zeiss Confocal Microscope (LSM 880), using a 40X oil objective, at 405 nm (filipin), 560 nm (mCherry), and 633 nm (TO‐PRO‐3). For data quantification, we calculated average filipin intensity per cell using Harmony High‐Content Imaging and Analysis Software (PerkinElmer).

### Mitomycin C treatment and JNCL autofluorescence analysis

Human fibroblasts (CTR and JNCL) were treated for 2 h with 30 μM mitomycin C (Millipore) to induce cell cycle arrest. Cells were seeded onto a glass coverslip (2,5 x 10^4^) overnight. After 16 h, t0 cells were fixed with PFA 4% or treated for 72 h with DMSO, TPC2‐A1‐P, or ML‐SA1 (30 μM). After 72 h, cells were fixed with 4% PFA. PFA was quenched for 10 min with 50 mM NaCl in DPBS 1X. Cells were then blocked and permeabilized in blocking buffer (0.05% Saponin, 1%BSA, and 50 mM NaCl) for 20 min. LAMP1 antibody exposure was performed overnight (1:800, SantaCruz). Cells were then incubated with Alexa Fluor 594‐conjugated secondary antibody (Thermo Fisher) for 1 h at room temperature. Nuclei were stained using To‐Pro (Thermo Fisher, 1:500 in PBS 1X) for 20 min. Confocal images were acquired using an LSM 880 microscope (Zeiss) with 40X magnification. Autofluorence mean intensities at 488 nm and 405 nm excitation in the LAMP1+ area were calculated using unsaturated images on ImageJ 1.52a software.

### Lysosomal exocytosis experiments and isolation and culture of primary macrophages

Lysosomal exocytosis experiments were performed as described previously (Gerndt *et al*, 2020). Further details are provided in the Appendix Supplementary Methods.

### Autophagy assays

Human CTR, MLIV, and NPC1 fibroblasts (5 × 10^4^) were seeded in 12‐well plate overnight. Treatment was performed for 180 min in complete media or HBSS 10 mM Hepes (Thermo Fisher) with DMSO or TPC2‐A1‐P (30 μM) or ML‐SA1 (30 μM). To determine the amplitude of the autophagic flux, a cotreatment with 100 nM of the vacuolar ATPase inhibitor Bafilomycin A1 (Millipore) was performed. Samples were then prepared for western blot analysis. For western blot analysis, antibodies were used as indicated in the Appendix Supplementary Methods.

### Site‐directed mutagenesis and colocalization analysis using confocal microscopy

All human CLN3 mutants were generated from WT cDNA templates using QuikChange Site‐Directed Mutagenesis Kit (Stratagene), following manufacturer's instructions. Further details are provided in the Appendix Supplementary Methods.

### Generation and quality control of lysosomal storage disease iPS cells

The protocol for generating homozygous knock‐in mutations in induced pluripotent stem cells (iPSCs) has previously been extensively described (Paquet *et al*, 2016). All details are provided in the Appendix Supplementary Methods.

### Differentiation and staining of lysosomal storage disease iPSC‐derived cortical neurons and staining

Cortical neurons were obtained as previously described (Paquet *et al*, 2016). All details are provided in the Appendix Supplementary Methods.

### Real‐time quantitative PCR analysis

In order to assess the expression levels of the target channels and disease genes, we used real‐time quantitative PCR (RT‐qPCR). Further details are provided in the Appendix Supplementary Methods.

### LysoTracker (LyTr) staining

iPSC‐derived neurons were terminally matured in glass‐bottom, poly‐ornithine/laminin‐coated eight‐well chambers (ibidi) as previously described, using DAPT and 5‐FU for 7 days, and kept in culture for another week before imaging. iPSC‐derived neurons were treated with 0.3% DMSO or 30 μM TPC2‐A1‐P for 48 h prior to live‐cell imaging. LyTr‐DR was added at a dilution factor of 1:10,000 to the culture medium 30 min prior to confocal microscopy. The cells were transferred to a pre‐heated 37°C incubation chamber mounted onto a Zeiss Confocal microscope (LSM 880) and imaged using a 63 X water objective and an excitation wavelength of 633 nm (LyTr). Quantification of captured images was performed using the Fiji software. A mask was generated around the neuronal cell bodies, and the mean intensity was recorded.

### Magic Red Cathepsin B activity measurements

We used fluorescence recovery after photobleaching (FRAP) approach previously utilized for assessing proteolysis upon CLN3 knockdown (Metcalf *et al*, 2008) to assess proteolysis in iPSC‐derived neurons. Further details are provided in the Appendix Supplementary Methods.

### Endolysosomal patch‐clamp experiments

Endolysosomal patch‐clamp experiments were performed as described previously (Chen *et al*, 2017). Further details are provided in the Appendix Supplementary Methods.

### Generation of the TPC2 reporter mouse line

Mice harboring the Tpcn2^IRES‐Cre^ locus were bred with ROSA26‐floxed stop‐τGFP mice, giving rise to mice constitutively expressing τGFP under the control of the TPC2 promoter. Further details are provided in the Appendix Supplementary Methods.

### Pharmacokinetic study of TPC2‐A1‐P in C57Bl/6N mice

The purpose of this study was to determine the pharmacokinetic characteristics of TPC2‐A1‐P in C57Bl/6N mice following single intravenous (IV) dosing. Study design, animal selection, handling, and treatment were all in accordance with the Enamine PK study protocols and conducted by the animal laboratory personnel at Enamine/Bienta. All details of the study are provided in the Appendix Supplementary Methods.

### Electron microscopy experiments

Electron microscopy experiments were performed as recently described (Polishchuk *et al*, 2019). Details are provided in the Appendix Supplementary Methods.

### Cell viability assay

Cell viability assays were performed using CellTiter‐Blue reagent according to the manufacturer's protocol. Further details are provided in the Appendix Supplementary Methods.

### Rotarod and open field

Rotarod and open‐field experiments were performed as recently described (Giordano *et al*, 2018; De Risi *et al*, 2021). Details are provided in the Appendix Supplementary Methods.

### Statistics

Detailed information about statistics is provided in every figure legend.

## Author contributions

**Anna Scotto Rosato:** Data curation; formal analysis; methodology. **Einar K Krogsaeter:** Data curation; formal analysis. **Dawid Jaślan:** Data curation; formal analysis; methodology. **Carla Abrahamian:** Data curation; formal analysis. **Sandro Montefusco:** Formal analysis; investigation. **Chiara Soldati:** Methodology. **Barbara Spix:** Data curation; formal analysis. **Mariateresa Pizzo:** Data curation; investigation. **Giuseppina Grieco:** Formal analysis; methodology. **Julia Böck:** Data curation; formal analysis. **Amanda Wyatt:** Data curation; methodology. **Daniela Wünkhaus:** Methodology. **Marcel Passon:** Data curation. **Marc Stieglitz:** Data curation. **Marco Keller:** Methodology. **Guido Hermey:** Resources. **Sandra Markmann:** Methodology. **Doris Gruber‐Schoffnegger:** Methodology. **Susan Cotman:** Resources. **Ludger Johannes:** Methodology. **Dennis Crusius:** Methodology. **Ulrich Boehm:** Funding acquisition; methodology. **Christian Wahl‐Schott:** Resources; funding acquisition. **Martin Biel:** Resources; funding acquisition. **Franz Bracher:** Conceptualization; resources; supervision; funding acquisition. **Elvira De Leonibus:** Data curation; formal analysis; supervision; investigation. **Elena Polishchuk:** Data curation; formal analysis; methodology. **Diego L Medina:** Funding acquisition. **Dominik Paquet:** Conceptualization; resources; supervision; funding acquisition; methodology; project administration; writing – review and editing. **Christian Grimm:** Conceptualization; resources; supervision; funding acquisition; validation; visualization; methodology; writing – original draft; project administration.

In addition to the CRediT author contributions listed above, the contributions in detail are:

E.K., A.S.R., D.J., S.M., C.A., J.B., C.S., B.S., D.W., M.P., and M.S. collected and analyzed data. A.W. and U.B. designed and generated the TPC2 reporter mouse model (*Tpcn2* ^IRES‐Cre/eR26‐τGFP^). M.K. synthesized and quality‐controlled TPC2‐A1‐P. G.H. provided the HeLa CLN3^−/−^ cells. C.G., C.W‐S., and M.B. provided funding for the generation of the TPC2 reporter mouse. S.C. provided CLN3 patient fibroblasts. S.M. (Evotec) and D.G.S. (Evotec) commented on the manuscript and discussed results. F.B. provided funding and designed chemical syntheses. E.P. and D.M. designed and funded the Shiga toxin (STX) and electron microscopy studies. E.D.L., M.T.P., G.G., and S.M. designed or performed behavioral tests. STX was provided by L.J. D.P. and C.G. designed and funded the study, and collected and analyzed data. C.G. wrote the manuscript. All of the authors discussed the results and commented on the manuscript.

## Disclosure and competing interests statement

The authors declare that they have no conflict of interest.

## For more information

https://www.ncl‐stiftung.de/

http://ml4.org/

https://beyondbatten.org/

https://www.curebatten.org/

https://www.omim.org/entry/252650

https://omim.org/entry/204200

https://www.omim.org/entry/257220

https://lmu‐munich.wixsite.com/lysolabmunich

https://twitter.com/lysolabmunich

https://www.en.wsi.med.uni‐muenchen.de/personen/professors/grimm/index.html

https://www.linkedin.com/in/prof‐dr‐dr‐christian‐grimm‐7097795/

## Supporting information




Appendix S1

Expanded View Figures PDF

## Data Availability

This study includes no data deposited in external repositories.
