# Supplementary material for: TPC2 rescues lysosomal storage in mucolipidosis type IV, Niemann–Pick type C1, and Batten disease
Source: EMBO Mol Med. 2022 Aug 5;14(9):e15377. doi: 10.15252/emmm.202115377 (PMC9449600; doi:10.15252/emmm.202115377)
Supplement: Supplementary file 1 — Appendix S1 [file EMMM-14-e15377-s002.pdf]

Appendix Supplementary Methods

TPC2 rescues lysosomal storage in mucolipidosis type IV, Niemann-Pick type C1 and Batten disease

Anna Scotto Rosato*[1], Einar Krogsaeter*[1], Dawid Jaślan[1], Carla Abrahamian[1], Sandro Montefusco[2], Chiara Soldati[2], Barbara Spix[1], Maria Teresa Pizzo[2], Giuseppina Grieco[2], Julia Böck[1], Amanda Wyatt[3], Daniela Wünkhaus[4], Marcel Passon[1], Marc Stieglitz[5], Marco Keller[5], Guido Hermey[6], Sandra Markmann[4], Doris Gruber-Schoffnegger[4], Susan Cotman[7], Ludger Johannes[8], Dennis Crusius[9], Ulrich Boehm[3], Christian Wahl-Schott[10], Martin Biel[5], Franz Bracher[5], Elvira De Leonibus[2,11], Elena Polishchuk[2], Diego L. Medina[2,12#], Dominik Paquet[9,13#], Christian Grimm[1#]

Suppl. materials and methods

Maintenance of iPSCs

iPSC experiments were performed in accordance with all relevant guidelines and regulations. Female iPSC line A18944 was purchased from ThermoFisher (#A18945). iPSCs were grown in Essential 8 Flex Medium (ThermoFisher, #A2858501) on VTN-coated (ThermoFisher, #A14700) cell culture plates at 37°C with 5% CO_2 and split as small clumps twice a week after a 5 min incubation in PBS/EDTA.

Generation of lysosomal storage disease iPS cells

Design and preparation of editing reagents and quality control of edited iPSCs was performed as described previously (Kwart *et al*, 2017; Weisheit *et al*, 2020, 2021). To generate knock-in patient mutant iPS cells for JNCL (CLN3$^{\text{D416G}}$) and MLIV (MCOLN1$^{\text{IVS3-2A>G}}$), we used CRISPOR (Concordet & Haeussler, 2018) to identify suitable gRNAs mediating efficient and specific homozygous knock-in editing events. gRNAs were cloned into the BsmBI cloning site of the MLM3636 vector (gift from Keith Joung, Addgene plasmid #43860; http://n2t.net/addgene:48360; RRID: Addgene_43860), and their editing efficiencies assessed by transfecting into HEK293 cells alongside a Cas9-GFP-encoding plasmid using the X-tremeGENE 9 DNA Transfection Reagent (Merck), and genomic DNA isolated using the NucleoSpin Tissue Kit (Macherey Nagel) after 48 h of culture. The edited sites were amplified by PCR and purified using the NucleoSpin Gel and PCR Clean-up Kit (Macherey Nagel) before they were sequenced by Sanger sequencing (Eurofins Genomics). Sequence traces were analyzed by sequence-trace decomposition using TIDE (Brinkman *et al*, 2014), and active gRNAs identified. We identified the following gRNAs to generate lysosomal storage disease iPS cells (5'→3', edited base in bold, PAM sequence follows hyphen): CLN3$^{\text{D416G}}$ (ACCTGCATCTCTG**A**CACACT-GGG), and MLIV$^{\text{IVS3-2A>G}}$ cells (GCAGGCAACGCCAGGTACTGGGG). The primers used to sequence the edited sites were (5'→3'): CLN3$^{\text{D416G}}$ (AGTGCCTCA ACCTGGTGTTC, CCATGGATAAAATCGGCATT) and MLIV$^{\text{IVS3-2A>G}}$ (CGCAGCCTACACGCGGGAGCA, GCTCCCAA CAGTGAAGCCTC). Repair templates were designed as the mutated nucleotide flanked by 50 base-pairs and ordered as Ultramer DNA Oligonucleotides (IDT). The human female episomal iPSC line A18944 (ThermoFisher) was quality controlled for suitability for differentiations and to rule out common chromosomal abnormalities. For gene editing in iPSCs, the cells were split into single cells using Accutase (ThermoFisher) onto Geltrex (ThermoFisher)-coated vessels, and supplemented with rock inhibitors (Y27632, Selleckchem). Two days later, the cells were dissociated into single cells, and 2 million cells electroporated by 2x20 ms, 65V pulses using the ECM830 system (BTX) with 30 µg (pSpCas9(BB)-2A-Puro (PX459) V2.0 (gift from Feng Zhang; Addgene plasmid #62988; http://n2t.net/addgene:62988; RRID: Addgene_62988 (Ran *et al*, 2013) 5 mg MLM3636-sgRNA, and 30 µg repair template. Cells were seeded onto Geltrex (ThermoFisher)-coated 10 cm cell culture dishes and cultured in StemFlex medium (ThermoFisher). From days 2-5, electroporated cells were selected for by Puromycin (350 ng/µL; VWR) treatment (Steyer *et al*, 2018), before the cells were allowed to recuperate without Puromycin from day 5 onwards, with supplementation of rock inhibitors and RevitaCell Supplement (ThermoFisher) depending on density and colony formation. Colonies were picked individually into 96-well plates and analyzed for presence of desired mutations. This was done by lysis and gDNA extraction, PCR amplification of the edited site, and restriction fragment length polymorphism (RFLP) analysis. MLIV$^{\text{IVS3-2A>G}}$-edited sites were amplified with the primers previously used for sequencing the edited site, while CLN3$^{\text{D416G}}$ was screened for using primers permitting RFLP analysis of the edited site (GTGATGAGCACCGGGAGTTTACAATGGCGG, GGAGCACAGTTCATGGAGGG). RFLP analysis was next performed using MwoI to screen for CLN3$^{\text{D416G}}$ and KpnI to screen for MCOLN1$^{\text{IVS3-2A>G}}$ (all enzymes from NEB). PCR products showing presence of the desired restriction site were sequenced by Sanger sequencing using the aforementioned primer pairs, and homozygously edited clones selected for expansion.

Quality control of edited iPSCs

Quality controls included confirming edited sequencing traces of propagated clones, assessing puromycin tolerance to exclude Cas9 vector integration, ensuring absence of undesired on-target (Weisheit *et al*, 2020) editing events and partial chromosome 20 triplications assessed by quantitative genomic PCR (qgPCR),

sequencing the top 5 predicted CFD and MIT off-target sites, staining for pluripotency markers by immunocytochemistry, and molecular karyotyping to interrogate chromosomal abnormalities (Weisheit *et al*, 2021). Puromycin tolerance was assessed upon performing a single-cell split of iPSCs onto Geltrex-coated 6-well wells, and treating the iPSCs with Puromycin (350 ng/µL; VWR) for 3 days. Selected clones died within the three days of Puromycin treatment. Adverse on-target editing events and chromosome 20 triplications were assessed as previously described (Amps *et al*, 2011; Weisheit *et al*, 2020). In short, genomic DNA was isolated using the NucleoSpin Tissue Kit (Macherey Nagel), and subsequently analyzed by qgPCR. The human TERT TaqMan Copy Number Reference Assay (ThermoFisher 4403316) was used as an internal control, and the BCL2L1 copy number probed using the primer set (GGTGGTTGACTTTCTCTCCTAC, TCTCCGAT TCAGTCCCTTCT), and the probe 56-FAM/TGTGGAAGA/ZEN/GAACAGGACTGA GGC/3IABkFQ for detection. To assess on-target editing effects, the same reference probe was used as for chromosome 20 qgPCR, alongside primer/probes targeting the edited site: For CLN3^{D416G}, the copy number of the edited site was assessed with the following primer set for amplification (GCATCTACCTCGT CTTCCTGA, CTCCCCAAGTGGGAGACAAT), and the probe 56-FAM/TTGCCTCTGCATGACTTCCTCTGC/3IA BkFQ for detection. For the MLIV$^{IVS3\text{-}2A>G}$ locus integrity assessment, on-target editing was not assessed by qgPCR, due to the presence of a silent, heterozygous SNP within the same sequencing trace as the edited base (rs111592394, G>T), present in the wild-type A18944 iPSCs. Presence of the heterozygous SNP alongside the edited base upon Sanger sequencing thereby indicated the presence of two edited chromosomes, ruling out larger chromosomal deletions and ensuring integrity of the edited locus. Using the same genomic DNA, we also performed fingerprinting analysis to confirm the shared lineage of the edited cells and their wild-type counterparts. This was done by PCR analysis of the D1S80 locus using the following primer combination: GTCTTGTTGGAGATGCACGTGCCCCTTGC, GAAACTGGCCTCCAAACACTGCCCGCCG. The detailed protocol has previously been described (Weisheit *et al*, 2021). The top 5 predicted off-target sites (by CFD and MIT algorithms) were also amplified and Sanger sequenced, finding no off-target editing events in the edited cells. For staining for pluripotency markers, iPSC colonies were with anti-SSEA4 (ab16287, 1:500), rabbit anti-NANOG (D73G4, 1:500), mouse anti-Tra160 (MAB4360, 1:500), and rabbit anti-Oct4 (S090023, 1:500) as described earlier (Paquet et al. 2016). Selected clones showed uniform expression of all interrogated pluripotency markers. Finally, molecular karyotyping analysis was performed by isolating genomic DNA using the NucleoSpin Tissue Kit (Macherey Nagel), and analyzing it using an Illumina BeadArray scanned with an Illumina iScan. Samples with call rates below 95% were excluded, and only SNPs with a GenTrain score above 0.7 included for analysis (Weisheit *et al*, 2021). The clones used for differentiations and further experiments passed all of the aforementioned quality control checkpoints.

Differentiation and staining of lysosomal storage disease iPSC-derived cortical neurons

iPSC-derived human cortical neurons were differentiated as previously described (Paquet *et al*, 2016) with modifications. WT and gene-edited A18944 iPSCs were expanded for neuronal inductions in Essential 8 Flex medium and split into single-cells using Accutase for 8 min at 37°C at day in vitro 0 (DIV0). Upon dissociation, F12 medium was added to neutralize Accutase, cells were triturated, counted, and centrifuged (1000 rpm, 4 min). Cells were resuspended in neuronal induction (NI) medium, consisting of neuronal maintenance (NM) medium (50% Neurobasal, 50% DMEM/F12, 0.1 mg/mL penicillin-streptomycin, 0.5X B27 supplement, 0.5X N-2 supplement, 2 mM GlutaMAX, 0.1 mM non-essential amino acids, 5 µL insulin, 0.1 mM β-mercaptoethanol), SB431542 (10 µM; Selleckchem), LDN193189 (250 nM; Selleckchem) and Rock Inhibitor (RI) Y27632 (10 µM; Selleckchem) and plated at 1 million cells per of a Geltrex-coated 12-well plate. Cells were subsequently fed daily by complete NI medium exchange without RI. At DIV8, cells were split into single cells using Accutase for 10 min at 37°C before the Accutase was neutralized with F12 medium. The cells were triturated, counted, and centrifuged (1000 rpm, 4 min). Cells were resuspended in NI medium supplemented with RI at 30 million cells/mL, and 350 µL droplets were plated onto dried, crystallized poly-L-ornithine (Sigma-Aldrich)/laminin-coated 6-well plate wells. Cells were allowed to attach for 1 hour before the wells were filled with NI/RI. Media replaced daily with NI. At DIV11, culture medium was changed to NM. At days DIV11 and DIV12, NM was supplemented with bFGF (100 ng/mL; StemCell Technologies). Two days prior to neural rosette isolation, NM was again supplemented with bFGF to boost the expansion of neural rosettes. Upon appearance of neural rosettes (around DIV23), cells were incubated for 1 h in STEMdiff neural rosette

selection reagent (NRSR; StemCell Technologies) at 37ºC. NRSR was replaced with NM, and rosettes manually isolated while excluding the edges of the spots, containing non-rosette cells. Rosettes were collected, triturated into smaller clumps, and centrifuged (1000 rpm, 4 min), before they were resuspended in NM supplemented with bFGF at a ratio of 3:2. The rosettes were seeded on fresh poly-L-ornithine/laminin-coated 6-well plates, and the medium replaced with NM+bFGF the following day. The rosettes were fed daily with NM, and at around DIV32 split with Accutase for 4 min, neutralized with NM, centrifuged (1000 rpm, 4 min), and resuspended in NM for seeding into new poly-L-ornithine/laminin-coated 6-well plates at a ratio of 1:3. At DIV42, neural rosettes were either frozen in NM supplemented with bFGF and 10% DMSO for long-term storage or split for terminal differentiation into mature cortical neurons.

For terminal maturation, the neural rosettes were split into single cells using Accutase for 10 min, Accutase neutralized using NM, triturated, centrifuged (1000 rpm, 4 min), resuspended in Neurobasal medium supplemented with B27, penicillin/streptomycin, and glutamine (from here on termed NB/B27), filtered through 40 µm strainers, and counted. NB/B27 medium was added to seed 400,000 cells/12 mm coverslip, or 200,000 cells/Ibidi 8-well plate well. Coverslips and Ibidi 8-well plate wells were freshly coated with poly-L-ornithine/laminin prior to neuronal seeding. NB/B27 medium was replaced as half feeds every 2-3 days. The cells were supplemented with the γ-secretase inhibitor DAPT for the first 7 days after plating to augment neuronal maturation, and with 5-fluorouracil (5-FU) for days 2-7 to prevent expansion of contaminating, proliferating cells (predominantly NPCs and astrocyte precursors). At day 7 after seeding, culture vessels were agitated to dislodge cell debris, and the medium completely replaced with NB/B27 without DAPT or 5-FU. From 7 days after plating onward, the neurons were kept in NB/B27 medium without DAPT or 5-FU until experiments were performed. Unless otherwise stated, reagents used for cortical neuron differentiation were obtained from ThermoFisher.

Following a week after terminal differentiation, the cortical neurons were stained for the neuronal markers β3-Tubulin/TuJ1, MAP2, and CTIP2. Neurons were fixed with 4% PFA in PBS for 30 min, washed twice with PBS, and blocked for 1 hour with blocking buffer (PBS with 3% donkey serum, 0.1% Triton X100, and 0.02% w/v sodium azide). Following blocking, cells were stained overnight at 4°C with the following antibodies: chicken anti-MAP2 (ab5392; 1:1000), mouse anti-Tuj1 (MMS-435P; 1:500) and rat anti-CTIP2 (ab18465; 1:200). The following day, cells were washed three times with PBS and stained with secondary antibodies for 2 h at room temperature. The following secondary antibodies were used: Donkey anti-mouse Alexa488 (A32766; 1:500), goat anti-rat Alexa555 (A21434; 1:500), and goat anti-chicken Alexa647 (A32933; 1:500). Cells were washed once with PBS, stained with DAPI for 20 min (ThermoFisher, 1:50,000 in PBS), and washed three times with PBS before being mounted on microscope slides for confocal imaging. Images were captured using a Zeiss Confocal microscope (Zeiss LSM880) equipped with a 40X oil objective, exciting at 405 nm (DAPI), 488 nm (Alexa488-conjugated 2° antibodies), 561 nm (Alexa568-conjugated 2° antibodies), and 633 nm (Alexa647-conjugated 2° antibodies).

Pharmacokinetic study of TPC2-A1-P in C57Bl/6N mice

Levels of the compound were determined by LC-MS/MS in blood plasma and brain samples over time after a single dose. The following reagents and consumables were used: DMSO Chromasolv Plus, HPLC grade, ≥99.7% (Sigma-Aldrich, USA; Cat #34869), Acetonitrile Chromasolv, gradient grade, for HPLC, ≥99.9% (Sigma-Aldrich, USA; Cat #34851), Methanol Chromasolv Plus, for HPLC, ≥99.9% (Sigma-Aldrich, USA; Cat 34860), Polyethylene glycol (PEG400) (AppliChem, Germany, Lot# 2S008911, CAS#25322-68-3), Blood collection microtubes, EDTA K2/K3, Purple, 0.5ml (Jiangxi, China, Code # NLD907), 2,2,2-Tribromoethanol 97% (Sigma-Aldrich; Cat # T48402), Compound IS-2015 was used as internal standard (IS), DMSO-PEG400-physiological saline (20%:50%:30%, v/v) was used as formulation vehicle. TPC2-A1-P was dissolved in DMSO and vortexed for 2 min. PEG400 was next added and the mixture was vortexed for 1 min. Next, saline was added and vortexed for 1 min, resulting in a clear solution. The batch of the working formulation was prepared 1 h prior to the in vivo study. The following equipment was used for compound detection: Gradient HPLC system (Shimadzu, Japan), MS/MS detector API 3000 PE with TurboIonSpray Electrospray module (PE Sciex, Canada), VWR Membrane Nitrogen Generators N2-04-L1466, nitrogen purity 99%+ (VWR, USA). Male C57Bl/6N mice aged 10 weeks were used in this study. The animals were randomly assigned to the treatment groups and fasted for 4 h before dosing. Six time points (5, 30, 60, 240, 480 and 1440 min) were set

for this pharmacokinetic study. Each time-point treatment group included 4 animals. There was also one vehicle dosed animal. Mice were injected i.p. with 2,2,2-tribromoethanol at the dose of 150 mg/kg prior to drawing the blood. Blood collection was performed from the orbital sinus in microtainers containing K2EDTA. Animals were next sacrificed by cervical dislocation after the blood samples collection. All samples were immediately processed, flash-frozen and stored at -70°C until subsequent analysis. Plasma samples (50 µl) were mixed with 200 µl of internal standard (IS) solution. After mixing by pipetting and centrifuging (4 min, 6000 rpm), 1 µl of each supernatant was injected into the LC-MS/MS system. A solution of compound IS-2015 (400 ng/ml in acetonitrile-methanol mixture, 1:1, v/v) was used as IS for quantification of TPC2-A1-P in plasma samples. Brain samples (weight 200 mg ± 1 mg) were dispersed in 800 µl of IS400(80) using zirconium oxide beads (115 mg ± 5 mg) in The Bullet Blender homogenizer for 30 seconds at speed 8. Samples were centrifuged (4 min, 14,000 rpm), and 1 µl of each supernatant injected into LC-MS/MS system. A solution of compound IS-2015 (400 ng/ml in water-methanol mixture, 1:4, v/v) was used as an internal standard (IS400(80)) for quantification of TPC2-A1-P in brain samples. Analyses of plasma and brain samples were conducted by the bioanalytical laboratory personnel at Enamine/Bienta. The concentrations of each compound in the blood plasma and brain were determined using high performance liquid chromatography/tandem mass spectrometry (HPLC-MSMS). The Shimadzu HPLC system consisted of controller Prominence CBM20A2, isocratic pumps LC-10ADvp, an autosampler Prominence SIL-20AC, a sub-controller FCV-14AH, and a degasser DGU-14A. Mass spectrometric analysis was performed using an API 3000 (triple-quadrupole) instrument from AB Sciex (Canada) with an electro-spray (ESI) interface. The data acquisition and system control was performed using Analyst 1.5.2 software (AB Sciex, Canada). Calibration standards for quantification of compound in plasma samples: TPC2-A1-P was dissolved in DMSO at concentration of 2 mg/ml (stock solution). A series of calibration standards was prepared by serial dilution of stock solution with blank mouse plasma to a final concentration ranging from 20 to 20,000 ng/ml. Standard plasma samples (50 µl) were mixed with IS (200 µl). After mixing by pipetting and centrifuging for 4 min at 6000 rpm, 1 µl of each supernatant was injected into LC-MS/MS system. The TPC2-A1-P stock solution (see description above) was consecutively diluted with IS400(80) to get a series of calibration solutions with final concentrations ranging from 5 to 2000 ng/ml. The calibration curve was constructed using blank mouse brain samples. To obtain calibration standards, blank brain samples were homogenized in 800 µl of corresponding calibration solution using zirconium oxide beads (115 mg ± 5 mg) in The Bullet Blender® homogenizer (30 seconds, speed 8). After that, the samples were centrifuged for 4 min at 14000 rpm, and 1 µl of each supernatant was injected into LC-MS/MS system. The regression analysis of TPC2-A1-P was performed by plotting the peak area ratio (y) against the compound concentration in calibration standards (x, ng/ml or ng/g). The validity of the calibration curves (relationship between peak area ratio and compound concentration) was ensured by the correlation coefficients (R) calculated for the quadratic regression. The concentrations of TPC2-A1-P in plasma and brain samples below the lower limit of quantitation (Plasma LLOQ – 20 ng/ml; Brain LLOQ – 20 ng/g) were designated as zero. The pharmacokinetic data analysis was performed using non-compartmental, bolus injection or extravascular input analysis models in WinNonlin 5.2 (PharSight).

Site-directed mutagenesis and colocalization analysis using confocal microscopy

All human CLN3 mutants were generated from WT cDNA templates using QuikChange Site-Directed Mutagenesis Kit (Stratagene) and verified by sequencing both strands entirely. For site-directed mutagenesis of CLN3 mutants L101P, S131R, C134R, A158P, G165E, L170P, I285V, L306H, V330F and V330I, the KAPA HiFi Hotstart Ready Mix (Roche) was used and the already mentioned plasmid pcDNA6.2/N-EmGFP-DEST with hCLN3 as a template and (10 ng of plasmid DNA in a 25 µl reaction) and primers that had been obtained from Eurofins Genomics (see Table S2 for primer sequences). PCR was done with a Mastercycler® nexus gradient (Eppendorf) PCR conditions were set to an initial denaturing step at 95°C for 3 min, followed by 16 cycles containing of a denaturing step at 98°C for 20 sec, an annealing step at 68°C for 1 min, and an elongation step at 72°C for 7 min, followed by an additional elongation step at 72°C for 7 min. After PCR amplification, the mix was digested for 2 h at 37°C with FastDigest DpnI and 100 µl of XL1Blue E. coli cells (Agilent) were transformed with 5 µl of the digested mix by incubating them for 20 min on ice, heat-shocking them for 40 sec at 37°C, incubating them another 2 min on ice, pre-culturing them for 1 h at 37 °C in a shaker at 200 rpm after addition of 900 µl LB(+) medium, and plating 250 µl of pre-culture on an ampicillin-

containing agar plate. After colony growth, colonies were cultured for 16 h at 37°C at 200 rpm in a shaker and plasmid DNA was isolated with a CompactPrep Plasmid Mini Kit. Isolated plasmid DNA was sequenced to check for successful mutagenesis. Mutants G187A, G189R, G189W, V290L, E295K, R334C, R334H, Q352H, R405W and D416G have been generated using the QuikChange Site-Directed Mutagenesis Kit (Stratagene). Sequencing of plasmid DNA was done by Eurofins Genomics. In case of successful clonation, a Midi preparation of the respective plasmid DNA was done using the CompactPrep Plasmid Midi Kit. DNA concentration was measured with a NanoDrop™ 2000c spectrophotometer. All CLN3 WT and mutant isoforms were N-terminally tagged GFP versions. For functional studies constructs were transiently expressed in CLN3$^{-/-}$ HeLa cells with the use of Turbofect (ThermoFisher) and analysed 24-48 h after transfection using a confocal microscope (Zeiss LSM 880). Colocalization analysis was done with the JACoP plugin of FiJi. For each cell, a region of interest (ROI) was selected and the channels were separated. For MCC (Mander's correlation coefficient) values, fixed thresholds were set for each transfected plasmid (GFP-CLN3: 92, LAMP1-RFP: 86, Rab5-RFP: 84, Rab11-DsRed: 77, MitoTracker-DR: 122).

Endolysosomal patch-clamp experiments

For whole-LE/LY manual patch-clamp recordings, cells were treated with apilimod (neurons). Compounds were washed out before patch-clamp experimentation. Unless otherwise stated, the cytoplasmic solution contained 140 mM K-MSA, 5 mM KOH, 4 mM NaCl, 0.39 mM $CaCl_2$, 1 mM EGTA and 20 mM HEPES (pH was adjusted with KOH to 7.2) and luminal solution contained 140 mM Na-MSA, 5 mM K-MSA, 2 mM Ca-MSA, 1 mM $CaCl_2$, 10 mM HEPES and 10 mM MES (pH was adjusted with NaOH to 4.6) were used. For the application of small molecule agonists (ML-SA1, ML1-SA1 (EVP169)), cytoplasmic solution was completely exchanged by cytoplasmic solution containing agonist. Intact endolysosomes were manually isolated as described before (Chen *et al*, 2017). Currents were recorded using an EPC-10 patch-clamp amplifier (HEKA, Lambrecht, Germany) and PatchMaster acquisition software (HEKA). Data were digitized at 40 kHz and filtered at low-pass filter frequency of 2.9 kHz. Recording glass pipettes were polished and had a resistance in range of 8-11 MΩ. Fast and slow capacitive transients were cancelled by the compensation circuit of the EPC-10 amplifier. Mean capacitance value for LE/LY of fibroblasts was 0.5 ± 0.1 (SEM; n=4). In all experiments, 500-ms voltage ramps from -100 to +100 mV were applied every 5 s, holding potential was kept at 0 mV. The current amplitudes at -100 mV were extracted from individual ramp current recordings. Offline analysis was performed with the software Origin8 (OriginLab Corp., Northampton, MA, USA).

Knockdown of TPC2 (siRNA)

Human fibroblasts have been electroporated using Neon Transfection System with 50nM of siRNA targeting a control sequence (ON-TARGETplus Non-targeting Pool, D-001810-10-05) and siRNA targeting TPCN2 ON-TARGET plus human TPCN2 (219931) siRNA SMARTpool, L-006508-00-0005). Cells were incubated with siRNA for 72h. Knockdown efficiency have been estimated by using Real-time quantitative PCR.

Real-time quantitative PCR analysis

To assess expression levels of the target channels and disease genes, we used real-time quantitative PCR (RT-qPCR). RNA was isolated from iPSC-derived neurons at day 10 after terminal maturation using the RNeasy Mini Kit (Qiagen), following manufacturer's instructions. RNA was immediately synthesized into cDNA and stored at -80ºC. cDNA was synthetized using the RevertAid First Strand cDNA Synthesis Kit (ThermoFisher), using 50 ng RNA as template for cDNA synthesis. cDNA synthesis was primed using a 1:1 combination of random hexamers and oligo(dT) primers, and cDNA synthesis initiated by a 5-minute incubation at 25ºC followed by a 60-minute incubation at 42ºC. The reaction was terminated by heating at 70ºC for 5 min, the cDNA diluted 1:7 with nuclease-free water, and frozen at -20ºC for storage. For RT-qPCR of cDNA isolated from iPSC-derived neurons, we employed the SYBR Green system (ThermoFisher) and the LightCycler 480 Instrument (Roche). Alternatively, for the prepared Human Brain cDNA Array (OriGene), we used the SYBR Green system and a CFX96 instrument (Biorad) to accommodate the pre-aliquoted cDNA plate. The primer pairs used for human samples were as follows (5'→3'): TRPML1 (TCTTCCAGCACGGAGACAAC, GCCACATGAACCCCACAAAC), TRPML2 (AACGGTGTTTCCTGTTCCGA, GCCATTGCATTTCTGA CGGTTA), TRPML3 (TGCTTCTGTGGATGGATCG, GAGACCATGTTCAGAGAACG), TPC1 (TCCCA

AAGCGCTGAGATTAC, TCTGGTTTGAGCTCCCTTTC), TPC2 (GTACCCCTCTTGTGTGGACG, GGCCCTGACAGTGACAACTT), CLN3 (GGTTCTCGTCAGTGGGATTT, CTGATGAGATGCTAGCGAAGAC), EMC7 (Eisenberg & Levanon, 2013; Artyukhov *et al*, 2017) (AAAGGAGGTAGTCAGGCCGT, GTTGCTTCACACGGTTTTCCA). For detecting mouse transcripts from biopsies, sample preparation and detection was performed as previously described, but transcripts detected with the following primers: TPC2 (TAAAGTACCGCTCCATCTACCA, GCAGACGTTCGAG TAATACCAG), HPRT (Hruz *et al*, 2011) (GCTCGAGATGTCATGAAGGAGAT, AAAGAACTTATAG CCCCCCTTGA). For iPSCs we opted for EMC7 as our preferred housekeeping gene due to consistent expression throughout neuronal differentiation (Eisenberg & Levanon, 2013; Artyukhov *et al*, 2017; Burke *et al*, 2020)

Magic Red Cathepsin B activity measurements

Lysosomal protease activity was measured using the Magic Red Cathepsin B Kit (AbD Serotec), which utilizes a cathepsin B target sequence (RR), fused by amide bonds to the fluorophore cresyl violet and quenching its fluorescence. Proteolytic cleavage of the quenching target sequence thus increases the cresyl violet fluorescence, which can be detected by confocal imaging. We used a fluorescence recovery after photobleaching (FRAP) approach previously utilized for assessing proteolysis upon CLN3 knockdown (Metcalf *et al*, 2008) to assess proteolysis in iPSC-derived neurons. The Magic Red Cathepsin B Kit was prepared according to manufacturer's instructions and the iPSC-derived neurons loaded for 60 min at 37ºC before imaging with a Zeiss LSM880 confocal microscope, equipped with a 37°C incubation unit and a 63X water immersion objective. FRAP bleaching was performed using 514 nm, 561 nm, and 633 nm lasers targeted towards the most intensely labelled area of the neuronal soma at 100% intensity for 200 iterations (85 s). The pinhole was kept wide (4.70 AU) to image throughout several focal planes, avoiding focal plane shifts or vesicular movement in the z-direction to influence the signal. Next, fluorescence recovery was measured upon excitation with a DPSS 561 nm laser, recording images every 75 ms for 75 seconds. Fluorescence recovery within photobleached regions was finally quantified relative to the initial fluorescence after photobleaching. For compound treatment of iPSC-derived neurons, compounds were administered 48 h prior to loading Magic Red Cathepsin B.

Lysosomal exocytosis experiments

Human fibroblasts (2 x 10^4) were seeded on 8-well plates (Ibidi) and cultured overnight. Cells were washed once with Minimum Essential Media (MEM) supplemented with 10 mM HEPES and then treated with TPC2-A1-P (30 µM) or ML-SA1 (30 µM) for 90 min. Ionomycin (4 µM for 10 min) and ML-SA1 were used as positive controls. Following treatment, cells were incubated with an anti-LAMP1 antibody (1:200, SantaCruz) in MEM supplemented with 10 mM HEPES and 1% BSA for 20 min on ice. Cells were then fixed with 2.6% PFA (ThermoFisher) for 20 min and incubated with Alexa Fluor 488 conjugated secondary antibody (ThermoFisher) for 1 h in PBS containing 1% BSA. Nuclei were stained with DAPI. Confocal images were acquired using an LSM 880 microscope (Zeiss) with 40X magnification. Plasma membrane LAMP1 mean intensity was calculated using unsaturated images on ImageJ 1.52a software. For the flow cytometry assay human fibroblasts (wild type, MLIV, NPC1 and JNCL) (15 x 10^4) were seeded overnight in a 6 well plate. Cells were washed once with Minimum Essential Media (MEM) supplemented with 10 mM HEPES and then treated with TPC2-A1-P (30 µM) for 90 min. Ionomycin (4 µM for 10 min) was used as positive control. Following treatment, cells were collected in falcon tubes and incubated on rotation with an anti-LAMP1 antibody (1:200, SantaCruz) in MEM supplemented with 10 mM HEPES and 1% BSA for 20 min at 4°C. Cells were then fixed with 2.6% PFA (ThermoFisher) for 20 min and incubated with Alexa Fluor 488 conjugated secondary antibody (ThermoFisher) for 1 h in PBS containing 1% BSA. Samples were then resuspended in DPBS 1 X and before FACS analysis cells were filtered with a pre-separation filter with a cut off of 20/30 µm. The instrument used was BD FACS Aria III.

Construction of the TPC2-IRES-Cre targeting vector (to generate a TPC2 reporter mouse line) and gene targeting

The final targeting construct is comprised of a 5' TPC2 homology arm, an IRES-Cre-FRT-pgk-Neo-FRT cassette and a 3' TPC2 homology arm. The 2665-bp 3' homology arm containing sequence downstream of the

final exon of Tpcn2 (exon 25) was amplified by polymerase chain reaction (PCR) from genomic R1 mouse embryonic stem (ES) cell DNA. The fragment was then subcloned into pKO-DTA using a 5' AscI site and a BamHI site localized at the 3'end, which were incorporated within the primer sequences. In a similar manner, the 2332-bp 5' homology arm containing the stop codon of TPC2 was generated and also cloned into the vector using XhoI and AscI sites. PCR amplification of both homology arms was undertaken using the high-fidelity PfuUltra II DNA polymerase to minimize PCR-induced mutations, and any nucleotides that differed from the database sequence upon sequence analysis were verified by independent PCR amplification and sequencing. Finally, the IRES-Cre-FRT-pgk-Neo-FRT cassette was cloned into the AscI site found at the junction of the 2 homology arms. The completed targeting construct was then further verified by a complete sequence analysis and restriction mapping. Following verification of the integrity of the targeting construct, plasmid DNA was linearized using the NotI enzyme and then electroporated into R1 ES cells at the FARAH Mammalian Transgenics Platform, University of Liege. Following electroporation, correctly targeted clones were identified by Southern blot analysis and these were then used to generate mice following standard protocols (injection of ES cells [129/Sv] into blastocysts [C57BL/6], implantation of injected blastocysts into foster mothers, backcross of male chimeras with C57BL6 females). F1 animals resulting from backcrosses were then crossed with FLP-deleter mice, which contain a ubiquitously expressed FLP recombinase gene, to facilitate removal of the FRT-flanked neomycin selection cassette.

Generation and analysis (by immunohistochemistry) of the TPC2 reporter mouse line

Mice harboring the Tpcn2$^{IRES\text{-}Cre}$ locus were bred with ROSA26-floxed-stop-τGFP mice, giving rise to mice constitutively expressing τGFP under control of the TPC2 promoter. The Tpcn2$^{IRES\text{-}Cre}$ mouse serves the purpose of expressing Cre recombinase cDNA under control of the TPC2 promoter, without affecting protein function (Mountford & Smith, 1995). The latter mouse harbors a transgene insertion on the *ROSA26* locus, consisting of a loxP-flanked (floxed) polyadenylation termination sequence followed by cDNA of the microtubule-associated protein tau (τ), conjugated to GFP. Without co-expression of Cre recombinase, the transgene is silenced by virtue of the floxed transcriptional termination signal. However, TPC2 promoter-driven Cre recombinase expression excises the termination signal, facilitating constitutive τGFP expression (see Fig. 8A) (Wyatt *et al*, 2017). The fusion of GFP to τ furthermore permits GFP distribution throughout neurites, enabling identification of expressing neurons (Wen *et al*, 2011; Iwata *et al*, 2019). At 11 weeks of age, the mice were anaesthetized upon intraperitoneal injection of ketamine and xylazine, and a needle inserted into the left ventricle. A small incision was made in the right atrium for liquid to leave the body. The circulation was first flushed with PBS before 4% PFA was injected into the mouse. Organs were removed and separately post-fixated in 4% PFA for 6 h at 4°C. PFA was next aspirated, and the organs stored overnight in 18% sucrose solution at 4°C. Organs were next embedded in OCT medium, first for 4 h at room temperature, then in embedding molds in isobutane beakers surrounded by ethanol and dry ice. Frozen embedding molds were stored at -80°C until slicing. For slicing, embedding molds were thawed to -16°C, and sliced into 14 µm thick slices. Slices were stored at -80°C until further use. For immunohistochemistry, slices were thawed to room temperature for 15 min, and washed three times with PBS. The slices were next blocked for 1 hour (10% normal donkey serum, 3% bovine serum albumin, 0.3% Triton X-100), and primary antibody staining solutions added at 4°C overnight. The following primary antibodies and dilution factors in PBS were used: chicken anti-GFP (Invitrogen, A10262; 1:1000), rabbit anti-β3 tubulin (Abcam, ab18207; 1:500), rat anti-CD13 (Abcam, ab33489; 1:200), rabbit anti-Iba1 (WAKO, 019-19741; 1:100), Cy3-conjugated mouse anti-GFAP (Sigma Aldrich, C9205; 1:500), and rabbit anti-mGluR1 (Alomone, AGC-006; 1:100). Slices were washed three times with PBS, and next stained with secondary antibodies for 2 h at room temperature. The following secondary antibodies and dilution factor in PBS were used: anti-chicken Alexa488 (Invitrogen, A11039; 1:500), anti-rabbit Cy3 (Jackson Dianova, 711-165-152; 1:500), and anti-rat Cy3 (Jackson Dianova, 112-165-143; 1:500). Samples were washed once with PBS, stained with DAPI for 30 min, washed three times with PBS, and mounted for imaging using a Zeiss LSM880 confocal microscope, equipped with a 40X oil immersion objective.

Autophagy assays

For western blot analysis, the following antibodies were used: β-Actin (Santa Cruz SC 47778, 1:4000), LC3 (Novus NB100-2220, 1:1000) P62/SQSMT1 (BD 610833, 1:1000), Vinculin (Cell Signaling Technology, 1:1000, cat. #4650). Total cell lysate was prepared by solubilization in TRIS HCl 10 mM pH 8.0 and 0.2% SDS supplemented with protein and phosphatases inhibitor (Sigma). Protein concentration was determined by the Bradford method (Biorad). After SDS-polyacrylamide gel electrophoresis (PAGE) and immunoblotting, the protein recognized by the specific antibody were visualized by chemiluminescence methods (Luminata Crescendo Western HRP substrate, Millipore) using peroxidase-conjugated anti-rabbit or anti-mouse secondary antibodies (Cell Signaling Technology). Membranes were developed using an Odyssey imaging system (LI-COR Biosciences). Quantification was carried out using unsaturated images on ImageJ 1.52a software.

Electron microscopy experiments

Human fibroblasts or neuronal stem cells were seeded in 6-well plates and treated with 30 µM TPC2-A1-P or DMSO for 48h. The cells were fixed with 1% glutaraldehyde for 30 min at room temperature, then washed with 1X PBS and post-fixed as previously described (Polishchuk *et al*, 2019). After dehydration, the specimens were embedded in epoxy resin and polymerized at 60 °C for 72 h. Thin 60 nm sections were cut using a Leica EM UC7 microtome. EM images were acquired from thin sections using a FEI Tecnai-12 electron microscope equipped with a VELETTA CCD digital camera (FEI, Eindhoven, NL). Each EM experiment was repeated three times and 20 fields of view were analyzed for each sample. Double-blind analysis of the samples was performed: number of inclusions (fingerprint-like structures/area), % cytosol covered by inclusions (area of inclusion/cell area), and mitochondria numbers/ area as well as mitochondria cristae numbers/area.

In vivo experiments in MLIV mice

Animals were used under animal protocols approved by the government (Regierung von Oberbayern, ROB-55.2-2532.Vet_02-17-170) and University of Munich (LMU) Institutional Animal Care Guidelines or in accordance with the guidelines and policies of the European Communities Council, approved by the Italian Ministry of Health. Mice were housed in individually ventilated cages in rooms maintained at constant temperature (20-24°C) and humidity (45-65%) with a 12 hour light cycle. Animals were allowed food and water ad libitum.

Experiments were performed with previously described MLIV ($Mcoln1^{tm1Sasl}$, i.e. $Mcoln1^{\Delta Ex3\text{-}5}$) mice and littermate controls (Venugopal *et al*, 2007). Mice were genotyped as initially described using gDNA extracted from ear clips (Venugopal *et al*, 2007). The injection solutions were prepared in pre-warmed PBS in a sterile laminar flow hood. 4-week old mice were injected with either vehicle (PBS with 10% DMSO and 10% PEG-400) or vehicle plus 20 mg/kg TPC2-A1-P i.p. every day for 13 weeks using a 25G needle. The appearance and body weight of injected mice was scored on a weekly basis. No gross scoring differences were observed between vehicle and TPC2-A1-P-injected mice until the experimental endpoint. Following the course of injections, mice were euthanized as previously described for the TPC2 reporter mouse, and brains retrieved for immunohistochemistry.

Furthermore, male and female WT (n = 10, 5 males and 5 females) and MLIV (n = 24, 13 males and 11 females) 9-week old mice, after receiving i.p. injections every day (either vehicle or TPC2-A1-P as described above) for one week, were subjected to the open field test (day1) and to the Rotarod test (day 2). In the open field test mice were left free to explore a Plexiglas arena (35 × 47 × 60 cm) for 15 min. The distance travelled (m), the maximum speed (m/s), the immobility time (s) and the distance (m) travelled in the center of the arena (central distance) were recorded using a video camera (PANASONIC WV-BP330) and automatically scored through a video-tracking system (ANY-MAZE, Stoelting, USA). The accelerating rotarod test was applied using a commercial apparatus (Ugo Basile) consisting of a rod suspended horizontally at a height of 14 cm from the floor. The rod (5 cm in diameter) was accelerated from 4 rpm to 40 rpm in 300 s and the latency to fall from the rod was measured; animals were left to run on the maximum speed for additional 300 s. The tests were repeated four times (with an intertrial interval of 30 min) and the average latency to fall was calculated. Sex x group effects were tested using a two-way ANOVA (with sex and groups, as between factors); as no sex x groups interaction was observed for any of the measures, values between the two sexes were averaged.

Cell viability assay

Human patient fibroblasts (HF) were seeded at a cell density of 3.000 cells per well, while iPSC-derived neurons were seeded at a density of 2.000 cells per well in 96-well plates (Sarstedt). Both, HF and iPSC-derived neurons were treated the following day with different concentrations of TPC2-A1P and HF also with other reported TPC2 agonists (Zhang et al, 2019), and monitored over 24-, 48-, 72-h. DMSO was used as vehicle control and full medium as blank. Cell viability using CellTiter-Blue reagent was measured according to the manufacturer's protocol. The compounds amitriptyline (#PHR1384), chlorpromazine (#C0982) clomipramine (#C7291) and desipramine (#D3900) were ordered from Sigma Aldrich and riluzole (#0768) was ordered from Tocris. The analysis was performed with GraphPad Prism 9.1.

Immunohistochemistry on mouse brain

Brain preparation and embedding in OCT was performed as described above for the TPC2 reporter mouse. Frozen embedding molds were stored at -80°C until slicing. For slicing, embedding molds were thawed to -16˚C, and sliced into 40 µm thick slices. Slices were stored at -80°C until further use. For immunohistochemistry, slices were thawed to room temperature for 30 min, and washed three times with PBS. For staining glial markers, immunohistochemistry was performed as previously described for the TPC2 reporter mouse. For P62 immunohistochemistry, the slices were blocked for 1 hour with blocking buffer (3% BSA, 5% FBS, 0.1% Triton X-100 in PBS1X) and anti P62/SQSTM1 primary antibody was added o/n at 4°C in the same blocking buffer (1:500, GP62-C Progen). Slices were washed three times with PBS, and next stained with secondary antibodies for 1h at room temperature (1:400, A-11074 ThermoFisher). Samples were washed once with PBS, stained with DAPI for 15 min, washed three times with PBS, and mounted for imaging using a Zeiss LSM880 confocal microscope, equipped with a 10X objective, zoom 0.6.

Suppl. references

Amps K, Andrews PW, Anyfantis G, Armstrong L, Avery S, Baharvand H, Baker J, Baker D, Munoz MB, Beil S, *et al* (2011) Screening ethnically diverse human embryonic stem cells identifies a chromosome 20 minimal amplicon conferring growth advantage. *Nat Biotechnol* 29: 1132–1144

Artyukhov AS, Dashinimaev EB, Tsvetkov VO, Bolshakov AP, Konovalova E V., Kolbaev SN, Vorotelyak EA & Vasiliev A V. (2017) New genes for accurate normalization of qRT-PCR results in study of iPS and iPS-derived cells. *Gene* 626: 234–240

Brinkman EK, Chen T, Amendola M & Van Steensel B (2014) Easy quantitative assessment of genome editing by sequence trace decomposition. *Nucleic Acids Res* 42: 1–8

Burke EE, Chenoweth JG, Shin JH, Collado-Torres L, Kim SK, Micali N, Wang Y, Colantuoni C, Straub RE, Hoeppner DJ, *et al* (2020) Dissecting transcriptomic signatures of neuronal differentiation and maturation using iPSCs. *Nat Commun* 11: 1–14

Chen C-C, Cang C, Fenske S, Butz E, Chao Y-K, Biel M, Ren D, Wahl-Schott C & Grimm C (2017) Patch-clamp technique to characterize ion channels in enlarged individual endolysosomes. *Nat Protoc* 12: 1639–1658

Concordet JP & Haeussler M (2018) CRISPOR: Intuitive guide selection for CRISPR/Cas9 genome editing experiments and screens. *Nucleic Acids Res* 46: W242–W245

Eisenberg E & Levanon EY (2013) Human housekeeping genes, revisited. *Trends Genet* 29: 569–574

Hruz T, Wyss M, Docquier M, Pfaffl MW, Masanetz S, Borghi L, Verbrugghe P, Kalaydjieva L, Bleuler S, Laule O, *et al* (2011) RefGenes: Identification of reliable and condition specific reference genes for RT-qPCR data normalization. *BMC Genomics* 12: 156

Iwata M, Watanabe S, Yamane A, Miyasaka T & Misonou H (2019) Regulatory mechanisms for the axonal localization of tau protein in neurons. *Mol Biol Cell* 30: 2441–2457

Kwart D, Paquet D, Teo S & Tessier-Lavigne M (2017) Precise and efficient scarless genome editing in stem cells using CORRECT. *Nat Protoc* 12: 329–334

Metcalf DJ, Calvi AA, Seaman MNJ, Mitchison HM & Cutler DF (2008) Loss of the batten disease gene CLN3 prevents exit from the TGN of the mannose 6-phosphate receptor. *Traffic* 9: 1905–1914

Mountford PS & Smith AG (1995) Internal ribosome entry sites and dicistronic RNAs in mammalian transgenesis. *Trends Genet* 11: 179–184

Paquet D, Kwart D, Chen A, Sproul A, Jacob S, Teo S, Olsen KM, Gregg A, Noggle S & Tessier-Lavigne M (2016) Efficient introduction of specific homozygous and heterozygous mutations using CRISPR/Cas9. *Nature* 533: 125–129

Polishchuk E V, Merolla A, Lichtmannegger J, Romano A, Indrieri A, Ilyechova EY, Concilli M, Cegli R De, Crispino R, Mariniello M, *et al* (2019) Activation of Autophagy, Observed in Liver Tissues From Patients With Wilson Disease and From ATP7B-Deficient Animals, Protects Hepatocytes From Copper-Induced Apoptosis. *Gastroenterology* 156: 1173–1189

Ran FA, Hsu PD, Wright J, Agarwala V, Scott DA & Zhang F (2013) Genome engineering using the CRISPR-Cas9 system. *Nat Protoc* 8: 2281–2308

Steyer B, Bu Q, Cory E, Jiang K, Duong S, Sinha D, Steltzer S, Gamm D, Chang Q & Saha K (2018) Scarless Genome Editing of Human Pluripotent Stem Cells via Transient Puromycin Selection. *Stem Cell Reports* 10: 642–654

Venugopal B, Browning MF, Curcio-Morelli C, Varro A, Michaud N, Nanthakumar N, Walkley SU, Pickel J & Slaugenhaupt SA (2007) Neurologic, Gastric, and Opthalmologic Pathologies in a Murine Model of Mucolipidosis Type IV. *Am J Hum Genet* 81: 1070–1083

Weisheit I, Kroeger JA, Malik R, Klimmt J, Crusius D, Dannert A, Dichgans M & Paquet D (2020) Detection of Deleterious On-Target Effects after HDR-Mediated CRISPR Editing. *Cell Rep* 31: 107689

Weisheit I, Kroeger JA, Malik R, Wefers B, Wurst W, Dichgans M, Paquet D & Lichtner P (2021) Simple and reliable detection of CRISPR-induced on-target effects by qgPCR and SNP genotyping. *Nat Protoc* 16: 1714–1739

Wen S, Go IN, Mai O, Schauer C, Leinders-zufall T & Boehm U (2011) Genetic Identification of GnRH Receptor Neurons : A New Model for Studying Neural Circuits Underlying Reproductive Physiology in the Mouse Brain. 152: 1515–1526

Wyatt A, Wartenberg P, Candlish M, Krasteva-christ G, Flockerzi V & Boehm U (2017) Genetic strategies to analyze primary TRP channel-expressing cells in mice. *Cell Calcium* 67: 91–104

Zhang X, Chen W, Li P, Calvo R, Southall N, Hu X, Bryant-genevier M, Feng X, Geng Q, Gao C, *et al* (2019) Agonist-specific voltage-dependent gating of lysosomal two-pore Na+ channels. *Elife* 8: e51423
