# Supplementary material for: TPC2 rescues lysosomal storage in mucolipidosis type IV, Niemann–Pick type C1, and Batten disease
Source: EMBO Mol Med. 2022 Aug 5;14(9):e15377. doi: 10.15252/emmm.202115377 (PMC9449600; doi:10.15252/emmm.202115377)
Supplement: Supplementary file 2 — Expanded View Figures PDF [file EMMM-14-e15377-s001.pdf]

Expanded View Figures

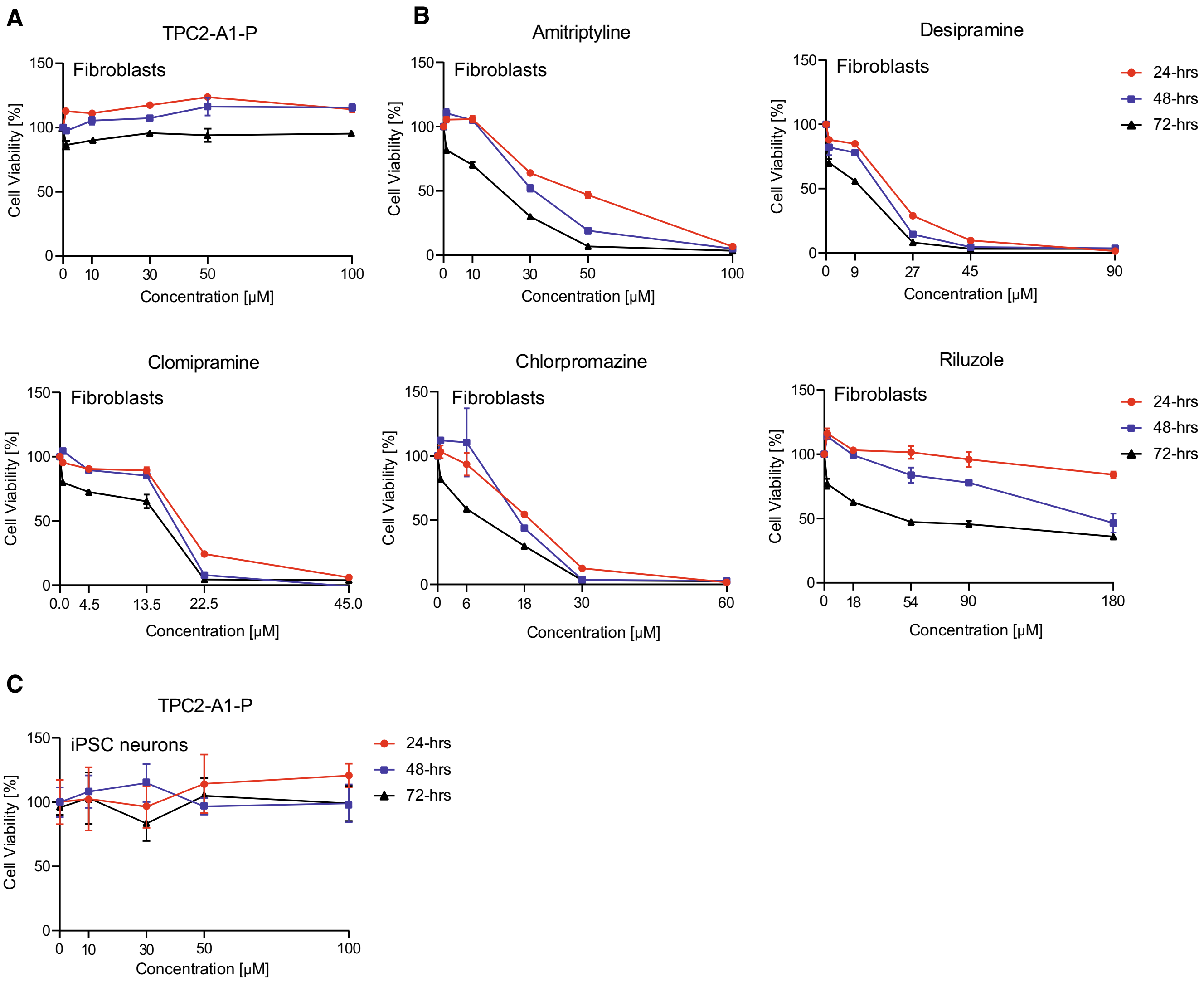

Figure EV1. Effect of TPC2-A1-P and various drugs reported to activate TPC2 on cell viability.

A–C Cell viability assay for TPC2-A1-P and other compounds reported to activate TPC2 (Zhang *et al*, 2019) on human patient fibroblasts (A, B) and iPSC-derived neurons (C). Cells were incubated for 24, 48, and 72 h with increasing compound concentrations, and cell viability was assessed with CellTiter-Blue according to the manufacturer's protocol. Data are presented as mean ± SEM. $n > 3$ for each tested condition.

© 2022 The Authors

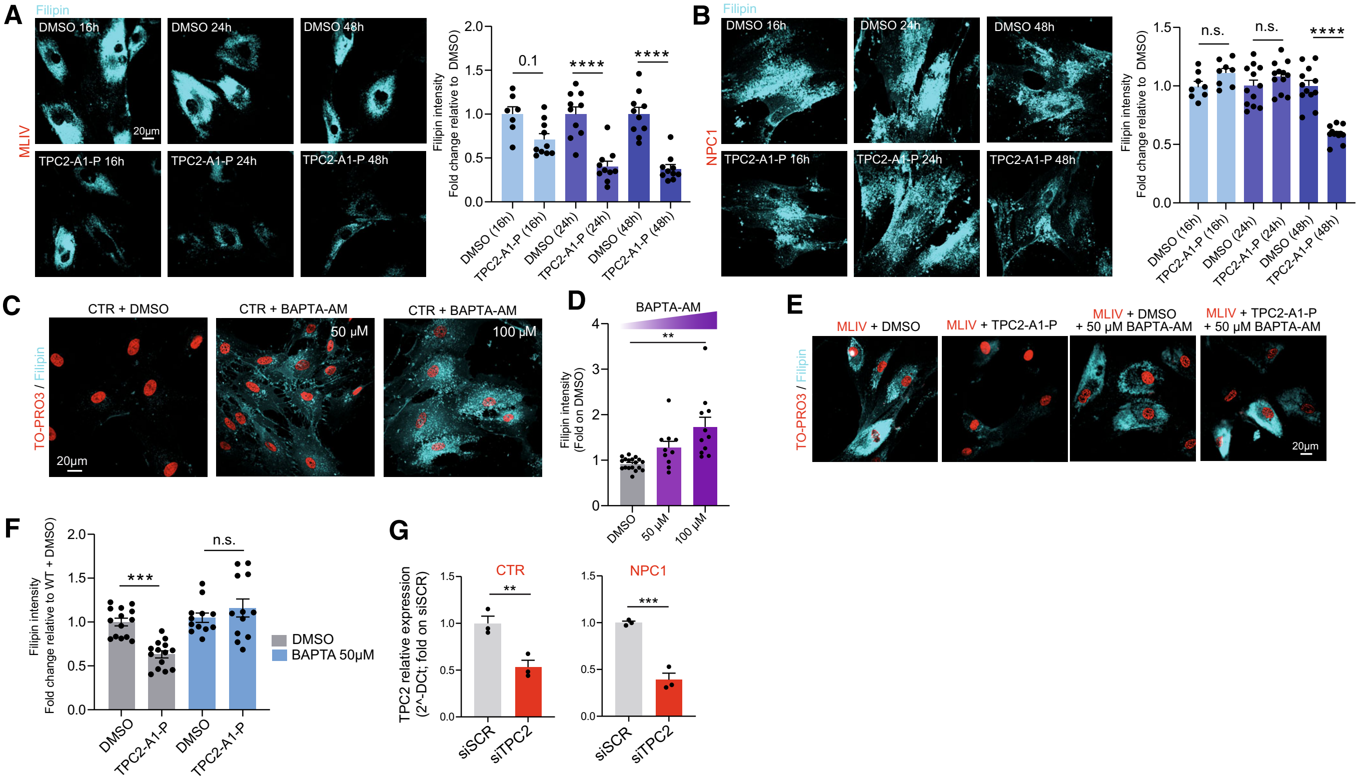

Figure EV2. Time course of filipin rescue and effect of BAPTA-AM in human fibroblasts.

A, B Time course of filipin rescue (treatment with either DMSO or 30 µM TPC2-A1-P) in human MLIV and NPC1 fibroblasts (16–48 h).

C, D Ca^{2+} chelation (BAPTA-AM) dose dependently causes cholesterol accumulation in CTR fibroblasts.

E, F Ca^{2+} chelation (BAPTA-AM) blunts the effect of TPC2-A1-P (48 h treatment) when added for the last 3 h.

G RT-qPCR showing TPCN2 knockdown efficiency in human CTR and NPC1 fibroblasts.

Data information: Shown are mean values ± SEM. $n > 3$ technical and biological replicates for each tested condition (each dot represents an imaged frame containing several cells or three independent qPCR experiments, respectively); one-way (A, B) or two-way (F) ANOVA, *post hoc* Tukey's multiple comparisons test, or two-tailed Student's *t*-test (D, G). **p-value < 0.01; ***p-value < 0.001; ****p-value < 0.0001.

Figure EV3. Colocalization of GFP-CLN3 and Batten disease-causing missense mutants with endolysosomal markers (LAMP1 for LE/LY, Rab5 for EE, and Rab11 for RE) and MitoTracker-DR. ▶

A–D Confocal images of CLN3 KO HeLa cells cotransfected with either GFP-CLN3 CTR (WT) or GFP-CLN3 missense mutant variants (as indicated) and endolysosomal markers: LAMP1-RFP, Rab5-RFP, or Rab11-DsRed. Six mutants (B), $CLN3^{S131R}$, $CLN3^{C134R}$, $CLN3^{A158P}$, $CLN3^{G165E}$, $CLN3^{L170P}$, and $CLN3^{V330I}$, appeared strongly mislocalized to the cytosol. When present in patients (usually heterozygously, alongside the more prevalent $CLN3^{\Delta 1.02kb}$ variant), these variants reportedly result in a variety of clinical phenotypes, including classic JNCL, cone-rod dystrophy, autophagic vacuolar myopathy, or retinitis pigmentosa (RP). A further six mutants (C), $CLN3^{L101P}$, $CLN3^{G189W}$, $CLN3^{I285V}$, $CLN3^{E295K}$, $CLN3^{R334C}$, and $CLN3^{Q352H}$ showed no significant difference in colocalization with LAMP1, Rab5, or Rab11 compared to CTR CLN3. For these, clinical phenotypes have not been described, incompletely characterized, or described as protracted Batten disease or RP. The remaining nine mutations (D), $CLN3^{G187A}$, $CLN3^{G189R}$, $CLN3^{G192E}$, $CLN3^{V290L}$, $CLN3^{L306H}$, $CLN3^{V330F}$, $CLN3^{R334H}$, $CLN3^{R405W}$, and $CLN3^{D416G}$ showed significantly reduced lysosomal localization (LAMP1), while retaining endosomal localization. $CLN3^{D416G}$ also showed a significant decrease in Rab11 (recycling endosome) colocalization compared to CLN3 CTR. Rab5 (early endosome) colocalization was altered in four of these nine mutants, including $CLN3^{D416G}$. Due to its consistent reduction in colocalization with all endolysosomal markers, $CLN3^{D416G}$ was chosen as a candidate for iPSC generation (with classic, more severe clinical JNCL phenotype).

E Quantification of experiments as shown in A-D. Shown are the respective Mander's correlation coefficients (MCC) for automated colocalization analysis (JACoP/Fiji) of GFP-CLN3 CTR and missense mutants with LAMP1-RFP, Rab5-RFP, Rab11-DsRed, or MitoTracker-DR (negative control).

Data information: Data are presented as mean ± SD. $n > 3$ technical and biological replicates for each tested condition; one-way ANOVA Dunnett's multiple comparisons test. *p-value < 0.1; **p-value < 0.01; ***p-value < 0.001; ****p-value < 0.0001.

© 2022 The Authors

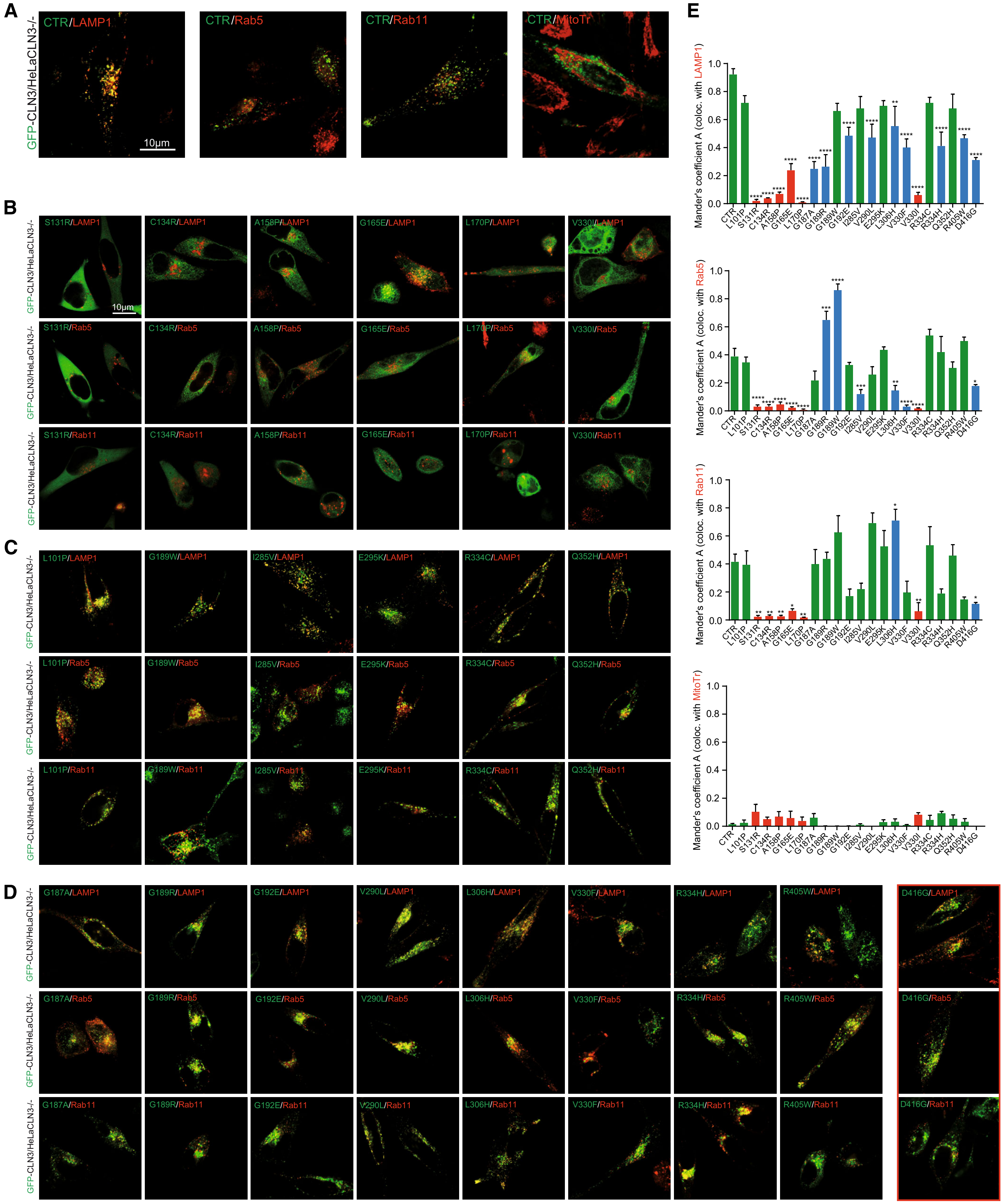

Figure EV3.

 © 2022 The Authors

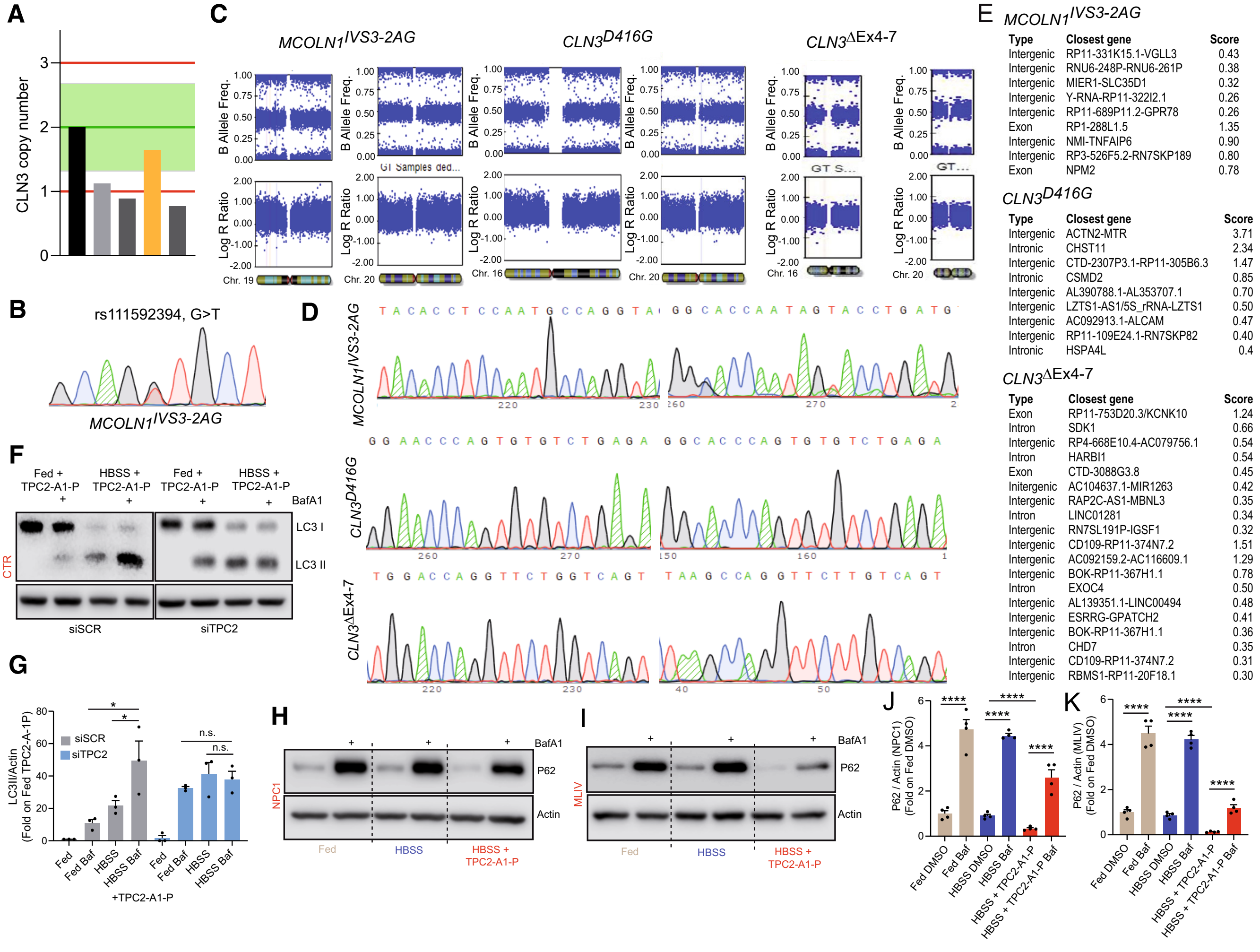

Figure EV4. Quality control of novel MLIV and CLN3 iPSC lines and autophagy (LC3 and P62) experiments.

- A Four CLN3^{D416G} iPSC clones were screened for locus copy numbers by qgPCR in comparison to the unedited parent line (black bar) to rule out undesired on-target editing. The clone showing two CLN3 copies was selected (yellow bar).
- B A heterozygous, silent SNP was found alongside the MLIV$^{IVS3-2A>G}$ edit, confirming the presence of both edited alleles and ruling out large indels due to on-target effects.
- C Molecular karyotyping did not reveal any detectable aberrations in the selected cell lines at the targeted locus or chromosome 20, which is frequently altered in edited iPSCs.
- D The most likely off-target sites of the gRNAs used for each edit were predicted by CFD and MIT algorithms and sequenced, revealing no off-target editing in the selected CLN3 and MLIV clones. The most likely off-target sites for each clone are depicted.
- E Tabular summary of sequenced off-target sites.
- F, G Effect of HBSS + TPC2-A1-P on LC3 in siSCR or siTPC2--treated human CTR fibroblasts.
- H–K Effect of TPC2-A1-P on P62 accumulation with and without bafilomycin A1 treatment in human NPC1 and MLIV fibroblasts.

Data information: Shown are mean values ± SEM. $n > 3$ technical and biological replicates for each tested condition (each dot in (F), (H), and (I) represent three independent western blot experiments, respectively); two-tailed Student's *t*-test (G, J, K). *$p < 0.05$; ****p-value < 0.0001.

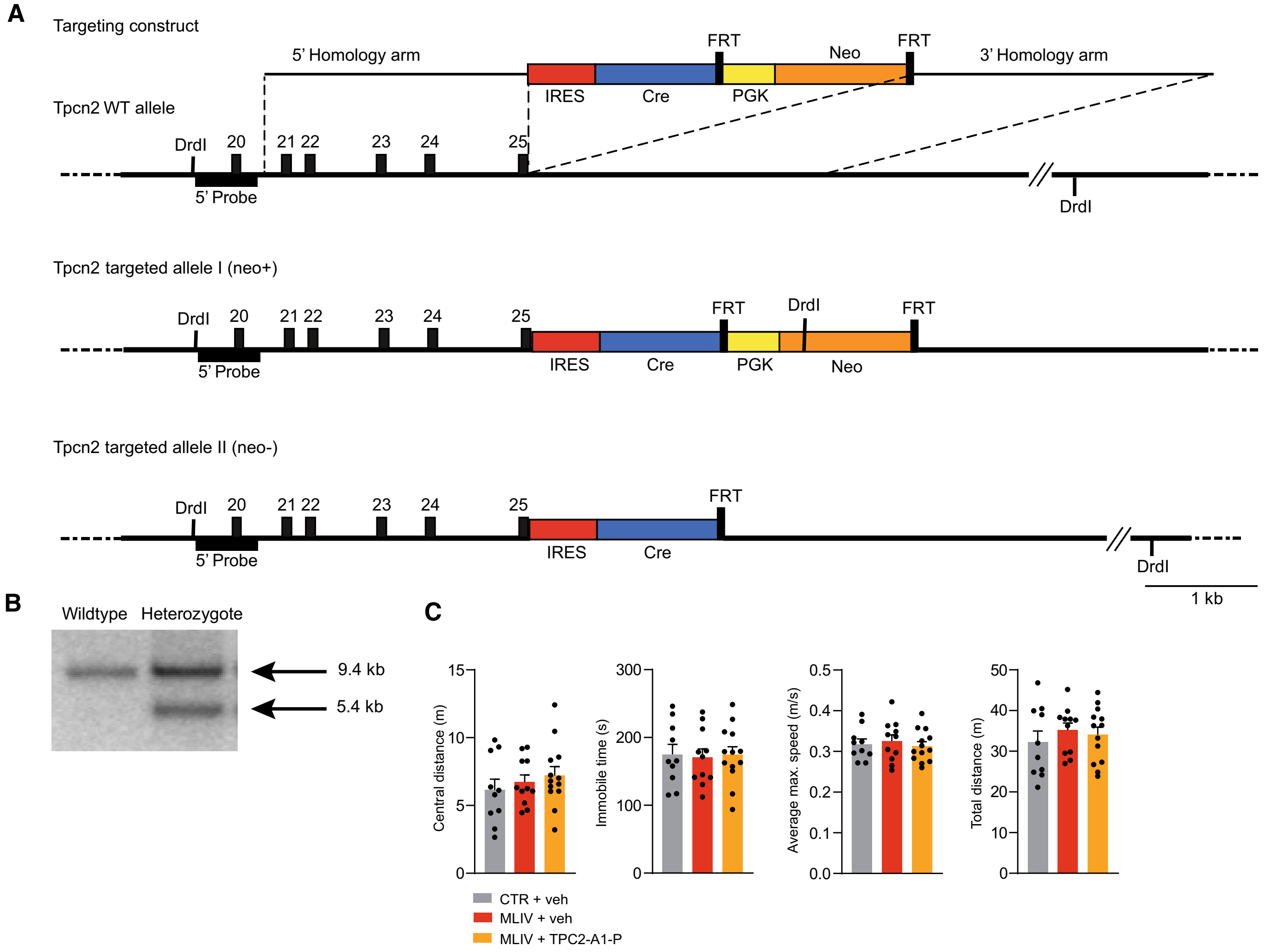

Figure EV5. Targeting strategy for TPC2 reporter mouse model and supplementary behavioral data (MLIV mouse model).

A Targeting strategy used to express Cre recombinase under the control of the Tpcn2 promoter. The targeting vector contains an IRES-Cre-FRT-PGK-NEO-FRT cassette in which a phosphoglycerate kinase promoter drives neomycin resistance (Pgk-neo). This cassette is incorporated by homologous recombination in embryonic stem cells subsequent to the stop codon in exon 25.

B Southern blot of embryonic stem cell DNA cut with DrdI, demonstrating correct targeting of the Tpcn2-IRES-Cre knock-in allele.

C Results of the open-field test using MLIV mice treated with vehicle or TPC2-A1-P, respectively, compared to vehicle-treated WT littermates (CTR). No differences between CTR and MLIV mice were observed.

© 2022 The Authors
