# Supplementary material for: TPC2 rescues lysosomal storage in mucolipidosis type IV, Niemann–Pick type C1, and Batten disease
Source: EMBO Mol Med. 2022 Aug 5;14(9):e15377. doi: 10.15252/emmm.202115377 (PMC9449600; doi:10.15252/emmm.202115377)
Supplement: Supplementary file 3 [file EMMM-14-e15377-s003.pdf]

EMBO Molecular Medicine

TPC2 rescues lysosomal storage in mucolipidosis type IV, Niemann-Pick type C1 and Batten disease

Anna Scotto Rosato, Einar Krogsaeter, Dawid Jaslan, Carla Abrahamian, Sandro Montefusco, Chiara Soldati, Barbara Spix, Mariateresa Pizzo, Giuseppina Grieco, Julia Böck, Amanda Wyatt, Daniela Wünkhaus, Marcel Passon, Marc Stieglitz, Marco Keller, Guido Hermey, Sandra Markmann, Doris Gruber-Schoffnegger, Susan Cotman, Ludger Johannes, Dennis Crusius, Ulrich Boehm, Christian Wahl-Schott, Martin Biel, Franz Bracher, Elvira De Leonibus, Elena Polishchuk, Diego Medina, Dominik Paquet, and Christian Grimm
DOI: 10.15252/emmm.202115377

Corresponding authors: Christian Grimm (christian.grimm@med.uni-muenchen.de) , Diego Medina (medina@tigem.it)

Review Timeline:

Submission Date:	3rd Nov 21
Editorial Decision:	11th Nov 21
Appeal Received:	12th Nov 21
Editor's Correspondence:	16th Nov 21
Authors' Correspondence:	11th Mar 22
Editorial Decision:	11th Apr 22
Revision Received:	4th Jul 22
Editorial Decision:	12th Jul 22
Revision Received:	14th Jul 22
Accepted:	15th Jul 22

Editor: Zeljko Durdevic

Transaction Report:

(Note: With the exception of the correction of typographical or spelling errors that could be a source of ambiguity, letters and reports are not edited. Depending on transfer agreements, referee reports obtained elsewhere may or may not be included in this compilation. Referee reports are anonymous unless the Referee chooses to sign their reports.)

11th Nov 2021

Decision on your manuscript EMM-2021-15377

Dear Prof. Grimm,

Thank you for submitting your manuscript to EMBO Molecular Medicine. I have now had a chance to read your manuscript carefully and to discuss it with the other members of our editorial team. In addition, I have also sought external advice on the study from an expert in the field. I regret to say that we find the article not well suited for publication in EMBO Molecular Medicine.

Your study reports in vitro and in vivo effects of previously published two-pore channel 2 (TPC2) agonists on different types of lysosomal storage diseases (LSDs). Your work suggests that TPC2 might be a promising novel target for the treatment of various LSDs. We do recognize the potential interest of these observations; however, we are not persuaded that they provide the sort of translational relevance we would expect in an EMBO Molecular Medicine article. In particular the in vivo effects of the TPC2 agonism would require further investigation to allow the statement that the TPC2 agonist has a therapeutic effect. As mentioned above, we have also sought further advice on the study, but I am afraid our advisor shared our opinion that translational aspect of the study is not sufficiently developed. Given these considerations, we find the manuscript not well suited for publication in EMBO Molecular Medicine.

I am sorry that I can't be more positive on this occasion. Please rest assured that this is not a judgment of the quality or interest of your work, but a decision based on appropriateness for EMBO Molecular Medicine.

Yours sincerely,

Zeljko Durdevic

Zeljko Durdevic
Editor
EMBO Molecular Medicine

As a service to authors, EMBO provides authors with the possibility to transfer a manuscript that one journal cannot offer to publish to another EMBO publication. The full manuscript and if applicable, reviewers reports are automatically sent to the receiving journal to allow for fast handling and a prompt decision on your manuscript. For more details of this service, and to transfer your manuscript to another EMBO title please click on Link Not Available

Please do not share this URL as it will give anyone who clicks it access to your account.

LUDWIG-MAXIMILIANS-UNIVERSITÄT MÜNCHEN

LMU MÜNCHEN, MEDIZINISCHE FAKULTÄT
WALTHER-STRAUB-INSTITUT FÜR PHARMAKOLOGIE

To:

Dr. Zeljko Durdevic
Editor EMBO Mol. Med.

Prof. Dr. Dr. Christian M. Grimm

Telefon +49 (0)89 2180-73811
Telefax +49 (0)89 2180-73817
Mobile +49 (0)157 34011173

christian.grimm@med.uni-muenchen.de

Walther-Straub-Institut für Pharmakologie und Toxikologie
LMU München
Nussbaumstrasse 26, A0.062
80336 Munich, Germany

München, 11.11.21

EMM-2021-15377

Dear Dr. Durdevic,

Thank you very much for carefully reading our MS EMM-2021-15377. In your Decision Letter you write that you "*are not persuaded that {our data} provide the sort of translational relevance {you} would expect in an EMBO Molecular Medicine article. In particular the in vivo effects of the TPC2 agonism would require further investigation to allow the statement that the TPC2 agonist has a therapeutic effect.*"

We are very surprised by this statement and the decision for the following reasons:

1) You do not specifically explain what kind of in vivo data you would expect beyond the ones shown. We would kindly ask to specify this in order for us to better understand the decision you made.
2) You do not acknowledge at all that in vivo efficacy very much depends on the pharmacokinetics and pharmacodynamics of the applied small molecule besides many other features and parameters. Infact, we are extremely lucky to find in vivo efficacy to the extent as currently shown. Our intent was to show positive proof of concept, which we did by using different (!) models, human patient fibroblasts, human iPSC derived cortical neurons and yes also the MLIV mouse model.

3) You do not at all mention or seemingly appreciate the enormous effort we took to develop IPSC models for MLIV and Batten disease. Researchers around the globe are impatiently waiting for such models to work with. We invested several years in developing these models of the human diseases and therefore even the more do not understand why you emphasize efficacy in the mouse (!) model so much? We show efficacy in human (!) neurons.
4) If any in vivo model was really relevant here then it would be testing of the compound in human patients, but you will appreciate that this cannot be done as part of an EMM study.
5) We are concerned with the custom of asking one (!) "expert" in the field to drive decisions on whether a paper should be sent out for peer review or not. What if by any chance this "expert" is biased or a direct competitor? An opinion by one single expert seems very problematic in our view.

In light of this we would ask you to please reconsider your decision and to either outline what kind of in vivo data you would expect to see in order to find our work appropriate for EMM or to send the MS out for peer-review to get a chance for a balanced and fair judgement of our work.

Mit freundlichen Grüssen,

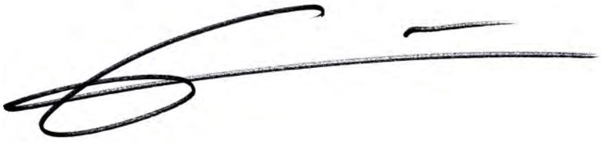

Prof. Dr. Dr. C. M. Grimm

16th Nov 2021

Dear Prof. Grimm,

Thank you for your response to the editorial decision on your manuscript entitled "TPC2 rescues lysosomal storage in mucolipidosis type IV, Niemann-Pick type C1 and Batten disease". I have carefully examined the arguments provided in your letter and discussed them with other members of our editorial team. I am pleased to inform you that we decided to re-consider our initial decision and invite resubmission of your manuscript with a commitment to send it for peer review if you obtain data that would strengthen the in vivo aspect of the study. As discussed with you the manuscript should include in vivo experiments with increased number of animals per group, behavioural test and/or analysis of additional tissues of the MLIV mouse model and CLN3 knockout iPSCs.

I look forward to receiving your manuscript.

Yours sincerely,

Zeljko Durdevic

Zeljko Durdevic
Editor
EMBO Molecular Medicine

LMU LUDWIG-MAXIMILIANS-UNIVERSITÄT MÜNCHEN

LMU MÜNCHEN, MEDIZINISCHE FAKULTÄT
WALTHER-STRAUB-INSTITUT FÜR PHARMAKOLOGIE UND TOXIKOLOGIE

Prof. Dr. Dr. Christian M. Grimm

Telefon +49 (0)89 2180-73811
Telefax +49 (0)89 2180-73817
Mobile +49 (0)157 34011173

christian.grimm@med.uni-muenchen.de

Walther-Straub-Institut für Pharmakologie und Toxikologie
LMU München
Nussbaumstrasse 26, A0.062
80336 Munich, Germany

Zeljko Durdevic
Editor
EMBO Molecular Medicine

zeljko.durdevic@embo.org

Ihr Zeichen, Ihre Nachricht vom Unser Zeichen

München, 11.03.2022

Dear Dr. Durdevic,

Thank you very much once again for the very constructive zoom-meeting on 16.11.21. In this meeting we had discussed our MS entitled *"**TPC2 rescues lysosomal storage in mucolipidosis type IV, Niemann-Pick type C1 and Batten disease**"*, originally submitted to EMM on 03.11.21.
In agreement with the editors of EMM, you offered us to hand in a revised MS for further consideration if we were able to provide more n numbers for the in-vivo experiments and also potentially additional in-vivo experiments. We have now worked towards this goal (more n numbers, more experiments). In addition we have added further material and data to strengthen the MS, such as a newly generated Batten disease knockout hiPSC line (in addition to the mucolipidosis type IV iPSC line and a CLN3 point mutant line (D416G) for Batten disease, which had already been part of the previous MS version).

In detail, we added the following material and new data sets to the MS:

1) Knockout CLN3$^{\Delta Ex4-7}$ hiPSC line and experiments with neurons derived from these in analogy to existing exp. with the other two lines (See new Fig. 4 and 5).
2) We have increased the n numbers of WT and MLIV mice in our in-vivo analysis to complement the existing data and added new data. We thus provide now new and more complete datasets to characterize the in-vivo efficacy of TPC2-A1-P as agreed (See new Fig. 9).
3) We now show endolysosomal patch clamp data where we electrophysiologically characterized the MLIV iPSC derived neurons and the TRPML1 currents compared to WT (See new Fig. 4F).
4) We have also added a new Fig.10, depicting a cartoon which summarizes our findings schematically.

Generally, we would like to emphasize that MLIV knockout mice are notoriously difficult to breed. Nevertheless, we were able to obtain more mice and to do more injections of TPC2-A1-P with WT and MLIV mice, and thus could strengthen our in-vivo analyses (Point 2).

Currently, we are still continuing to inject additional mice (which were born later) and to breed more mice for behavioral tests (e.g. Rotarod). However, this will likely take another couple of months.
Given that since the initial submission several months have already passed and competitors are submitting similar papers at the moment, we would like to ask if the editors of EMM could discuss the possibility to send out our MS for peer-review at this point, with the newly performed exp. and additional mouse numbers/in-vivo data as per our agreement, which we have fulfilled accordingly.
Nevertheless, we will be continuing with our breeding scheme to perform additional in-vivo studies for a potential revision. We very much hope this would be a fair option and acceptable solution.

Many thanks again for considering our MS for EMM. If we may answer any questions related to this submission in the meantime, please do not hesitate to contact us at the numbers or email listed above.

All authors are familiar with the present manuscript and concur in its submission. This work has not previously appeared in print, and is not under editorial consideration elsewhere.
Please direct all editorial correspondence to me at the following address:

Prof. Dr. Dr. Christian Michael Grimm
Department of Pharmacology and Toxicology, Medical Faculty
Ludwig-Maximilians-University (LMU) Munich
Nussbaumstr. 26, 80336 Munich, Germany
Tel.: +49(0)89/2180-73811
email: christian.grimm@med.uni-muenchen.de

Sincerely,

Prof. Christian Grimm

11th Apr 2022

Dear Prof. Grimm,

Thank you for the submission of your manuscript to EMBO Molecular Medicine. We have now received feedback from the three reviewers who agreed to evaluate your manuscript. As you will see from the reports below, the referees acknowledge the interest of the study but also raise serious concerns that should be addressed in a major revision. Particular attention should be given to re-structuring of the manuscript, improving presentation of the results (i.e. some panels and in some cases whole figures could be moved to Appendix, please check our Author Guidelines: https://www.embopress.org/page/journal/17574684/authorguide#expandedview) and re-writing of the manuscript in more focused and clear manner. Please also move M&M from the Appendix to main manuscript M&M section.

Further consideration of a revision that addresses reviewers' concerns in full will entail a second round of review. EMBO Molecular Medicine encourages a single round of revision only and therefore, acceptance or rejection of the manuscript will depend on the completeness of your responses included in the next, final version of the manuscript. For this reason, and to save you from any frustrations in the end, I would strongly advise against returning an incomplete revision.

We would welcome the submission of a revised version within three months for further consideration. Please let us know if you require longer to complete the revision.

Please use this link to login to the manuscript system and submit your revision: Link Not Available

I look forward to receiving your revised manuscript.

Yours sincerely,

Zeljko Durdevic

Zeljko Durdevic
Editor
EMBO Molecular Medicine

***** Reviewer's comments *****

Referee #1 (Remarks for Author):

The present ms explores an emerging approach for lysosomal storage disorders based on a new molecule that activates the two pore channel TPCN2. The work follows up on ground-breaking results published earlier by the same group (Gerndt et al., 2020 Elife), where they showed that the ion selectivity of the TPCN2 channel depends on the endogenous activator. Notably, the group identified two distinct small-molecule agonists that mimick the endogenous activators and thereby induce distinct functions of the channel in its natural habitat, the endosomal-lysosomal system. Here, the authors claim that one of these agonists, named TPC2-A1-P, shows beneficial effects in a range of cellular models of different lysosomal storage disorders, namely mucolipidosis IV, which is caused by genetic defects in a similar channel named TRPML1/MCOLN1. The authors have amassed an impressive amount of in vitro and in vivo results, and they provide a bit of in vivo data showing effects of the agonist on a mouse model of mucolipidosis type IV. The results could be of interest, but the ms has numerous severe flaws that preclude its publication. The following points are of concern:

Major points

- The team's above-mentioned discovery of two distinct small molecule activators (TPC2-A1-P and -N; Gerndt et al., 2020 Elife, see also Gerndt et al., 2020 FEBS) would have provided a unique chance to explore which TPCN2 mode of function (ion selectivity) is required to mitigate disease-specific pathologic changes in the different models (or at least an informed selection of those). Unfortunately, it is not explained why the authors have chosen to test only the effects of TPC2-A1-P.

- In all relevant figure panels, the authors must state what is represented by individual symbols in the graphs: cells/animals or means of preparations etc. The author must also state how many cells/preparations/micrographs etc., i.e. how many biological/technical replicates were used for the statistical analyses. The statement on pg. 13, para 6 ("was conducted on datasets where n > 3") is not sufficient, last not least because it is unclear what "n" represents (preps, animals, cells etc.).

- Pg. 4, para 3, Fig. 1A: The two graphs in this panel show exactly the same data except for distinct control (CTR) lines. Duplicate presentation of data is inacceptable.

- The authors' intention to test the effects of their drug candidate in different cell-based disease models is laudable and could provide important insight. Principally, one would assume that such drug tests are performed with the most suitable assay measuring pathologic accumulation of a disease-specific molecule(s). In the case of SMPD1- and NPC1-deficiency, for example, this would probably be sphingomyelin and cholesterol, respectively, in the case of mucolipidosis IV, the lactosylceramide-based assay can be a valid choice. However, throughout the Results part the authors use a bewildering range of assays without much justification/explanation of their choices and a clear lack of focus. Moreover, the results are shown in an inconsistent and eclectic manner. This must be completely revised including a redesign of figures. Examples to illustrate these points:

(1) The authors state on pg. 4, para 3 that they "assessed the effect of TPC2 activation in different LSD fibroblasts". However, it remains unclear, which fibroblasts/disease models were used and why and it is unclear why the results of these tests are not shown except for MLIV. This is inacceptable. It is important to see how the TPC2 agonist affects the different models with each given assay. In the following sentence, the authors describe results from a distinct approach (overexpression of TPC2 and TRPML1 in three disease contexts, MLIV, NPC1, NPA). Again, there is no explanation why this approach was selected and why it is applied to three diseases. In the corresponding panels of Fig. 1, micrographs for MLIV and NPA are shown, whereas quantitative data are shown for three diseases. Again, it is unclear, why incomplete datasets are presented.
(2) A similarly inconsistent presentation of results can be found on pg. 5-6 and Fig. 2 with respect to the filipin assay and

ultrastructure. Again, the authors claim that effects of the agonist were tested in different patient fibroblasts, but it is unclear in which ones, and the outcome is only shown for selected disease models, in the case of filipin staining for MLIV and NPC1, in the case of ultrastructure JNCL, MLIV and NPC1 for vehicle controls, and NPC1 for agonist effects. Here, ML-SA1 data are presented without explanation: why was the agonist used in this assay, but not in the other assays presented in preceding pages/figures?

- Pgs. 4-6: Bioassays should be performed using different drug concentrations and durations of treatment to determine the full range of drug effects. The authors tested only a single concentration of their drug candidate (e.g. 30 µM of TPC2-A1-P; except for EV5) and they used treatment durations ranging from 2h (for the lactosyl-ceramide assay), 180 min (= 3h; autophagy assay) to 48-72h (lysotracker filipin staining) depending on the assay. The choice of these paradigms is not explained and one wonders about their validity. The reported EC50 values in Fluo-4 calcium experiments were in the 1-10µM range, whereas the EC50 values obtained by electrophysiological recordings were 10-fold lower. The authors' assumption that the use of a single concentration is sufficient to explore the effects of their drug in different lysosomal diseases with distinct mechanisms is not justified.

- Pg. 4-5, Fig. 1D-F, Fig. 2E-G: The authors tested drug effects on fibroblasts overexpressing wildtype versions of TPC2 or TRPML1 channels using different assays (LacCer accumulation, filipin). However, the authors do not show the effects of channel expression with vehicle treatment. The outcome of these standard controls must be included. Moreover, a description of the plasmids used to transfect cells must be given in the relevant sections of Material/Methods (pg. 11). On pg. 4, para 3, the authors state that MLIV cells respond "strongly" to TPC2/TPC2-A1-P treatment (see Fig. 1D). This is only partially true, it appears that only a subset of cells (or preparations? See comment below) is affected. Does this depend on the level of channel expression?

- Pg. 5, para 1, Fig. 1G; Fig. 2H-I: It is unclear, why the authors show effects of BAPTA on LacCer and filipin distribution in "wildtype" fibroblasts. With respect to LacCer, this experiment cannot address the question why ion channel overexpression and activation in NPA disease fibroblasts failed to affect LacCer accumulation. It is further unclear to which effects authors refer to in the sentence "Hypothesizing that the observed effects were due to lysosomal Ca2+ release". With respect to cholesterol, the reason why these experiments were performed is not indicated. Since the relation of these experiments to the specific goals of the present study is unclear, they should probably removed.

- Pg. 5, para 1: The statement "Altered cellular cholesterol homeostasis is rather common in LSDs" is imprecise and should be removed. There is a large number of LSDs where cholesterol distribution is unaffected. The authors mention at least one, JNCL.

- Pg. 5, para 1, EV1: The results shown in expanded view 1 are interesting, but only partially related to the present study. Moreover, they are introduced and explained in an insufficient manner. The fibroblasts are incompletely characterized, different agonists were used, and effects of TPC1-A1-P on IV curves are not shown.

- Pg. 5, para 1, EV2: The author show yet another series of experiments (siRNA-mediated silencing of TPC2) rather en passant without much explanation. Evidently, these results should be shown in a main figure 2, as target validation in vitro is an important element. On the other hand, the results are incomplete, as the validation is only shown with respect to NPC1 and filipin, not for example with respect to LacCer accumulation for example in MLIV fibroblasts related to experiments presented in Fig. 1. No explanation is given. In EV2, the y axes label reads "Filipin-cholesterol load" but it is unclear, how this parameter was measured. In EV2B, values are normalized to fibroblasts treated with scrambled siRNA and with DMSO. However, is this possible, because one would expect that the cells treated with siTPC2 show an increased level of cholesterol load compared to siSCR, which then is reduced or not by TPC2-A1-P. In EV2C, the abbreviation in the label of the y axes is not explained.

- Pg. 6, para 1; pg. 8, para 1; Fig. 2J-L; Fig. 5H,I: Results obtained by electron microscopy are presented, but the ms lacks a description of the experimental procedures used and of the way quantitative data were obtained. This information must be provided.

- Pg. 7, para 2; Fig. 4F,G: The authors show effects of their favorite agonists on channel IV curves and current densities, but these findings are not mentioned in the Results.

- Pg. 7, para 2; Fig. 5A: It is unclear what is represented by the measurements shown in Panel A. Are these mean values obtained from n = ? independent preparations?

- Pg. 7, para 2: The authors state "functionally we assessed these neurons by... ": It is doubtful whether a cathespin B activity assay can be used to analyse neuronal function. This statement should be revised and the following findings should be introduced properly.

- Pg. 8, para 2: The authors propose to study lysosomal exocytosis as "potential rescue mechanism", which is a valid idea that has been explored previously. However, it remains unclear which "observed rescue effects" they refer to. Moreover, it is unclear why these data obtained with cultured fibroblasts are shown here and not in the preceding paragraphs where the "observed rescue effects" were presented ?

- Pg. 8, para 2: The validity of macrophages as "an independent cellular model" cannot be estimated, because the authors have neither established the presence of the pathologic changes (e.g. accumulation of LacCer or cholesterol, disturbance of autofluorescence etc.) in these cells nor did they show that the agonists reduce these changes at the first place.

- Pg. 8, para 2: The authors propose autophagy as potential rescue mechanism. This appears a bit strange given that autophagy itself is disturbed in LSD diseases: How can a pathway that is perturbed serve as rescue mechanism? Again, the authors test this idea in selected fibroblasts of two diseases without explanation why in these models and not in others.

- Pg. 8, para 2/EV5: The authors' claim that "In TPC2 knockout fibroblasts, we observed no effect with TPC2-A1-P" is inaccurate and not supported by the data. The experiments were performed in a completely different manner than the ones shown in Fig. 7A-I. First, no fed-starved conditions were examined. Second, for unknown reasons, the authors used mouse embryonic fibroblasts (if this is what MEF stands for). Third, the experiments were performed without bafilomycin control, which is inacceptable for this kind of assays. Fourth, the authors employed a much lower agonist concentration (1-5µM EV5 compared to 30 µM Fig. 7). Moreover, the y-axis label is incorrect, as the values were obviously normalized, but it is unclear to what. The meaning of the results and their relation to experiments performed on "autophagic flux" are unclear.

- Pg. 8, para 2; Fig. 7: iPSC-derived neurons were maintained under fed and starved conditions using two distinct media (Neurobasal versus DMEM, as indicated in the legend). Given their distinct compositions, can the authors exclude that their protocol induces cellular changes other than just those due to a fed-starved transition?

- Pg. 8, para 2; Fig. 7: It is unclear based on which results shown in the figure the authors claim that "TPC2-A1-P recovered impaired autophagic flux in NPC1 and MLIV". In MLIV fibroblasts, the basal changes induced by starvation and by bafilomycin (E, F) and the effect of the agonists are much smaller compared to those in WT and NPC1 fibroblasts (compare scales panel B, E, H). The measurements of P62 levels were performed without the bafilomycin control, and therefore it is unclear to which degree the levels of P62 under the distinct condition depend on autophagic degradation.

- Fig. 7: Graphs in panels C, F & I show the same data as presented in panels B, E & H. Duplicate presentation of results is inacceptable. In addition, it is not clear what the y axes show. Are the values normalized to some control conditions: the values seem to equal 1 in DMSO+Fed condition. If this is the case, the titles of the y axes must be corrected.

- Fig. 7K: The y axis label states "Ctr fed dmso" but it is unclear what CTR refers to?! Wt? In this case, the first value is probably incorrect, as it should be 1.

- Pg. 8-9: The generation of a TPC2-IRES-Cre line is a strength of the study as this tool will be of interest for future investigations. The authors should probably note in this part of the Results that the outcome of recombination and thus the type of cells labeled and their percentages in distinct brain regions depends on the Cre-dependent reporter line used.

- Pg. 9-10: The in vivo experiments are an important part of the study, but the methods used to test effects of the TPC2-A1-P on MLIV mice are not described in the Material/Methods section. Notably, a "statement in the Materials and Methods identifying the institutional and/or licensing committee approving the experiments" as demanded by EMBO (and any other journal) is missing both for the pharmacokinetic study on BL6 mice and for the TCP2 study on MLIV mice.

- Pg. 9-10: With respect to the tests in MLIV mice it is key to know which vehicle has been used (DMSO-PEG400 as stated for the pharmacokinetic analysis) to deliver the agonist and whether the same has been used as vehicle control (DMSO or DMSO-PEG400). DMSO alone without PEG400 would be inacceptable.

- Pg. 9-10: In the same context, it is key to know whether littermates have been used as wildtype controls. Evidently, littermates represent the only valid control for this kind of studies.

- Pg. 9-10; Fig. 9: The authors indicate the densities of IBA1- and GFAP-positive cells in different brain regions of MLIV and wildtpye animals treated with DMSO, but data from agonist-treated mice are only shown for one brain region (arbor vitae). This is inacceptable. Full datasets must be shown to judge the robustness of the effect across brain areas. Panel E repeats data shown in D.

Other points
- The ms suffers from an inconsistent use of terms and abbreviations in figures and text. This must be revised. In the following, a few examples are mentioned: (1) Pg. 2, para 1 and throughout ms: The authors should use consistently the official symbol TPCN2 for the two-pore segment channel. (2) Pg. 4, para 3 and throughout the ms. The authors should use consistently the official abbreviation for TRPML1, which is MCOLN1. This includes also the designation of human gene mutations or protein variants. The parallel use of both terms is particularly confusing on pg. 7 and Fig. 4. (3) Throughout the ms and figures the authors use CTRL, CTR or wildtype. They should use consistently one term. With respect to fibroblasts from healthy donors, the term "wildtype" may not be appropriate. (4) The abbreviation "HF" is used in some figures (e.g. Fig. 3, Expanded View) and not in others. This must be corrected. (5) On pg. 6 and Fig. 3 the terms JNCL and CNL3 are used, respectively. This should be

corrected.

- Pg. 3, para 1: For clarity the authors should add to the more historic term Niemann-Pick type A the correct nosology "infantile neurovisceral form of acid sphingomyelinase (SMPD1) deficiency" and use this subsequently in the text.

- Pg. 11 para 3 & Fig. 1A: The authors should explain whether analysis of colocalization was performed on selected regions of interest containing cells or on the entire image. Evidently, the former is preferred.

- Pg. 12 (and elsewhere): Statements such as "immunofluorescence targeting LAMP1 protein is performed overnight" or "Autofluorescence mean intensity in the 488nm channel and 405 channel in LAMP1" are lab jargon and must be replaced by precise statements. The excitation and emission wavelengths to measure autofluorescence etc. must be stated clearly.

- Pg. 13, para 6: Replace "to-tailed" by "two-tailed".

- Pg. 19, para 1: The statement "which showed the most significant assay window" is unclear and should be replaced/removed.

- Figures: The authors should consider to redesign their graphs and figures to render them more intelligible. For example, in Fig. 1, panel C shows two types of measurements: are the ranges of y axes really similar: density of puncta versus Mander's coefficient? Other example: panels E and F show micrographs whereas the quantitative data are presented already in D. Thus, the link between the panels is not obvious.

- Fig. 1B: The authors should consider to rearrange the panel and place MLIV treated with DMSO and with drug side-by-side.

- Fig. 1C: Here, and elsewhere, the term "spots" should probably be replaced by "puncta".

- Fig. 3, legend: The description of panels C and D is incorrect and must be revised. X axis designations (t0) and the y axis title are undefined.

Referee #2 (Comments on Novelty/Model System for Author):

The authors present interesting findings on the effects of activation of TPC2 on the cellular phenotypes of a set of lysosomal storage disorders. Extensive data are provided demonstrating attenuation of aberrant lactosyl ceramide trafficking and cholesterol accumulation along with enhanced lysosome exocytosis among other changes associated with application of the TPC2 activator TPC2-A1-P. Pharmacokinetic studies and effects on some aspects of a mouse model of MLIV are also presented. The study builds on previous work by this group on TPC2 activation and work on TRPML1 and the effect of its activation on lysosomal storage disorder cellular pathophysiology. The effects of TPC2-A1-P on the is marked and reflects a potential for significant clinical impact on a subset of lysosomal storage disorders.

Referee #2 (Remarks for Author):

There are a few minor issues with the manuscript.
- The authors make the statement "TPC2-A1-P treatment significantly decreased CtsB activity in iPSC-derived MLIV neurons, supporting its therapeutic relevance in MLIV". To this reviewer this is an overstatement - decreasing CtsB in a cell model does not support therapeutic relevance.
- The authors use "wild-type" for human cells derived from non-diseased control subjects - "control" would be more appropriate.
- There are is no method description for the EM and importantly for the analysis of figures - importantly, how many samples/grids were used for each experiment and were those collecting/analyzing images blinded to sample treatment?
- While TPC2-A1-P clearly acts on TPC2, confirming that at least one of the effects of TPC2-A1-P on one of the lysosomal storage disorder models is dependent up on TPC2 would help exclude an off target effect. It would also be interesting to test whether BAPTA blunts the effect of TPC2-A1-P.
- A discussion about mechanisms to explain the differential effects on the different LSDs would be useful - e.g. why is Niemann Pick A lactosyl ceramide unaffected? Understanding the differential effects on different diseases could better the understanding of mechanism.

Referee #3 (Comments on Novelty/Model System for Author):

Rosato et al explore the effects of small molecule activation of the endolysosomal two-pore channel 2 (TPC2) in diverse models of lysosomal disease, including patient fibroblasts and iPSC-derived neurons harboring mutations in several disease-causing genes as well as in MLIV mice. In aggregate, their data provide evidence of therapeutic benefit from activating TPC2 in a number of disease models by clearing storage material and promoting lysosomal exocytosis and autophagy. Initial data from MLIV mice also suggest a beneficial effect. These finding are reminiscent of the beneficial effects that have been reported for activation of TRPML1 and identify an additional potential target for therapeutic intervention. While these observations are of

potential interest, several issues need to be addressed:

Scientific rigor requires attention. For the quantification of imaging studies in multiple figures involving cells and mice, it is unclear in graphs what each dot represents. It is not clearly stated how many experimental replicates were performed, how many cells per replicate were analyzed, or which statistical test was used to assess significance. F statistics should be included for ANOVA.

The specificity of effects on TPC2-A1-P on cellular phenotypes (cholesterol, LacCer, lamellar structures) should be confirmed in TPC2 knockdown or null cells. Additionally, time- and dose-dependence should be shown. Therapeutic effects should be shown to occur without triggering cell death; this is particularly important for studies using iNeurons.

The animal studies reported in Fig 9 are under-developed and show only partial rescue of downstream components of the pathogenic cascade.
-Is there evidence that drug treatment alters lipid storage and behavioral phenotypes?
-A marker of target engagement in the CNS should be provided to confirm that rescue is not mediated by off target effects of drug administration.
-Changes to autophagic markers should be corroborated by western blot.
-Is analysis of the cerebellum from the same lobule? Many lysosomal models show a gradient of pathology across folia.
-Are the DMSO data in panel E the same data shown in panel D? If so, this should be explicitly stated.

Additional comments:

Fig 1A: Why is the graph duplicated?

Fig 1: The dose and duration of TPC2-A1-P administration needs to be stated.

Fig 1D: Is the analysis focused on only those cells over-expressing the ion channel? If so, how was this done? Are the disease specific difference consistent across multiple cell lines?

Fig 5: Are phenotypes consistent in more than one targeted clone?

Fig 7: Are the data in panels C, F, I repeated from panels B, E, H? It is unclear how they're different. For the quantification in panels C, F, and I, are the samples from separate blots? It seem that this would be problematic.

Fig 7L: What do the arrowheads show?

Referee #3 (Remarks for Author):

This is a dense manuscript with a large amount of data from multiple model systems and diseases and using diverse approaches. While the authors are commended for including so much information, this also presents challenges. In this case, unfortunately, incorporating such a large body of data into a single story has left lots of unresolved questions that need to be addressed, particularly regarding their animal work.

***** Reviewer's comments *****

Referee #1 (Remarks for Author):

The present ms explores an emerging approach for lysosomal storage disorders based on a new molecule that activates the two pore channel TPCN2. The work follows up on ground-breaking results published earlier by the same group (Gerndt et al., 2020 Elife), where they showed that the ion selectivity of the TPCN2 channel depends on the endogenous activator. Notably, the group identified two distinct small-molecule agonists that mimick the endogenous activators and thereby induce distinct functions of the channel in its natural habitat, the endosomal-lysosomal system. Here, the authors claim that one of these agonists, named TPC2-A1-P, shows beneficial effects in a range of cellular models of different lysosomal storage disorders, namely mucolipidosis IV, which is caused by genetic defects in a similar channel named TRPML1/MCOLN1. The authors have amassed an impressive amount of in vitro and in vivo results, and they provide a bit of in vivo data showing effects of the agonist on a mouse model of mucolipidosis type IV. The results could be of interest, but the ms has numerous severe flaws that preclude its publication. The following points are of concern:

A: We thank the reviewer for thoroughly reading our MS and for the many valuable comments to improve our MS. We also appreciate their comments that "The authors have amassed an impressive amount of in vitro and in vivo results". We feel that incorporating the reviewer's comments has now considerably strengthened our MS.

Major points

- The team's above-mentioned discovery of two distinct small molecule activators (TPC2-A1-P and -N; Gerndt et al., 2020 Elife, see also Gerndt et al., 2020 FEBS) would have provided a unique chance to explore which TPCN2 mode of function (ion selectivity) is required to mitigate disease-specific pathologic changes in the different models (or at least an informed selection of those). Unfortunately, it is not explained why the authors have chosen to test only the effects of TPC2-A1-P.

A: As shown in Gerndt et al., 2020, TPC2-A1-P but not TPC2-A1-N promotes lysosomal exocytosis, instead TPC2-A1-N strongly increases lysosomal pH, which would hamper lysosomal degradation. For these reasons we did not test TPC2-A1-N here. We also feel this would add an additional layer of complexity to an already overloaded MS. We hope the reviewer will find this acceptable.

- In all relevant figure panels, the authors must state what is represented by individual symbols in the graphs: cells/animals or means of preparations etc. The author must also state how many cells/preparations/micrographs etc., i.e. how many biological/technical replicates were used for the statistical analyses. The statement on pg. 13, para 6 ("was conducted on datasets where $n > 3$") is not sufficient, last not least because it is unclear what "n" represents (preps, animals, cells etc.).

A: We agree with the reviewer. We had to considerably shorten our MS when submitting to EMM due to space reasons set by the journal's guidelines. Nevertheless, we now provide this information in the respective figure legends.

- Pg. 4, para 3, Fig. 1A: The two graphs in this panel show exactly the same data except for distinct control (CTR) lines. Duplicate presentation of data is inacceptable.

A: We agree with this point and have removed one of the datasets.

- The authors' intention to test the effects of their drug candidate in different cell-based disease models is laudable and could provide important insight. Principally, one would assume that such drug tests are performed with the most suitable assay measuring pathologic accumulation of a disease-specific molecule(s). In the case of SMPD1- and NPC1-deficiency, for example, this would probably be sphingomyelin and cholesterol, respectively, in the case of mucolipidosis IV, the lactosylceramide-based assay can be a valid choice. However, throughout the Results part the authors use a bewildering range of assays without much justification/explanation of their choices and a clear lack of focus. Moreover, the results are shown in an inconsistent and eclectic manner. This must be completely revised including a redesign of figures. Examples to illustrate these points:

A: We agree with this point and have now restructured the MS. We explain now more precisely why we focus on MLIV, NPC1 and JNCL/Batten disease. We also explain why we use specific assays for one or the other LSD. For example, as the reviewer points out LacCer is well suited for MLIV analysis. Likewise, filipin assay works well in MLIV and particularly well in NPC1. Vice versa LacCer works also in NPC1. However, neither assay was suited for JNCL/Batten disease. We also would like to point out that LacCer or filipin while well suited for MLIV/NPC1 patient fibroblasts, appeared to be not or considerably less suitable as read outs for iPSC derived neurons. For example, we were not able to see a positive filipin staining in MLIV neurons and LacCer was not sufficiently endocytosed, precluding analysis of its intracellular trafficking routes. Instead, we had to use alternative assays for the analysis of iPSC derived neurons such as CathB. Activity or EM ultrastructural analysis (lamellar structures, mitochondria), the latter one being also successfully performed in fibroblasts . Therefore, different read outs had to be applied and explored for different cell types to some extent. In this context, we would like to draw the reviewer’s attention also to a new dataset on JNCL iPSC derived NPC, which show reduced Cristae numbers per mitochondrial area which can be rescued by TPC2-A1-P treatment (new Fig. 5H-I).

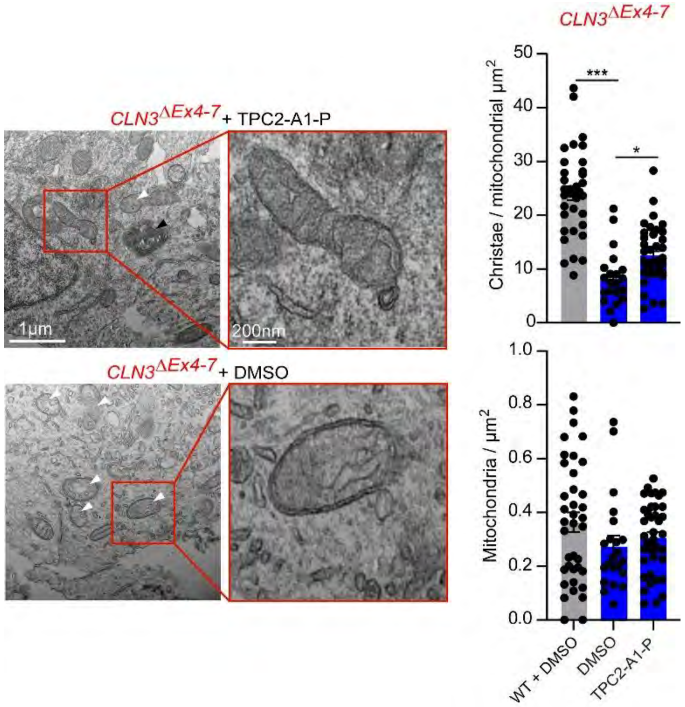

(1) The authors state on pg. 4, para 3 that they "assessed the effect of TPC2 activation in different LSD fibroblasts". However, it remains unclear, which fibroblasts/disease models were used and why and it is unclear why the results of these tests are not shown except for MLIV. This is inacceptable. It is important to see how the TPC2 agonist affects the different models with each given assay.

A: We agree with the reviewer and have now explained in more detail why we focus on MLIV, NPC1 and JNCL/Batten disease. We now also show more complete datasets as requested. We showed not all datasets in the first version in part due to limited space set by the guidelines of EMM and limited space is also a reason why we had to cut down considerably the figure legends incl. information on statistics. But we now provide detailed information on statistics, n numbers etc. in each figure legend. Instead, we have tried to shorten the MS elsewhere.

In the following sentence, the authors describe results from a distinct approach (overexpression of TPC2 and TRPML1 in three disease contexts, MLIV, NPC1, NPA). Again, there is no explanation why this approach was selected and why it is applied to three diseases. In the corresponding panels of Fig. 1, micrographs for MLIV and NPA are shown, whereas quantitative data are shown for three diseases. Again, it is unclear, why incomplete datasets are presented.

A: We have changed this now. We have removed all drug treatment data on NPA as this disease is not a focus of the MS. Instead more datasets are now provided on the LSDs we focused on, we also focused exclusively on TPC2 activation and removed data relating to ML1 activation as these data are not absolutely necessary for the story and only served as comparative control (nevertheless ML-SA1 was still used as a control for autophagy and exocytsosis assays).

(2) A similarly inconsistent presentation of results can be found on pg. 5-6 and Fig. 2 with respect to the filipin assay and ultrastructure. Again, the authors claim that effects of the

agonist were tested in different patient fibroblasts, but it is unclear in which ones, and the outcome is only shown for selected disease models, in the case of filipin staining for MLIV and NPC1, in the case of ultrastructure JNCL, MLIV and NPC1 for vehicle controls, and NPC1 for agonist effects. Here, ML-SA1 data are presented without explanation: why was the agonist used in this assay, but not in the other assays presented in preceding pages/figures?

A: Same as above. We have changed this now. We now focus on MLIV, NPC1 and NCL/Batten disease. We found that filipin works well in MLIV and NPC1 patient fibroblasts and we show rescue data for both diseases. JNCL fibroblasts showed no cholesterol-accumulating phenotype, hence TPC2-A1-P was not tested. The same applies to the EM data, where JNCL fibroblasts showed no phenotype in contrast to MLIV and NPC1. ML-SA1 was originally added as a positive control as it had been reported previously that activation of TRPML1 can rescue NPC1 phenotypes.

- Pgs. 4-6: Bioassays should be performed using different drug concentrations and durations of treatment to determine the full range of drug effects. The authors tested only a single concentration of their drug candidate (e.g. 30 µM of TPC2-A1-P; except for EV5) and they used treatment durations ranging from 2h (for the lactosyl-ceramide assay), 180 min (= 3h; autophagy assay) to 48-72h (lysotracker filipin staining) depending on the assay. The choice of these paradigms is not explained and one wonders about their validity. The reported EC50 values in Fluo-4 calcium experiments were in the 1-10µM range, whereas the EC50 values obtained by electrophysiological recordings were 10-fold lower. The authors' assumption that the use of a single concentration is sufficient to explore the effects of their drug in different lysosomal diseases with distinct mechanisms is not justified.

A: This is a very good point. The dose/concentration is based on the results in Gerndt et al., 2020 where 30 uM TPC2-A1-P showed the most robust effects in cellular/functional assays. Fluo-4 DRCs were generated with the PM variant of TPC2 in overexpression and EC50s cannot be expected to be the same in endogenous systems with channel expression in the lysosomal membrane. Patch clamp experiments were performed on isolated lysosomal membranes, i.e. concentrations for activation are expected to be considerably lower here as compound is directly perfused onto the isolated lysosomes.
Every cellular assay requires specific incubation times, which were empirically determined, thus e.g. we experimentally determined the ideal incubation time for the filipin assay, in which 16h of incubation was not sufficient to rescue MLIV while 24-48h incubation was found to be sufficient to rescue. In contrast, for NPC1 significant effects were only found after 48h incubation, in line with results obtained in the LacCer exp. We show now the respective time courses in new Fig. EV2A-B.

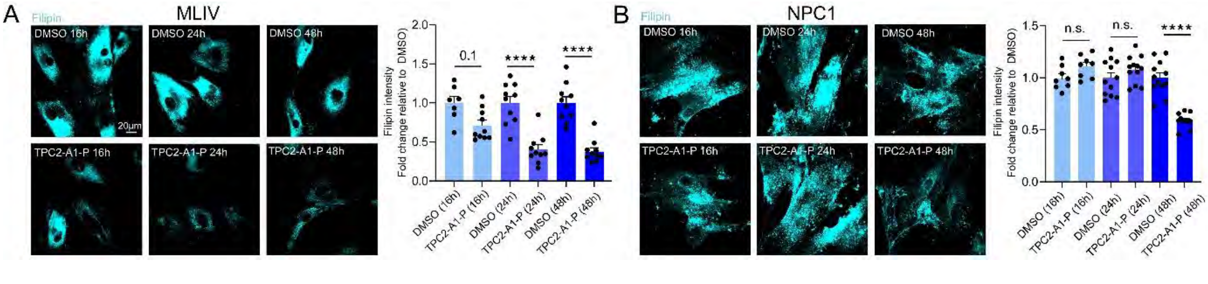

- Pg. 4-5, Fig. 1D-F, Fig. 2E-G: The authors tested drug effects on fibroblasts overexpressing wildtype versions of TPC2 or TRPML1 channels using different assays (LacCer

accumulation, filipin). However, the authors do not show the effects of channel expression with vehicle treatment. The outcome of these standard controls must be included.

A: We now performed additional exp. including channel overexpression with vehicle treatment only. See new Fig.1F-G and 2E-G.

Moreover, a description of the plasmids used to transfect cells must be given in the relevant sections of Material/Methods (pg. 11).

A: We now provide more detailed information in text and figure legend on plasmids used.

On pg. 4, para 3, the authors state that MLIV cells respond "strongly" to TPC2/TPC2-A1-P treatment (see Fig. 1D). This is only partially true, it appears that only a subset of cells (or preparations? See comment below) is affected. Does this depend on the level of channel expression?

A: We have toned this down now in the text. Channel expression efficacy may indeed play a role and explain the variation. While overexpression adds an additional effect clearly in some cells, it seems less prominent in others. Nevertheless, all transfected cells (mCherry+) were included into the statistical analysis.

- Pg. 5, para 1, Fig. 1G; Fig. 2H-I: It is unclear, why the authors show effects of BAPTA on LacCer and filipin distribution in "wildtype" fibroblasts. With respect to LacCer, this experiment cannot address the question why ion channel overexpression and activation in NPA disease fibroblasts failed to affect LacCer accumulation. It is further unclear to which effects authors refer to in the sentence "Hypothesizing that the observed effects were due to lysosomal Ca2+ release". With respect to cholesterol, the reason why these experiments were performed is not indicated. Since the relation of these experiments to the specific goals of the present study is unclear, they should probably removed.

A: We have clarified this now in the text. The BAPTA-AM experiments in new Fig.1 and new Fig. EV2 show that removal of intracellular Ca^{2+} mimics in CTR cells the phenotypes seen in LSD cells, i.e. accumulation of LacCer and cholesterol. We think this is an important information, suggesting removal of intracellular Ca^{2+} to be relevant for phenotype development. We would therefore argue to leave these data in the MS, while providing now a better explanation for them in the text.

- Pg. 5, para 1: The statement "Altered cellular cholesterol homeostasis is rather common in LSDs" is imprecise and should be removed. There is a large number of LSDs where cholesterol distribution is unaffected. The authors mention at least one, JNCL.

A: We have removed this statement.

- Pg. 5, para 1, EV1: The results shown in expanded view 1 are interesting, but only partially related to the present study. Moreover, they are introduced and explained in an insufficient manner. The fibroblasts are incompletely characterized, different agonists were used, and effects of TPC1-A1-P on IV curves are not shown.

A: We thought that showing additional patient lines would strengthen the MS and our conclusions because we see rescue effects across the panel of all tested MLIV mutants with

TPC2-A1-P. But we will follow the argument of the reviewer here that the data is only partially related to the study and removed this dataset accordingly.

- Pg. 5, para 1, EV2: The author show yet another series of experiments (siRNA-mediated silencing of TPC2) rather en passant without much explanation. Evidently, these results should be shown in a main figure 2, as target validation in vitro is an important element. On the other hand, the results are incomplete, as the validation is only shown with respect to NPC1 and filipin, not for example with respect to LacCer accumulation for example in MLIV fibroblasts related to experiments presented in Fig. 1. No explanation is given.

A: We agree that this dataset is important to demonstrate on-target effects. We have moved the filipin siRNA data for NPC1 to Fig.2 now. We have also added to new Fig. EV2 another dataset on siRNA confirming on target effect in LC3 experiments (see below). Finally, we show also siRNA effects in LacCer assays (see new Fig.1J-K).

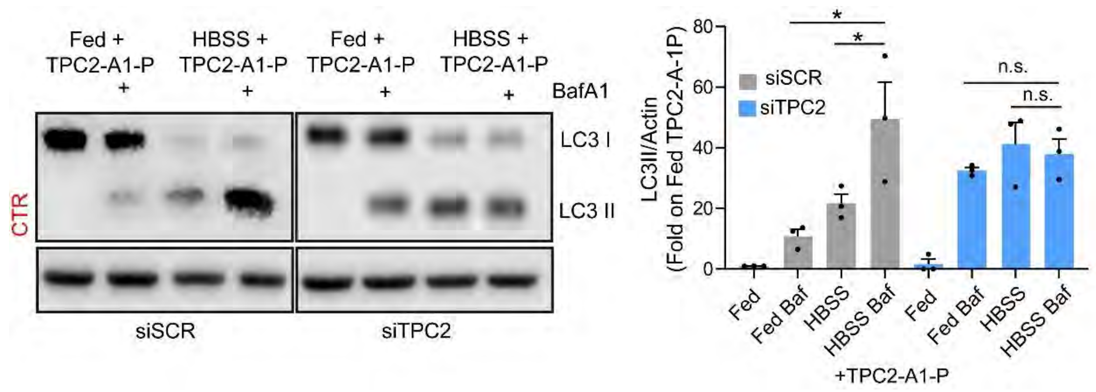

In EV2, the y axes label reads "Filipin-cholesterol load" but it is unclear, how this parameter was measured. In EV2B, values are normalized to fibroblasts treated with scrambled siRNA and with DMSO. However, is this possible, because one would expect that the cells treated with siTPC2 show an increased level of cholesterol load compared to siSCR, which then is reduced or not by TPC2-A1-P.

A: Filipin cholesterol load corresponds to filipin intensity that was measured at the excitation wavelength of 405 nm (emission 480 nm). We replaced the corresponding labels of the y axes with “Filipin intensity”. In NPC1 cells it would be expected that siRNA SCR with DMSO shows filipin accumulation, while treatment with TPC2-A1-P should reduce it in NPC1 cells (hence normalized to DMSO). In siRNA TPC2 treated NPC1 no effect of TPC2-A1-P is expected. Indeed TPC2-A1-P treatment is not different from DMSO treatment. And also no further increase was found (maximum already reached in NPC1 cells). By contrast, in CTR experiments it is shown that siRNA TPC2 treatment results in increased cholesterol, in accordance with findings in murine TPC2KO cells (Grimm et al., Nature Commun., 2014).

In EV2C, the abbreviation in the label of the y axes is not explained.

A: y axes should be labeled as “TPC2 relative expression” (further information in Materials and Methods). We have changed this.

- Pg. 6, para 1; pg. 8, para 1; Fig. 2J-L; Fig. 5H,I: Results obtained by electron microscopy are presented, but the ms lacks a description of the experimental procedures used and of the way quantitative data were obtained. This information must be provided.

A: We now provide a description of EM and EM analysis in the Methods section.

- Pg. 7, para 2; Fig. 4F,G: The authors show effects of their favorite agonists on channel IV curves and current densities, but these findings are not mentioned in the Results.

A: Apologies if this has not been clear enough. Indeed we mentioned these data (Fig.4F-G and also D-E) in the section with the headline "Effect of TPC2 activation in neurons derived from human LSD iPSCs."

- Pg. 7, para 2; Fig. 5A: It is unclear what is represented by the measurements shown in Panel A. Are these mean values obtained from n = ? independent preparations?

A: Shown in 5A is the MagicRed Cath.B intensity measured using FRAP analysis (see Methods section). Results are as reported in the literature (Metcalf et al., 2008 and Colletti et al., 2012), following the protocol described by Metcalf et al. (2008). These are mean values from 3 independent exp. Further clarified in the Fig. legend now.

- Pg. 7, para 2: The authors state "functionally we assessed these neurons by... ": It is doubtful whether a cathespin B activity assay can be used to analyse neuronal function. This statement should be revised and the following findings should be introduced properly.

A: We have revised this paragraph.

- Pg. 8, para 2: The authors propose to study lysosomal exocytosis as "potential rescue mechanism", which is a valid idea that has been explored previously. However, it remains unclear which "observed rescue effects" they refer to. Moreover, it is unclear why these data obtained with cultured fibroblasts are shown here and not in the preceding paragraphs where the "observed rescue effects" were presented ?

A: We have now removed some of these data (see below) and combined exocytosis and autophagy data in one Fig. In this Fig. we show both fibroblast and neuron data, justifying IOO to leave this Fig. positioned after the iPSC/neuronal model data.

- Pg. 8, para 2: The validity of macrophages as "an independent cellular model" cannot be estimated, because the authors have neither established the presence of the pathologic changes (e.g. accumulation of LacCer or cholesterol, disturbance of autofluorescence etc.) in these cells nor did they show that the agonists reduce these changes at the first place.

A: We have removed these data accordingly.

- Pg. 8, para 2: The authors propose autophagy as potential rescue mechanism. This appears a bit strange given that autophagy itself is disturbed in LSD diseases: How can a pathway that is perturbed serve as rescue mechanism? Again, the authors test this idea in selected fibroblasts of two diseases without explanation why in these models and not in others.

A: Disturbed autophagy flux in MLIV and NPC1 cells can be rescued by TRPML1 as demonstrated before or, as we show here by TPC2 activation. Both channels when activated promote autophagic flux and degradation. Indeed, autophagy activation through TRPML1 is dysfunctional in MLIV cells as expected (TRPML1 is absent or mutated) but TPC2 is fully

functional and can according to our results compensate for the defect. TPC2 mediated autophagy activation is impacted neither in CTR nor NPC1/MLIV cells.
We did not test autophagy in JNCL fibroblasts since under basal conditions (not mitomycin treated) no disease phenotype was identified in JNCL fibroblasts.

- Pg. 8, para 2/EV5: The authors' claim that "In TPC2 knockout fibroblasts, we observed no effect with TPC2-A1-P" is inaccurate and not supported by the data. The experiments were performed in a completely different manner than the ones shown in Fig. 7A-I. First, no fed-starved conditions were examined. Second, for unknown reasons, the authors used mouse embryonic fibroblasts (if this is what MEF stands for). Third, the experiments were performed without bafilomycin control, which is inacceptable for this kind of assays. Fourth, the authors employed a much lower agonist concentration (1-5µM EV5 compared to 30 µM Fig. 7). Moreover, the y-axis label is incorrect, as the values were obviously normalized, but it is unclear to what. The meaning of the results and their relation to experiments performed on "autophagic flux" are unclear.

A: We agree with the reviewer that using murine cells here may be less appropriate than using HF. We therefore repeated these experiments with HF (see also new Fig. EV2).

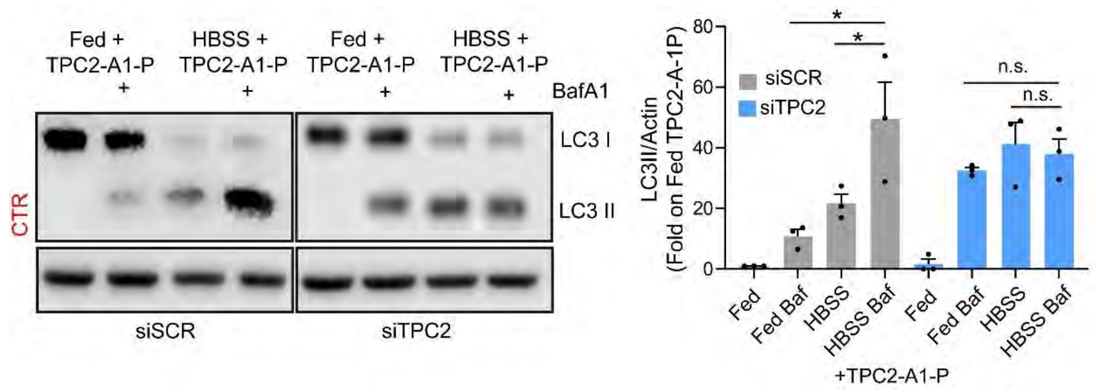

- Pg. 8, para 2; Fig. 7: iPSC-derived neurons were maintained under fed and starved conditions using two distinct media (Neurobasal versus DMEM, as indicated in the legend). Given their distinct compositions, can the authors exclude that their protocol induces cellular changes other than just those due to a fed-starved transition?

This is a very good question. Since we wanted to validate our data obtained in fibroblasts (increase in LC3-II band upon co-treatment with HBSS and TPC2-A1-P) in neurons we were searching for a "starvation" protocol appropriate for neuronal cells. Although autophagy is very relevant for neuronal function, there is limited literature on it. Classical methods for inducing autophagy in non-neuronal cells, like HBSS starvation, are too harsh and lead to neuronal cell death. By following the methods shown in Jessica E. Young et al 2009 (10.1074/jbc.M806088200) we used their nutrient-limited media (DMEM w/o B27, a supplement added to the Neurobasal to provide a variety of lipid-rich compounds, proteins, and growth factors). This approach has been shown to safely induce autophagy (LC3-II) in neurons.

- Pg. 8, para 2; Fig. 7: It is unclear based on which results shown in the figure the authors claim that "TPC2-A1-P recovered impaired autophagic flux in NPC1 and MLIV". In MLIV fibroblasts, the basal changes induced by starvation and by bafilomycin (E, F) and the effect

of the agonists are much smaller compared to those in WT and NPC1 fibroblasts (compare scales panel B, E, H).

A: The impairment of the autophagic flux is visible by comparing the two bafilomycin treated samples (in fed and Hbss): no significant difference is seen in both MLIV and NPC1 (DMSO treated). The result of this experiment is showing that there is no de novo LC3-II lipidation and even if autophagy was induced (via nutrient deprivation) there is no further increase in LC3-II, indicating an autophagic flux block. On the contrary, by co-treating nutrient deprived cells with TPC2-A1-P we have a significant difference between the two bafilomycin treated samples (fed and Hbss), suggesting a recovery in the autophagic flux. Analyses are done for each fibroblast species separately (MLIV, NPC1 etc.) and do not depend on each other. The densitometry values are different as different primary fibroblasts may present different LC3 levels (therefore, we calculated the amplitude of the autophagic flux specifically for each fibroblast sample, respectively).

The measurements of P62 levels were performed without the bafilomycin control, and therefore it is unclear to which degree the levels of P62 under the distinct condition depend on autophagic degradation.

A: We agree with the reviewer that bafilomycin controls are needed here. We now provide these missing controls, see new Fig. EV2 (exp. shown for NPC1 and MLIV, each). We found that P62 clearance is hampered under starvation plus TPC2-A1-P plus bafilomycin A1 treatment. The difference between HBSS+A1-P versus HBSS+A1-P+Baf remains highly significant ($p < 0.0001$). Nevertheless, we cannot fully exclude involvement of additional pathways or mechanisms.

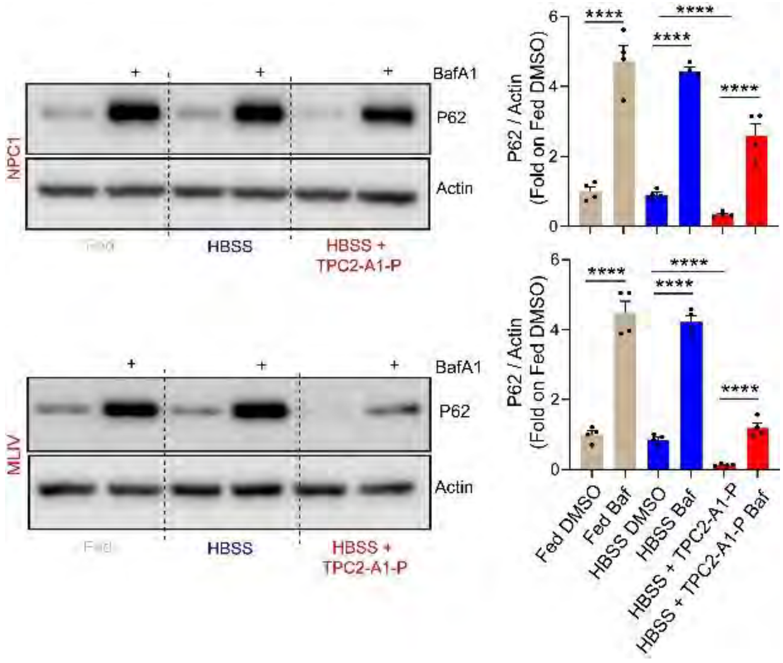

- Fig. 7: Graphs in panels C, F & I show the same data as presented in panels B, E & H. Duplicate presentation of results is inacceptable. In addition, it is not clear what the y axes show. Are the values normalized to some control conditions: the values seem to equal 1 in DMSO+Fed condition. If this is the case, the titles of the y axes must be corrected.

A: Apologies for the confusion, we thought that splitting the plots (condition plus bafilomycin to highlight the flux and condition w/o bafilomycin to show the effect of starvation) would facilitate data interpretation. We have now restructured the Fig. and avoid showing duplicate datasets. Every sample (CTR, NPC1 etc.) is normalized to the respective fed DMSO condition. We corrected the y axes accordingly.

- Fig. 7K: The y axis label states "Ctr fed dmso" but it is unclear what CTR refers to?! Wt? In this case, the first value is probably incorrect, as it should be 1.

A: Thanks for pointing this out. We have now corrected this.

- Pg. 8-9: The generation of a TPC2-IRES-Cre line is a strength of the study as this tool will be of interest for future investigations. The authors should probably note in this part of the Results that the outcome of recombination and thus the type of cells labeled and their percentages in distinct brain regions depends on the Cre-dependent reporter line used.

A: We thank the reviewer for this positive comment. We have added a sentence to clarify the comment on the Cre-dependent reporter line used: "The labeling of TPC2 positive cells via expression of tGFP is dependent on the expression of Cre recombinase under control of the TPC2 promotor."

- Pg. 9-10: The in vivo experiments are an important part of the study, but the methods used to test effects of the TPC2-A1-P on MLIV mice are not described in the Material/Methods section. Notably, a "statement in the Materials and Methods identifying the institutional and/or licensing committee approving the experiments" as demanded by EMBO (and any other journal) is missing both for the pharmacokinetic study on BL6 mice and for the TCP2 study on MLIV mice.

A: Pharmacokinetic studies were performed by Enamine/Bienta according to their study protocols as indicated in the Methods section. Regarding experiments with MLIV mice we have added the following paragraph in the Methods Suppl.: "Animals were used under animal protocols approved by the government (Regierung von Oberbayern, ROB-55.2-2532.Vet_02-17-170) and University of Munich (LMU) Institutional Animal Care Guidelines or in accordance with the guidelines and policies of the European Communities Council, approved by the Italian Ministry of Health. Mice were housed in rooms maintained at constant temperature (20-24°C) and humidity (45-65%) with a 12 hour light cycle. Animals were allowed food and water ad libitum."

- Pg. 9-10: With respect to the tests in MLIV mice it is key to know which vehicle has been used (DMSO-PEG400 as stated for the pharmacokinetic analysis) to deliver the agonist and whether the same has been used as vehicle control (DMSO or DMSO-PEG400). DMSO alone without PEG400 would be inacceptable.

A: We used the conditions as stated for the pharmacokinetic analysis.

- Pg. 9-10: In the same context, it is key to know whether littermates have been used as wildtype controls. Evidently, littermates represent the only valid control for this kind of studies.

A: Littermates were used in all experiments. Info added in the Fig. legend.

- Pg. 9-10; Fig. 9: The authors indicate the densities of IBA1- and GFAP-positive cells in different brain regions of MLIV and wildtpye animals treated with DMSO, but data from agonist-treated mice are only shown for one brain region (arbor vitae). This is inacceptable. Full datasets must be shown to judge the robustness of the effect across brain areas. Panel E repeats data shown in D.

A: We agree and now show the TPC2-A1-P effects for all conditions.

Other points

- The ms suffers from an inconsistent use of terms and abbreviations in figures and text. This must be revised. In the following, a few examples are mentioned: (1) Pg. 2, para 1 and throughout ms: The authors should use consistently the official symbol TPCN2 for the two-pore segment channel. (2) Pg. 4, para 3 and throughout the ms. The authors should use consistently the official abbreviation for TRPML1, which is MCOLN1. This includes also the designation of human gene mutations or protein variants. The parallel use of both terms is particularly confusing on pg. 7 and Fig. 4.

A: The terms TPC2 instead of TPCN2 and TRPML1 instead of MCOLN1 are widely accepted; indeed TRPML1, which belongs to the TRP channel family is now the preferred term. We mention now both terms, each, first in the MS to clarify that these terms are synonymous, but would prefer to use TPC2 and TRPML1 throughout the MS. For patient lines we would stick to the names as they appear in the databases of e.g., commercial providers such as the Coriell Institute.

(3) Throughout the ms and figures the authors use CTRL, CTR or wildtype. They should use consistently one term. With respect to fibroblasts from healthy donors, the term "wildtype" may not be appropriate.

A: We agree and have changed this to “CTR” throughout the MS.

(4) The abbreviation "HF" is used in some figures (e.g. Fig. 3, Expanded View) and not in others. This must be corrected.

A: We have corrected this. “HF” is omitted now in the Fig.

(5) On pg. 6 and Fig. 3 the terms JNCL and CNL3 are used, respectively. This should be corrected.

A: We have now corrected this. We use the term JNCL throughout the MS except for iPSC derived neurons, which we named according to their CRISPR/Cas engineered genotype.

- Pg. 3, para 1: For clarity the authors should add to the more historic term Niemann-Pick type A the correct nosology "infantile neurovisceral form of acid sphingomyelinase (SMPD1) deficiency" and use this subsequently in the text.

A: Added.

- Pg. 11 para 3 & Fig. 1A: The authors should explain whether analysis of colocalization was

performed on selected regions of interest containing cells or on the entire image. Evidently, the former is preferred.

A: Entire images were analysed.

- Pg. 12 (and elsewhere): Statements such as "immunofluorescence targeting LAMP1 protein is performed overnight" or "Autofluorescence mean intensity in the 488nm channel and 405 channel in LAMP1" are lab jargon and must be replaced by precise statements. The excitation and emission wavelengths to measure autofluorescence etc. must be stated clearly.

A: Corrected.

- Pg. 13, para 6: Replace "to-tailed" by "two-tailed".

A: Corrected.

- Pg. 19, para 1: The statement "which showed the most significant assay window" is unclear and should be replaced/removed.

A: Removed.

- Figures: The authors should consider to redesign their graphs and figures to render them more intelligible. For example, in Fig. 1, panel C shows two types of measurements: are the ranges of y axes really similar: density of puncta versus Mander's coefficient? Other example: panels E and F show micrographs whereas the quantitative data are presented already in D. Thus, the link between the panels is not obvious.

A: We have rearranged this where possible.

- Fig. 1B: The authors should consider to rearrange the panel and place MLIV treated with DMSO and with drug side-by-side.

A: Done.

- Fig. 1C: Here, and elsewhere, the term "spots" should probably be replaced by "puncta".

A: Replaced.

- Fig. 3, legend: The description of panels C and D is incorrect and must be revised. X axis designations (t0) and the y axis title are undefined.

A: Modified.

Referee #2 (Comments on Novelty/Model System for Author):

The authors present interesting findings on the effects of activation of TPC2 on the cellular phenotypes of a set of lysosomal storage disorders. Extensive data are provided demonstrating attenuation of aberrant lactosyl ceramide trafficking and cholesterol accumulation along with enhanced lysosome exocytosis among other changes associated with application of the TPC2 activator TPC2-A1-P. Pharmacokinetic studies and effects on some aspects of a mouse model of MLIV are also presented. The study builds on previous work by this group on TPC2 activation and work on TRPML1 and the effect of its activation on lysosomal storage disorder cellular pathophysiology. The effects of TPC2-A1-P on the is marked and reflects a potential for significant clinical impact on a subset of lysosomal storage disorders.

A: We thank the reviewer for their encouraging comments on our MS and that they deem our data having a "potential for significant clinical impact on a subset of LSDs".

Referee #2 (Remarks for Author):

There are a few minor issues with the manuscript.
- The authors make the statement "TPC2-A1-P treatment significantly decreased CtsB activity in iPSC-derived MLIV neurons, supporting its therapeutic relevance in MLIV". To this reviewer this is an overstatement - decreasing CtsB in a cell model does not support therapeutic relevance.

A: We agree and have rephrased this paragraph.

- The authors use "wild-type" for human cells derived from non-diseased control subjects - "control" would be more appropriate.

A: Both reviewer 1 and 2 have raised this point. We now call "WT" cells control (CTR) throughout the MS.

- There are is no method description for the EM and importantly for the analysis of figures - importantly, how many samples/grids were used for each experiment and were those collecting/analyzing images blinded to sample treatment?

A: Also reviewer 1 had noticed this. Thank you very much to both for drawing our attention to this. We have now added a detailed section on EM Materials and Methods and analysis. All analyses were performed in a double blinded manner.

- While TPC2-A1-P clearly acts on TPC2, confirming that at least one of the effects of TPC2-A1-P on one of the lysosomal storage disorder models is dependent up on TPC2 would help exclude an off target effect.

A: We provide such data in new Fig.2H-K for filipin and NPC1 (see below), in addition in new Fig.1J-K for LacCer and NPC1, and in Fig. EV2H-I for LC3.

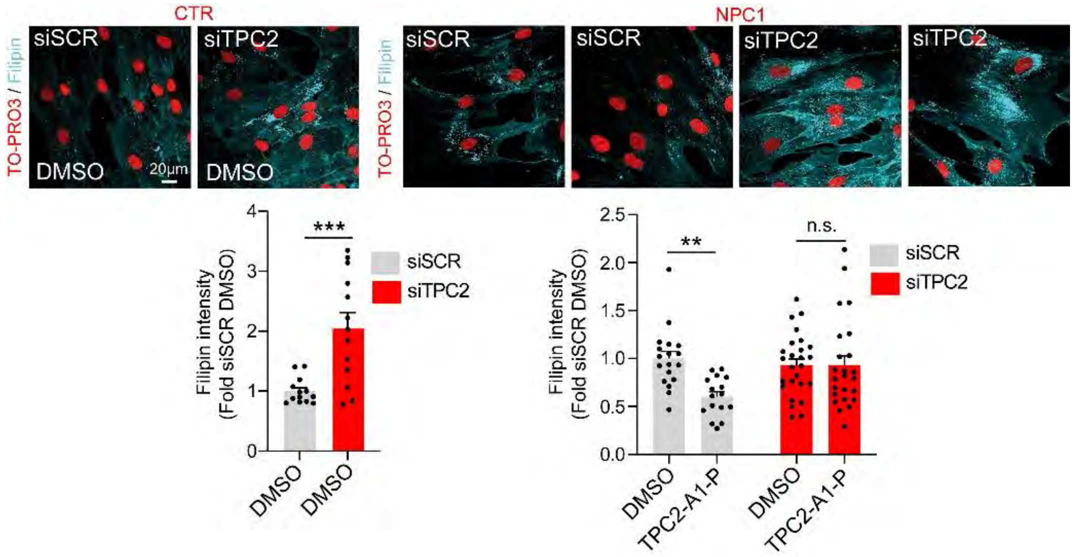

It would also be interesting to test whether BAPTA blunts the effect of TPC2-A1-P.

A: We did this experiment and indeed found that BAPTA blunts the effect of TPC2-A1-P. The data are now presented as part of Fig. EV2.

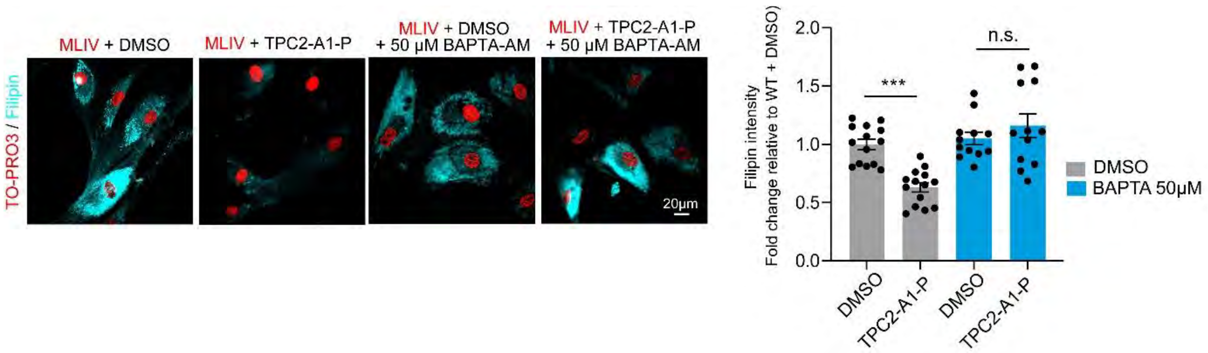

- A discussion about mechanisms to explain the differential effects on the different LSDs would be useful - e.g. why is Niemann Pick A lactosyl ceramide unaffected? Understanding the differential effects on different diseases could better the understanding of mechanism.

A: The lack of effect of both TRPML1 and TPC2 activation on NPA is indeed puzzling. While the lack of effect of TRPML1 activation may be explained by the fact that sphingomyelin blocks strongly TRPML1 activity, such an effect on TPC2 activity is not described in the literature. It may however be interesting to test this hypothesis. If TPC2 is also blocked by sphingomyelin, this may explain the lack of effects.
Since reviewer one asked to focus more, we removed the NPA data from the MS as we had generated data for NPA only for LacCer and did also not follow up on NPA throughout the MS.

Referee #3 (Comments on Novelty/Model System for Author):

Rosato et al explore the effects of small molecule activation of the endolysosomal two-pore channel 2 (TPC2) in diverse models of lysosomal disease, including patient fibroblasts and iPSC-derived neurons harboring mutations in several disease-causing genes as well as in MLIV mice. In aggregate, their data provide evidence of therapeutic benefit from activating TPC2 in a number of disease models by clearing storage material and promoting lysosomal exocytosis and autophagy. Initial data from MLIV mice also suggest a beneficial effect. These finding are reminiscent of the beneficial effects that have been reported for activation of TRPML1 and identify an additional potential target for therapeutic intervention. While these observations are of potential interest, several issues need to be addressed:

Scientific rigor requires attention. For the quantification of imaging studies in multiple figures involving cells and mice, it is unclear in graphs what each dot represents. It is not clearly stated how many experimental replicates were performed, how many cells per replicate were analyzed, or which statistical test was used to assess significance. F statistics should be included for ANOVA.

A: We thank the reviewer for carefully reading our MS. Due to space issues set by the guidelines of EMM we had to cut down the MS considerably during the first submission. We now added back the statistics and n number information in every Fig. legend.

The specificity of effects on TPC2-A1-P on cellular phenotypes (cholesterol, LacCer, lamellar structures) should be confirmed in TPC2 knockdown or null cells.

A: This is indeed an important point. We show such data in new Fig.2 on cholesterol rescue in NPC1 using TPC2 siRNA, in addition in new Fig.1 for LacCer and NPC1, and in Fig. EV2H-I for LC3.

Additionally, time- and dose-dependence should be shown.

A: This is an important point. The dose/concentration is based on the results in Gerndt et al., 2020 where 30 uM TPC2-A1-P showed the most robust effects in all cellular/functional assays.
Every cellular assay requires specific incubation times, which were empirically determined here, thus e.g. we experimentally determined the ideal incubation time for the filipin assay, where an incubation time of 24h was sufficient to rescue MLIV but not NPC1 cells (the latter ones accumulate more cholesterol than MLIV cells), while 48h incubation was found to be sufficient to rescue both. These data are now part of new Fig. EV2.

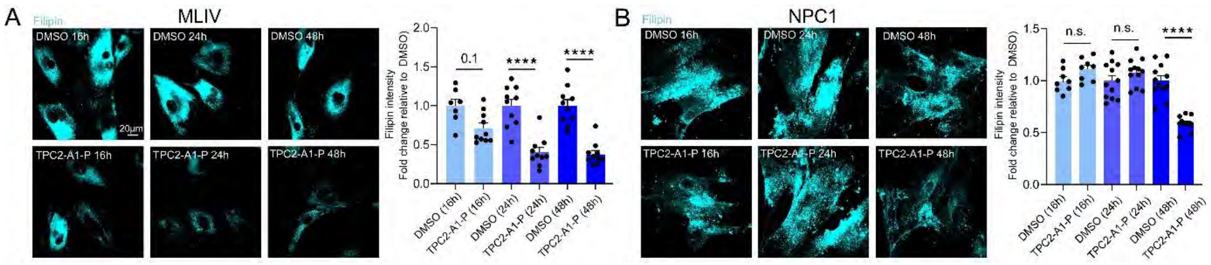

Therapeutic effects should be shown to occur without triggering cell death; this is particularly important for studies using iNeurons.

A: We have now performed cell viability assays for TPC2-A1-P in both fibroblasts and iPSC derived neurons (new Fig. EV1A-C), comparing them to other postulated TPC2 agonists.

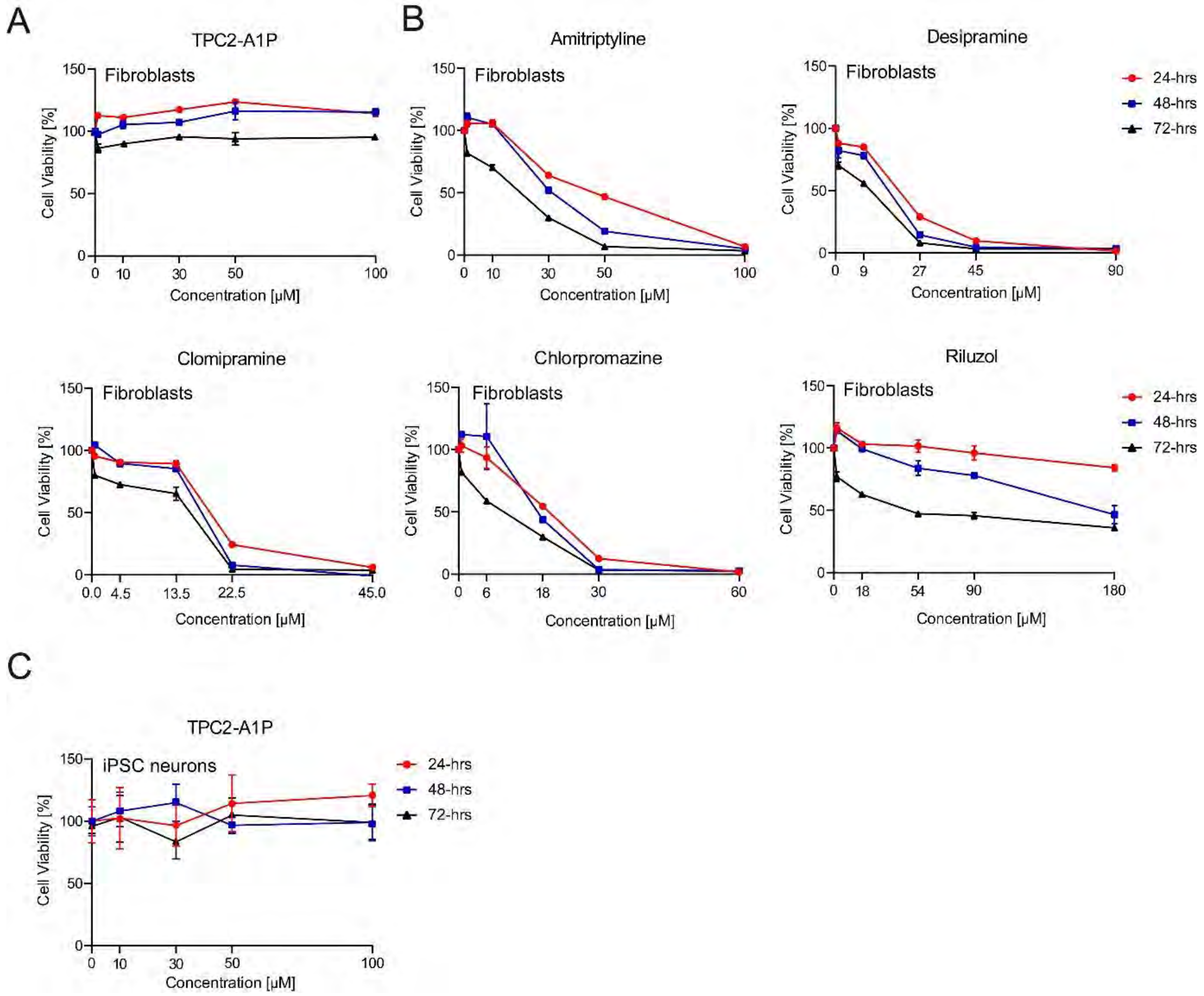

The animal studies reported in Fig 9 are under-developed and show only partial rescue of downstream components of the pathogenic cascade.
-Is there evidence that drug treatment alters lipid storage and behavioral phenotypes?

A: MLIV mice have been shown before to develop significant motor deficits already after 2 months of age (Walker and Montell, 2016). We have performed rescue experiments with TPC2-A1-P injections and measured the performance of vehicle versus compound treated MLIV mice in rotarod assays accordingly. See below and new Fig.8J. In addition, we tested the mice in open field experiments but found no significant assay windows between WT and MLIV mice. See below and new Fig.EV5C.

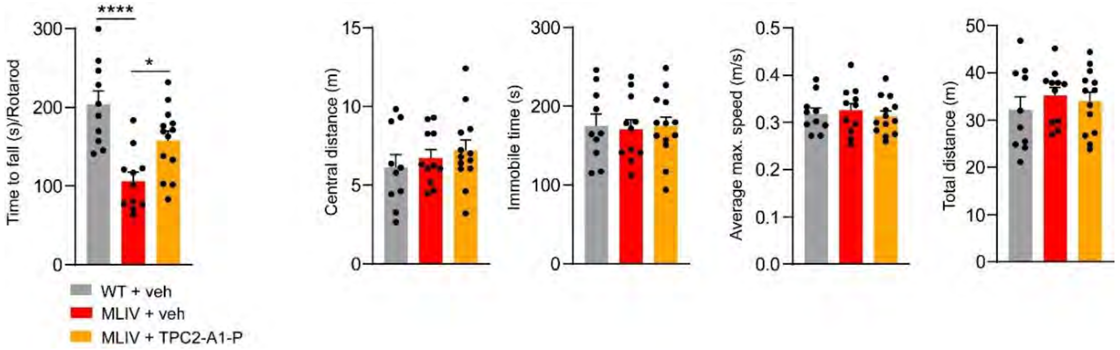

-A marker of target engagement in the CNS should be provided to confirm that rescue is not mediated by off target effects of drug administration.

A: This is a valid point, albeit we believe that target engagement has been shown convincingly by our in vitro data. We would certainly need to apply for a new animal protocol if we were to do the requested experiments, which would require approval by the Bavarian government (currently takes up to 10 months and more). In Germany the DFG, the ROB (Bavarian government) and other political entities strongly recommend to reduce any animal studies to the absolute minimum. Therefore, we have taken the effort here to generate over the last 3 years iPSC models for human LSDs. The advantage of this is two-fold. 1) we can work with human! neurons. 2) we avoid unnecessary animal experiments and stick to the recommended 3Rs principle:
"A crucial ethical guideline in animal experimentation is the 3Rs principle (Re- place, Reduce, Refine)1: Animal experiments may be performed only if no other suitable methods are available to investigate the research question and if the number of animals and the harms imposed on them are limited to the unavoidable". For further information see: www.dfg.de The 3Rs Principle and the Validity of Scientific Research.
We agree with the reviewer that this exp. may add additional confidence, but on the other hand believe that target engagement has been demonstrated convincingly, not justifying IOO additional animal experiments resulting in significant delay of the publication due to lengthy and unforeseeable administrative processes.

-Changes to autophagic markers should be corroborated by western blot.

A: Thanks for this suggestion. We tried to corroborate our P62 data obtained by immunofluorescence (IF) in cerebellum and hippocampus now by western blot. When we performed the in vivo experiments we perfused all the mice with 4% PFA. Instead of injecting new mice (see also Point above) we decided to make use of the already available brain slices from the areas of interest (cerebellum or hippocampus). We first washed the samples with PBS to remove tissue-tek residues. Next, we removed the PFA crosslinking by using an established protocol by Thacker et al., 2021 (https://doi.org/10.1016/j.jneumeth.2020.108995). We used high temperature (90˚C) in combination with 2% (w/v) SDS, everything in high molar TRIS buffer (500 mM). Unfortunately, we observed in contrast to the IF experiments no clear assay window between WT and MLIV. One possibility may be problems due to the processing of the samples as described above, alternatively, by using whole lysates from the brain slices we may dilute too much the effect observed in specific brain areas such as cerebellum or hippocampus. Nevertheless, the results are shown below for the reviewer's information.

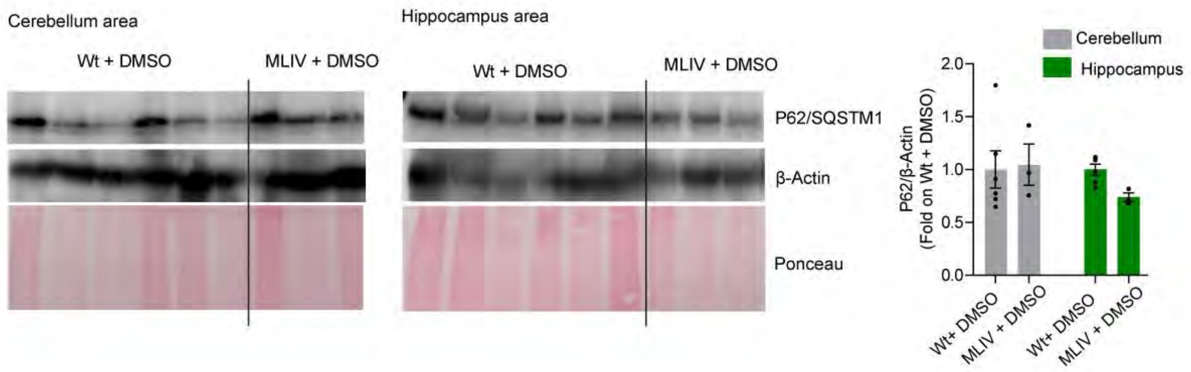

-Is analysis of the cerebellum from the same lobule? Many lysosomal models show a gradient of pathology across folia.

A: Yes, same lobule.

-Are the DMSO data in panel E the same data shown in panel D? If so, this should be explicitly stated.

A: Yes. We have modified the Fig. as to not show any duplicate data.

Additional comments:

Fig 1A: Why is the graph duplicated?

A: We have deleted the redundant data.

Fig 1: The dose and duration of TPC2-A1-P administration needs to be stated.

A: Done.

Fig 1D: Is the analysis focused on only those cells over-expressing the ion channel? If so, how was this done? Are the disease specific difference consistent across multiple cell lines?

A: The analysis was performed on the whole cell population. Disease specific differences are consistent across multiple cell lines. See previous version of the MS where several fibroblast lines e.g., for MLIV were presented. Since one reviewer advised to focus more, we have deleted these data from the MS.

Fig 5: Are phenotypes consistent in more than one targeted clone?

A: Is the reviewer referring here to fibroblasts or iPSCs and iPSC derived neurons? For fibroblasts the answer is "yes". Shown e.g. in original Fig. EV1, now removed as requested by reviewer 1. Regarding iPSCs: Single clones were selected after rigorous QC as they are isogenic to the control. Here, we did not test multiple clones as we selected single clones, that fully passed QC (including molecular karyotyping, on-target editing, etc. as described by Paquet *et al*., 2016 (https://pubmed.ncbi.nlm.nih.gov/27120160/) and Weisheit *et al*. (https://pubmed.ncbi.nlm.nih.gov/33597771/), rendering them identical to their isogenic

controls apart from the edited nucleotide(s).

Fig 7: Are the data in panels C, F, I repeated from panels B, E, H? It is unclear how they're different.

A: The Fig. has been rearranged and modified to avoid duplicate data.

For the quantification in panels C, F, and I, are the samples from separate blots? It seem that this would be problematic.

A: The data shown in C, F, and I are the same as in B, E, and H. They are all from the same experiments/blots. We originally intended, by dividing the plots, to facilitate data interpretation and to better visualize the results for the reader. We have now removed all duplicate data and we only show one single plot. We apologize for the confusion this has caused.

Fig 7L: What do the arrowheads show?

A: We removed the arrow heads as there is no need to leave them included.

Referee #3 (Remarks for Author):

This is a dense manuscript with a large amount of data from multiple model systems and diseases and using diverse approaches. While the authors are commended for including so much information, this also presents challenges. In this case, unfortunately, incorporating such a large body of data into a single story has left lots of unresolved questions that need to be addressed, particularly regarding their animal work.

A: We agree with the reviewer and have removed some datasets from the MS, which were either incomplete, e.g. NPA data or redundant such as duplicate data or the data in original Fig. EV1, showing TPC2-A1-P effects on different other MLIV fibroblast lines (this was specifically also a request by reviewer 1).

12th Jul 2022

Dear Prof. Grimm,

Thank you for the submission of your revised manuscript to EMBO Molecular Medicine. I am pleased to inform you that we will be able to accept your manuscript pending the following final amendments:

1) Please address all the points raised by the referee #1.
2) Please make sure that you implemented all suggestions made by our data editors on your previous version of the manuscript (see attached file).
3) We note that you currently have together with you, a total of 3 co-corresponding authors. Is that correct? Do you confirm equal contribution of these 3 people, able to take full responsibility for the paper and its content? While there is no limit per se to the number of co-corresponding authors, 3 is rare and may not reflect as intended to the community.
4) In the main manuscript file, please do the following:
- Reduce keywords to max. 5.
- Remove font colour.
- Make sure that the references to figures in the main text are correct. Add callouts for Figure 6D and E. There are callouts for Figures 7L-N and 9, which don't exist. All figure callouts should be in a sequential order, currently Figures EV3 and EV4 are called out before Fig EV2H-M. Also, Figure EV3 panels should be called out.
- In M&M, add statistical paragraph that should reflect all information that you have filled in the Authors Checklist, especially regarding randomization, blinding, replication.
- Please rename "Conflict of Interest" to "Disclosure Statement & Competing Interests". We updated our journal's competing interests policy in January 2022 and request authors to consider both actual and perceived competing interests. Please review the policy https://www.embopress.org/competing-interests and update your competing interests if necessary.
- Author contribution: The nature of every author's contribution must be specified both in the manuscript submission system (using the CRediT contributor role taxonomy) and in the manuscript under the heading "Author Contributions". Please check "Author Guidelines" for more information. https://www.embopress.org/page/journal/17574684/authorguide#authorshipguidelines
5) Author contributions: Please specify author contributions in our submission system. CRediT has replaced the traditional author contributions section because it offers a systematic machine readable author contributions format that allows for more effective research assessment. You are encouraged to use the free text boxes beneath each contributing author's name to add specific details on the author's contribution. More information is available in our guide to authors: https://www.embopress.org/page/journal/17574684/authorguide#authorshipguidelines
6) Funding: Please make sure that information about all sources of funding are complete in both our submission system and in the manuscript.
7) Synopsis:
- Please check your synopsis text and image and submit their final versions with your revised manuscript. Please be aware that in the proof stage minor corrections only are allowed (e.g., typos).
8) For more information: There is space at the end of each article to list relevant web links for further consultation by our readers. Could you identify some relevant ones and provide such information as well? Some examples are patient associations, relevant databases, OMIM/proteins/genes links, author's websites, etc...
9) Source data: We encourage you to include the source data for figure panels that show essential data. Numerical data should be provided as individual .xls or .csv files (including a tab describing the data). For blots or microscopy, uncropped images should be submitted (using a zip archive if multiple images need to be supplied for one panel). Please check "Author Guidelines" for more information. https://www.embopress.org/page/journal/17574684/authorguide#sourcedata
10) As part of the EMBO Publications transparent editorial process initiative (see our Editorial at http://embomolmed.embopress.org/content/2/9/329), EMBO Molecular Medicine will publish online a Review Process File (RPF) to accompany accepted manuscripts. This file will be published in conjunction with your paper and will include the anonymous referee reports, your point-by-point response and all pertinent correspondence relating to the manuscript. Let us know whether you agree with the publication of the RPF and as here, if you want to remove or not any figures from it prior to publication. Please note that the Authors checklist will be published at the end of the RPF.
11) Please provide a point-by-point letter INCLUDING my comments as well as the reviewer's reports and your detailed responses (as Word file).

I look forward to reading a new revised version of your manuscript as soon as possible.

Yours sincerely,

Zeljko Durdevic

Zeljko Durdevic

Editor
EMBO Molecular Medicine

***** Reviewer's comments *****

Referee #1 (Comments on Novelty/Model System for Author):

The authors have addressed the major points of criticism, the technical quality has greeatly improved and the novelty is more apparent. The model systems are adequate based on the explanations of the authors.

Referee #1 (Remarks for Author):

The authors have responded to the comments in a satisfactory manner, and the revision has improved the ms. The authors should address a few minor points that can be validated by the editors:

- Given that lysosomal calcium is a key topic according to the Introduction, the authors should also mention this in the abstract, which strangely lacks this term. This will help to render the work more accessible for humans and robots searching through bibliographic databases.

- The statements added by the authors at the beginning of the Results part (pg. 4, para 3) are very helpful, but they could be moved to the Introduction, so readers can immediately learn what the article is all about.

- On pg. 7, para 2, the following statement should be revised for clarity:
"We employed the endolysosomal patch-clamp technique (Chen et al, 2017), measuring TRPML1 currents with the TRPML agonist ML-SA1 in CTR neurons, but not in MCOLN1 IVS3-2A>G (MLIV) neurons". A suggestion would read: "Measuring TRMPL1-dependent currents using the endolysosomal patch-clamp technique showed their absence and presence in mutant and wildtype neurons, respectively".

- Fig. 6D. It's unclear, whether and how this "cartoon" can represent lysosomal exocytosis. This probably requires more work (or elimination). In case the referee missed this point in the first version, apologies for the "late comment".

Referee #3 (Comments on Novelty/Model System for Author):

This is an interesting report documenting the effects of a TCP2 agonist in cellular and mouse models of lysosomal diseases. I think it will be of interest to the LSD community.

Referee #3 (Remarks for Author):

The authors have done a very good job responding to my previous review. The revised manuscript has incorporated changes that make it much improved.

The authors performed the requested editorial changes.

We are pleased to inform you that your manuscript is accepted for publication and is now being sent to our publisher to be included in the next available issue of EMBO Molecular Medicine.

EMBO Press Author Checklist

Corresponding Author Name: Prof. Dr. Dr. Christian Grimm	
Journal Submitted to: EMM	
Manuscript Number: EMM-2021-15377-V3	

USEFUL LINKS FOR COMPLETING THIS FORM
The EMBO Journal - Author Guidelines
EMBO Reports - Author Guidelines
Molecular Systems Biology - Author Guidelines
EMBO Molecular Medicine - Author Guidelines

Reporting Checklist for Life Science Articles (updated January 2022)
This checklist is adapted from Materials Design Analysis Reporting (MDAR) Checklist for Authors. MDAR establishes a minimum set of requirements in transparent reporting in the life sciences (see Statement of Task: 10.31222/osf.io/9sm4x). Please follow the journal's guidelines in preparing your manuscript.
Please note that a copy of this checklist will be published alongside your article.

Abridged guidelines for figures
1. Data
The data shown in figures should satisfy the following conditions:
- the data were obtained and processed according to the field's best practice and are presented to reflect the results of the experiments in an accurate and unbiased manner.
- ideally, figure panels should include only measurements that are directly comparable to each other and obtained with the same assay.
- plots include clearly labeled error bars for independent experiments and sample sizes. Unless justified, error bars should not be shown for technical replicates.
- if n<5, the individual data points from each experiment should be plotted. Any statistical test employed should be justified.
- Source Data should be included to report the data underlying figures according to the guidelines set out in the authorship guidelines on Data Presentation.

2. Captions
Each figure caption should contain the following information, for each panel where they are relevant:
- a specification of the experimental system investigated (eg cell line, species name).
- the assay(s) and method(s) used to carry out the reported observations and measurements.
- an explicit mention of the biological and chemical entity(ies) that are being measured.
- an explicit mention of the biological and chemical entity(ies) that are altered/varied/perturbed in a controlled manner.
- the exact sample size (n) for each experimental group/condition, given as a number, not a range;
- a description of the sample collection allowing the reader to understand whether the samples represent technical or biological replicates (including how many animals, litters, cultures, etc.).
- a statement of how many times the experiment shown was independently replicated in the laboratory.
- definitions of statistical methods and measures:
  - common tests, such as t-test (please specify whether paired vs. unpaired), simple χ2 tests, Wilcoxon and Mann-Whitney tests, can be unambiguously identified by name only, but more complex techniques should be described in the methods section;
  - are tests one-sided or two-sided?
  - are there adjustments for multiple comparisons?
  - exact statistical test results, e.g., P values = x but not P values < x;
  - definition of 'center values' as median or average;
  - definition of error bars as s.d. or s.e.m.

Please complete ALL of the questions below.
Select "Not Applicable" only when the requested information is not relevant for your study.

Materials

Newly Created Materials	Information included in the manuscript?	In which section is the information available? (Reagents and Tools Table, Materials and Methods, Figures, Data Availability Section)
New materials and reagents need to be available; do any restrictions apply?	Yes	Materials and Methods, no restrictions

Antibodies	Information included in the manuscript?	In which section is the information available? (Reagents and Tools Table, Materials and Methods, Figures, Data Availability Section)
For **antibodies** provide the following information:<br>- Commercial antibodies: RRID (if possible) or supplier name, catalogue number and or/clone number<br>- Non-commercial: RRID or citation	Yes	Materials and Methods, Suppl.

DNA and RNA sequences	Information included in the manuscript?	In which section is the information available? (Reagents and Tools Table, Materials and Methods, Figures, Data Availability Section)
**Short novel DNA or RNA including primers, probes**: provide the sequences.	Yes	Materials and Methods, Suppl.

Cell materials	Information included in the manuscript?	In which section is the information available? (Reagents and Tools Table, Materials and Methods, Figures, Data Availability Section)
**Cell lines:** Provide species information, strain. Provide accession number in repository **OR** supplier name, catalog number, clone number, and/**OR** RRID.	Yes	Materials and Methods, Figures, Suppl.
**Primary cultures:** Provide species, strain, sex of origin, genetic modification status.	Yes	Materials and Methods, Figures, Suppl.
Report if the cell lines were recently **authenticated** (e.g., by STR profiling) and tested for mycoplasma contamination.	Not Applicable	

Experimental animals	Information included in the manuscript?	In which section is the information available? (Reagents and Tools Table, Materials and Methods, Figures, Data Availability Section)
**Laboratory animals or Model organisms:** Provide species, strain, sex, age, genetic modification status. Provide accession number in repository **OR** supplier name, catalog number, clone number, **OR** RRID.	Yes	Materials and Methods, Figures, Suppl.
**Animal observed in or captured from the field:** Provide species, sex, and age where possible.	Not Applicable	
Please detail **housing and husbandry conditions**.	Yes	Materials and Methods, Figures, Suppl.

Plants and microbes	Information included in the manuscript?	In which section is the information available? (Reagents and Tools Table, Materials and Methods, Figures, Data Availability Section)
**Plants:** provide species and strain, ecotype and cultivar where relevant, unique accession number if available, and source (including location for collected wild specimens).	Not Applicable	
**Microbes:** provide species and strain, unique accession number if available, and source.	Not Applicable	

Human research participants	Information included in the manuscript?	In which section is the information available? (Reagents and Tools Table, Materials and Methods, Figures, Data Availability Section)
If collected and within the bounds of privacy constraints report on age, sex and gender or ethnicity for all study participants.	Not Applicable	

Core facilities	Information included in the manuscript?	In which section is the information available? (Reagents and Tools Table, Materials and Methods, Figures, Data Availability Section)
If your work benefited from core facilities, was their service mentioned in the acknowledgments section?	Not Applicable	

Design

Study protocol	Information included in the manuscript?	In which section is the information available? (Reagents and Tools Table, Materials and Methods, Figures, Data Availability Section)
If study protocol has been **pre-registered, provide DOI in the manuscript**. For clinical trials, provide the trial registration number **OR** cite DOI.	Not Applicable	
Report the **clinical trial registration number** (at ClinicalTrials.gov or equivalent), where applicable.	Not Applicable	

Laboratory protocol	Information included in the manuscript?	In which section is the information available? (Reagents and Tools Table, Materials and Methods, Figures, Data Availability Section)
Provide DOI OR other citation details if **external detailed step-by-step protocols** are available.	Not Applicable	

Experimental study design and statistics	Information included in the manuscript?	In which section is the information available? (Reagents and Tools Table, Materials and Methods, Figures, Data Availability Section)
Include a statement about **sample size** estimate even if no statistical methods were used.	Yes	Materials and Methods, Figures
Were any steps taken to minimize the effects of subjective bias when allocating animals/samples to treatment (e.g. **randomization procedure**)? If yes, have they been described?	Yes	Materials and Methods, Figures
Include a statement about **blinding** even if no blinding was done.	Not Applicable	
Describe **inclusion/exclusion criteria** if samples or animals were excluded from the analysis. Were the criteria pre-established?<br><br>If sample or data points were omitted from analysis, report if this was due to attrition or intentional exclusion and provide justification.	Not Applicable	
For every figure, are **statistical tests** justified as appropriate? Do the data meet the assumptions of the tests (e.g., normal distribution)? Describe any methods used to assess it. Is there an estimate of variation within each group of data? Is the variance similar between the groups that are being statistically compared?	Yes	Materials and Methods, Figures

Sample definition and in-laboratory replication	Information included in the manuscript?	In which section is the information available? (Reagents and Tools Table, Materials and Methods, Figures, Data Availability Section)
In the figure legends: state number of times the experiment was **replicated** in laboratory.	Yes	Materials and Methods, Figures
In the figure legends: define whether data describe **technical or biological replicates**.	Yes	Materials and Methods, Figures

Ethics

Ethics	Information included in the manuscript?	In which section is the information available? (Reagents and Tools Table, Materials and Methods, Figures, Data Availability Section)
Studies involving **human participants**: State details of **authority granting ethics approval** (IRB or equivalent committee(s), provide reference number for approval.	Not Applicable	
Studies involving **human participants**: Include a statement confirming that **informed consent** was obtained from all subjects and that the experiments conformed to the principles set out in the WMA Declaration of Helsinki and the Department of Health and Human Services Belmont Report.	Not Applicable	
Studies involving **human participants:** For publication of **patient photos**, include a statement confirming that consent to publish was obtained.	Not Applicable	
Studies involving experimental **animals**: State details of **authority granting ethics approval** (IRB or equivalent committee(s), provide reference number for approval. Include a statement of compliance with ethical regulations.	Yes	Materials and Methods, Figures
Studies involving **specimen and field samples:** State if relevant **permits** obtained, provide details of authority approving study; if none were required, explain why.	Not Applicable	

Dual Use Research of Concern (DURC)	Information included in the manuscript?	In which section is the information available? (Reagents and Tools Table, Materials and Methods, Figures, Data Availability Section)
Could your study fall under dual use research restrictions? Please check biosecurity documents and list of **select agents and toxins** (CDC): https://www.selectagents.gov/sat/list.htm	Not Applicable	
If you used a select agent, is the security level of the lab appropriate and reported in the manuscript?	Not Applicable	
If a study is subject to dual use research of concern regulations, is the name of the **authority granting approval and reference number** for the regulatory approval provided in the manuscript?	Not Applicable	

Reporting

The MDAR framework recommends adoption of discipline-specific guidelines, established and endorsed through community initiatives. Journals have their own policy about requiring specific guidelines and recommendations to complement MDAR.

Adherence to community standards	Information included in the manuscript?	In which section is the information available? (Reagents and Tools Table, Materials and Methods, Figures, Data Availability Section)
State if relevant guidelines or checklists (e.g., **ICMJE, MIBBI, ARRIVE, PRISMA**) have been followed or provided.	Not Applicable	
For **tumor marker prognostic studies,** we recommend that you follow the **REMARK** reporting guidelines (see link list at top right). See author guidelines, under 'Reporting Guidelines'. Please confirm you have followed these guidelines.	Not Applicable	
For **phase II and III randomized controlled trials**, please refer to the **CONSORT** flow diagram (see link list at top right) and submit the CONSORT checklist (see link list at top right) with your submission. See author guidelines, under 'Reporting Guidelines'. Please confirm you have submitted this list.	Not Applicable	

Data Availability

Data availability	Information included in the manuscript?	In which section is the information available? (Reagents and Tools Table, Materials and Methods, Figures, Data Availability Section)
Have **primary datasets** been deposited according to the journal's guidelines (see 'Data Deposition' section) and the respective accession numbers provided in the Data Availability Section?	Not Applicable	
Were **human clinical and genomic datasets** deposited in a public access-controlled repository in accordance to ethical obligations to the patients and to the applicable consent agreement?	Not Applicable	
Are **computational models** that are central and integral to a study available without restrictions in a machine-readable form? Were the relevant accession numbers or links provided?	Not Applicable	
If publicly available data were reused, provide the respective **data citations in the reference list**.	Not Applicable	
